# Circular Cross-Linked Polyethylene Enabled by In-Chain Ketones

**DOI:** 10.1021/acsmacrolett.4c00660

**Published:** 2024-11-15

**Authors:** Tobias O. Morgen, Stefan Mecking

**Affiliations:** Chair of Chemical Materials Science, University of Konstanz, Department of Chemistry, Universitätsstraße 10, 78457 Konstanz, Germany

## Abstract

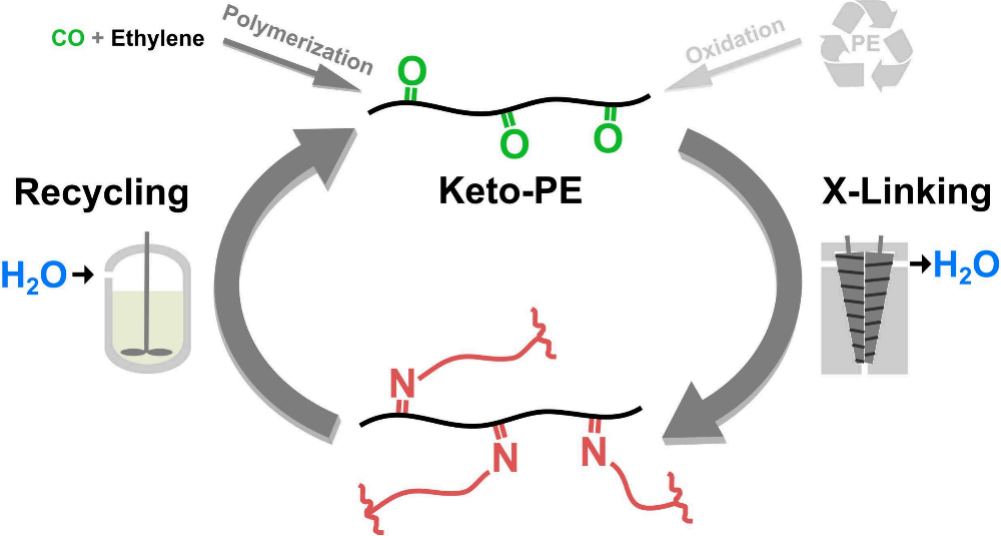

Cross-linked polyethylenes (PEs) are widely employed, but the permanent links between the chains impede recycling. We show that via imine formation with diamines keto-functionalized polyethylenes from both free-radical (keto-low-density PE, keto-LDPE) and catalytic (keto-high-density PE, keto-HDPE) nonalternating ethylene-CO copolymerization can be cross-linked efficiently in the melt, resulting in gel fractions of the formed cross-linked PEs of up to 85% and improved tensile properties. The imine-based cross-links in the material can be hydrolyzed at 140 °C to recycle up to 97% of the initial thermoplastic keto-polyethylene. Low keto contents of ≤1.5 mol % are found ideal to retain PE-like thermal properties, achieve sufficient cross-link density, and maintain circular recyclability.

Their cross-linked three-dimensional molecular structure imparts thermoset polymers with desirable properties, like mechanical strength and solvent resistance.^1^ Hence, about 20% of worldwide polymeric materials manufactured today (ca. 80 million tons per year) are thermosets.^2,3^ Although produced in such quantities, their recyclability poses severe obstacles since they cannot be easily fused or dissolved. Consequently, most thermoset materials after their useful service life are deposited in landfills or are incinerated. Higher value reutilization of this waste is desirable in the sense of a resource-saving circular economy.^4−9^

Among thermosets, recycling of cross-linked polyethylene (PE) is particularly challenging due to the inert nature of the hydrocarbon chains and the irreversible nature of the bonds generated by traditional cross-linking chemistry.^1,10−14^ This is a relevant problem as cross-linked PEs are employed on a multimillion-ton scale in the form of cross-linked low-density as well as high-density polyethylene, with the latter predominating.^15^ Elegant possible approaches to this problem through dynamic reversible cross-links have been reported recently.^16−25^ The introduction of the required reactive moieties to the polyethylene chain often utilizes reagents like azides,^16^ maleimides,^17−19^ or diazirines^20,21^ that require costly and laborious multistep synthesis or proceeds with a low efficiency. Some approaches cross-link ethylene copolymers which, however, contain very specialized comonomers^22,24^ and/or yield non-PE-like materials.^24,25^

Carbonyl groups offer themselves for a reversible cross-link chemistry as illustrated for numerous polymer systems,^26−31^ but access to in-chain carbonyl-functionalized polyolefins has been limited. This picture was recently altered by the finding of catalytic nonalternating ethylene-CO copolymerization as well as selective catalytic oxidation of polyethylene,^32,33^ both complementary methods affording linear keto-PE under mild conditions.

We report how polyethylenes with low in-chain keto content (≤2%) can be reversibly cross-linked with commercial diamines at relatively low temperatures (≤160 °C). We demonstrate the feasibility of this method both for keto-functionalized low-density PE (keto-LDPE) from free-radical^34^ and high-density PE (keto-HDPE) from catalytic nonalternating ethylene-CO copolymerization.^35^ The obtained imino-cross-linked PEs exhibit gel contents like commercial cross-linked PEs (≥65 wt %)^36,37^ and show improved mechanical properties. Hydrolysis of the cross-links under comparatively mild conditions allows for efficient recycling of the initial thermoplastic keto-PEs (Figure 1).

**Figure 1:**
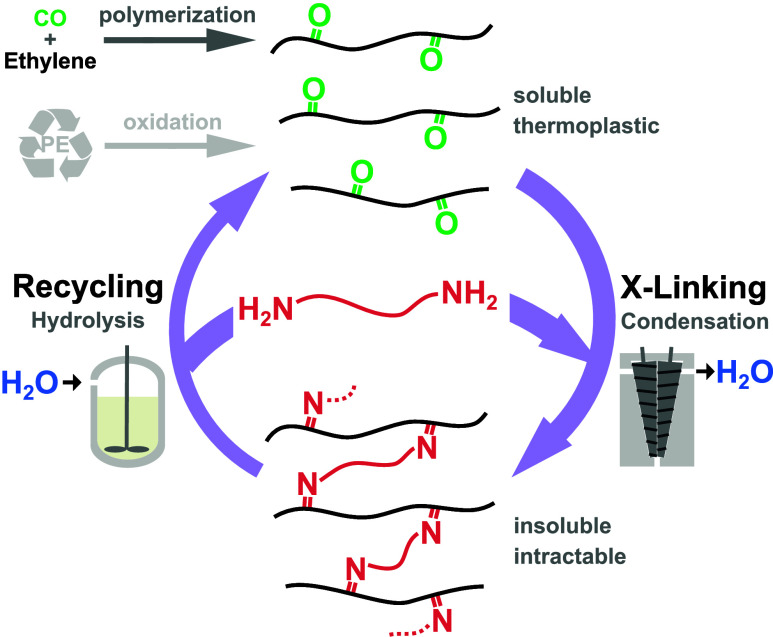
Concept of reversible cross-linking of keto-polyethylene comprising condensation with diamines and subsequent chemical recycling by hydrolysis of imines.

## Model Reactions with Low-Molecular-Weight Compounds

**Scheme 1:**
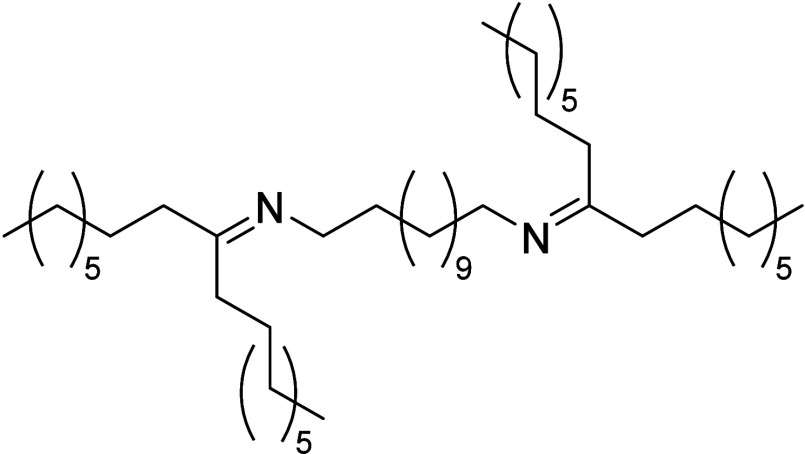
Chemical Structure of the Aliphatic Diimine *N*,*N*′-(Dodecane-1,12-diyl)bis(heptadecan-9-imine) Used As a Spectroscopic Reference for Imino-Cross-Linked PE

An aliphatic imine as a spectroscopic reference compound was synthesized from 1,12-diaminododecane and dioctyl ketone (cf. Supporting Information, SI, for details). Mixtures of the obtained liquid diimine product (Scheme 1) with dioctyl ketone were used to calibrate the ATR-IR band intensities of keto vs imino groups in an otherwise hydrocarbon medium (cf. SI for further details). This enables quantification of the imino:keto ratio and determination of the cross-link densities of the target cross-linked polymers (vide infra), other methods being prohibited by their insolubility.

The hydrolysis of this compound at room temperature under atmospheric moisture was so distinct that it had to be handled under inert gas. In slightly acidic water (pH 3), complete hydrolysis was observed within seconds at 95 °C. In contrast, at the same temperature even in a large excess of unbuffered water (4500 equiv) the hydrolysis reaction ceased after 15 min due to increasing pH by dissolution of formed amine.

Both amino and imino groups are sensitive toward oxidation by oxygen at elevated temperatures.^38^ Aliphatic imines are primarily oxidized in the α position to form α-keto imino groups which, in the presence of amine, further react to α-diimines (cf. SI for more information). Condensation and hydrolysis reactions on polymer melts and solutions were therefore performed under an inert atmosphere, which is also not an uncommon procedure in industrial processing of polymers.^39^

## Condensation of Keto-PE with Monoamine

**Figure 2:**
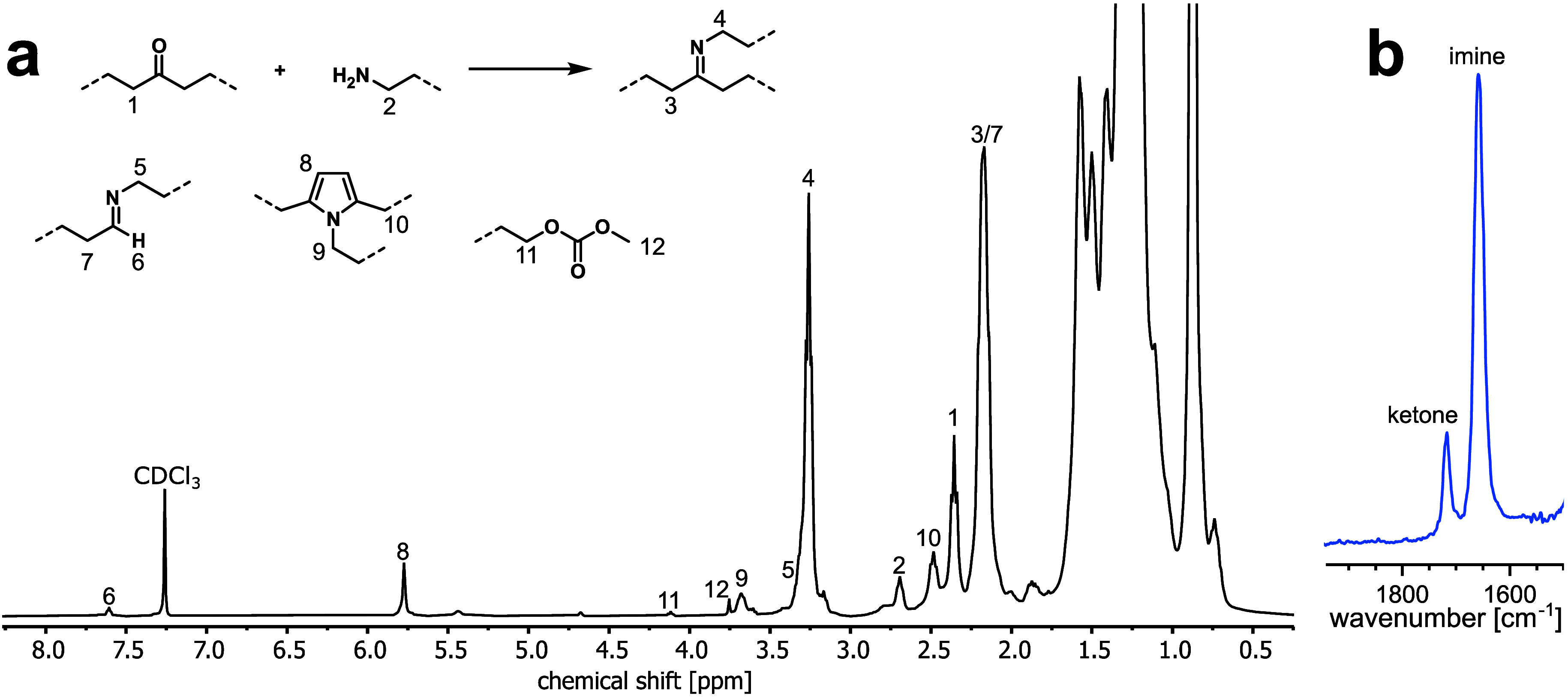
**a:**^1^H NMR spectrum (300 K, CDCl_3_) of imino-functionalized LDPE (5.8 mol % imino, 0.3 mol % pyrrole, 1.2 mol % keto) obtained from the condensation of keto-LDPE (7.6 mol % keto) and 1-aminotetradecane at 160 °C (cf. Table S3 in SI, entry I3). **b:** IR spectrum of the same product mixture (cf. Figure S15 for the full spectrum).

For detailed microstructure analysis by NMR spectroscopy, non-cross-linked LDPEs with imino groups were prepared from 1-aminotetradecane and keto-LDPEs from free-radical ethylene/CO copolymerization (cf. SI for details on synthesis and characterization).^34^ Polymers with ≤2 mol % imino groups compared to ethylene repeat units in the polymer were semicrystalline but with lowered melting points (ca. 100 °C) compared to initial keto-LDPEs. However, spectroscopic analysis of these polymers in solution was impeded by their low stability under conditions required for high-resolution NMR of PEs (C_2_D_2_Cl_4_, 100 °C). Samples with higher imino content were oily and soluble in CDCl_3_ at rt. NMR analysis shows that up to 83% of keto groups were converted to the desired imino functionality when 4 equiv of amine were used in the condensation reaction (Figure 2). 1,4-Diketone motifs form 2,5-disubstituted pyrroles with the amine (Paal–Knorr reaction).^40,41^

Otherwise, there are no indications of undesired side reactions. Neither chain scission nor aldol addition/condensation is evident, and the total number of functional groups in the material is unaltered. Note that all imino contents given are corrected by the number of additional methylene groups introduced into the material by the aliphatic amines. Therefore, functional group densities in imino-cross-linked PEs are directly comparable to those of the respective keto-PEs.

The functional group densities of imino-functionalized polymers were also quantified by ATR-IR spectroscopy (cf. Figure S27 in SI). In contrast to low-molecular-weight aliphatic compounds containing keto and imino groups, band deconvolution of polymer spectra called for four instead of two Lorentzian functions (two each for keto and imino groups, respectively). This effect is well-known from spectra of keto-PEs^34,42^ and is evoked by the spacial proximity of some polar functional groups which, compared to an isolated group in an all hydrocarbon medium, gradually decreases their resonance frequencies (from 1718 to 1700 cm^–1^ for keto and from 1659 to 1640 cm^–1^ for imino). Residual unreacted amino groups hinder quantification of imino groups by IR due to overlapping bands with virtually identical spectral position. In general, pyrrole motifs could not be unambiguously identified in IR spectra because 1) their concentration in all the samples was low and 2) their characteristic absorption bands overlap with those of imines and amines.^43^

## Cross-Linking of Keto-PE with Diamine

Preliminary small-scale cross-linking experiments of keto-LDPEs with 1,12-diaminododecane were conducted by melting the components at 160 °C, first under inert gas and then under high vacuum. While an excess of monoamine could be completely removed in vacuo, excess of diamine always resulted in cross-linked materials with remaining amino functionalities. We then aimed to avoid unreacted amines in the material because 1) they disturb PE crystallization and lower the materials hydrophobicity, in short, render it less PE-like without contributing to the desired cross-linking network, and 2) they prevent quantification of the conversion of ketones to imines by IR (vide supra). Typically, 0.95–1.00 equiv of NH_2_:CO was optimum to obtain imino-cross-linked LDPEs with little to no residual amino groups and sufficient cross-link density.

**Figure 3:**
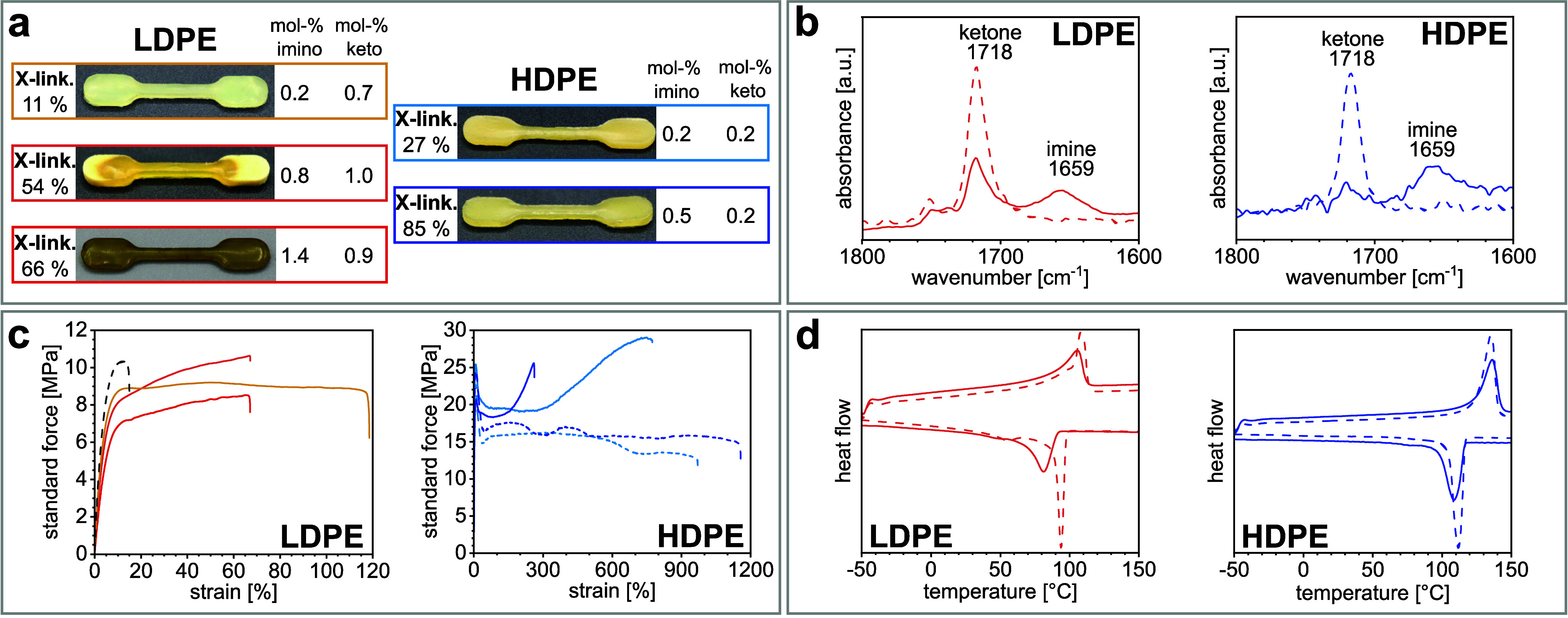
Data on imino-cross-linked PEs. Dashed lines refer to the respective initial keto-PEs for reference. **a:** Photographs of specimens from melt pressing with imino and keto contents determined by IR spectroscopy. Percentages are the gel fractions of each sample. **b:** Exemplary IR spectra of cross-linked materials and initial keto-PE references. **c:** Representative stress–strain curves of cross-linked specimens and keto-PE references. **d:** Comparison of DSC traces of cross-linked materials and keto-PE references (cf. SI for further details and data).

For example, in a keto-LDPE with 4.9 mol % keto content, 57% of functional groups were converted to imines during condensation with 0.5 equiv of 1,12-diaminododecane (cf. Table S4, entry XI2 in SI). The resulting material exhibits a high degree of cross-linking which was reflected in a gel fraction of 97%. However, its melting point and crystallinity were significantly decreased compared to the initial keto-PE (89 vs 105 °C and 21 vs 36%). More PE-like materials were obtained with lower functional group densities: An imino-cross-linked LDPE with keto and imino content of 1.6 and 0.6 mol %, respectively, showed a more LDPE-like melting point and crystallinity of 109 °C (χ = 34%) and still a gel fraction of 70% (cf. Table S4, entry XI1 in SI). The experiments confirmed that functional group densities of 1–2 mol % are most promising to obtain sufficiently cross-linked materials without altering other PE-like properties too much. Tensile specimens of imino-cross-linked LDPE were prepared by compression molding of finely ground mixtures of keto-LDPEs (0.7–4.9 mol % keto) and 0.475 equiv of 1,12-diaminododecane (cross-linking by Method 1, cf. Figure 3, Tables 1 and S5, cf. SI for preparative details and analysis). Typically, 40–60% of keto groups were converted to imines (that is 0.4–1.5 mol % imino content) during this procedure, and no significant amounts of amine were present after the condensation. The imino-cross-linked PEs exhibit melting points of 96–111 °C with a degree of crystallinity of 24–38%. Depending on the cross-link density, gel fractions are in the range of 28–86%. Thermogravimetric analysis showed no significant mass loss in imino-cross-linked PEs up to 300 °C (cf. Figures S42 and S43).

**Table 1:** Summarized Data of Imino-Cross-Linked PEs from Either Direct Condensation of Keto-PEs with 1,12-Diaminododecane (Method 1, Suitable for LDPEs) or Condensation via a Two-Step Procedure over an Amino-Functionalized PE Intermediate (Method 2, Suitable for LDPEs and HDPEs)

#^a^	initial keto-PE [keto mol %]	cross-linking method	*T*_m_ [°C] (% cryst.)^b^	molar ratio imino:keto^c^	χ(C=O) [mol %]^d^	χ(C=N) [mol %]^d^	gel fraction [wt %]^e^
XIL1	0.7	1	111 (38)	40:60	0.6	0.4	28
XIL2	1.2	1	111 (35)	46:54	0.7	0.6	35
XIL3	2.2	1	106 (32)	60:40	0.9	1.4	66
XIL4	4.9	1	96 (24)	39:61	2.3	1.5	86
XIL5	0.7	2	112 (39)	17:83	0.8	0.2	39
XIL6	1.2	2	111 (37)	22:78	0.7	0.2	11
XIL7	2.2	2	105 (31)	44:56	1.0	0.8	54
XIH1	0.4	2	136 (61)	57:43	0.2	0.2	27
XIH2	0.6	2	136 (53)	74:26	0.2	0.5	85

a Condensation conditions: 160 °C, in melt press, first under 1 atm of N_2_ and then gradually reduced pressure down to 10^–2^ mbar.

b Peak melting point and degree of crystallinity determined by DSC, 2nd heating cycle (10 K × min^–1^).

c Determined by band deconvolution of ATR-IR spectra considering the different molar absorption coefficients of imino and keto groups (cf. Figure S18 in SI).

d Keto and imino content with respect to ethylene repeat units of the polymer. Determined by ATR-IR spectra acquired on newly cut cross sectional areas of the specimens.

e Determined by weighing the samples before and after extraction with hot toluene (6 h, 110 °C).

A prerequisite for any possible application of these new materials is their resistance to hydrolysis under ambient conditions. We therefore investigated the persistence of imino-cross-linked LDPEs at ambient temperature both under air and in water (cf. Figures S61 and S62 in SI). After three months under air, virtually no change of gel fraction (33 vs 34 wt %) was observed. Additionally, mechanical properties (*E*_t_ = 314 vs 257 MPa, ε_b_ = 46 vs 41%, and σ_max_ = 11.3 vs 11.8 MPa) did not deteriorate. IR spectra remained unaltered which, overall, points toward a negligible degree of hydrolysis of cross-links under these conditions and high stability of the material.

Keto-HDPEs from catalytic nonalternating ethylene-CO copolymerization with mostly isolated in-chain keto groups were synthesized according to a reported procedure (cf. SI for details on synthesis and characterization of initial keto-HDPEs).^35^ Defect-free cross-linked HDPE specimens could only be prepared by a two-step method which comprises melt pressing of powders under high vacuum throughout (cross-linking by Method 2, cf. Figure 3, Tables 1 and S5, cf. SI for characterization data of obtained cross-linked materials). We also prepared specimens of imino-cross-linked LDPEs by this strategy. Resulting imino-cross-linked HDPE specimens contained 0.2 and 0.5 mol % imino groups, meaning that 57 and 74% of initial keto groups were converted, respectively. The gel fraction of the samples was 27 and 85%, and HDPE-like thermal properties were largely retained (cf. Figure 3, Table 1, and SI for further characterization data).

Mechanical properties of imino-cross-linked PEs and initial keto-PEs are inherently different, and the degree of cross-linking is crucial (cf. Figure 3 and SI for further data). Reference keto-LDPEs have rather low molecular weights, so that their elongation at break is only ∼10% (LDPE-like Young’s modulus of *E*_t_ = 190–240 MPa). In contrast, keto-HDPEs showed excellent mechanical properties (ε_b_ = 830–1100%, *E*_t_ = 820–970 MPa, σ_λ_ = 23 MPa). In cross-linked samples, two effects are operant: 1) The higher the cross-link density the lower the degree of crystallinity, meaning the softer the materials. Therefore, Young’s modulus decreases to 100–200 MPa for imino-cross-linked LDPEs and to 800 MPa for cross-linked HDPEs. 2) On the other hand, very low cross-linking densities (imino content ≤0.5 mol %) negligibly disturb crystallinity but broaden the molecular weight distribution by adding high-molecular-weight fractions to the sample. Those increase both Young’s modulus (LDPE: *E*_t_ = 250–270 MPa, HDPE: *E*_t_ = 1070 MPa) and tensile strength (LDPE: σ_max_ = 11 MPa, HDPE: σ_max_ = 27 MPa). For LDPEs, effect 2) is especially pronounced because the molecular weight of initial keto-PEs is low. Therefore, high molecular weight fractions introduced by cross-linking greatly enhance the elongation at break (up to ε_b_ = 100%). For HDPEs, the qualitative shape of the stress–strain curve with increasing cross-link density changes as observed for HDPEs with increasing weight-average molecular weight *M*_W_.^44,45^ That is, the higher the cross-link density, the lower the yield stress and the following yield drop and the more pronounced the strain hardening during plastic flow. While non-cross-linked keto-PEs, like commercial HDPE, deform inhomogeneously with neck formation, an imino-cross-linked HDPE with 0.5 mol % imino content deforms homogeneously without necking. Similar observations were made for blends of ultrahigh-molecular-weight PE (UHMWPE) and HDPE when increasing the portion of the former.^46^ On a molecular level, these effects are attributed to a reduced chain mobility by both cross-links and entanglement formation in combination with a decreased crystallinity.

## Chemical Recycling by Acidic Hydrolysis

**Figure 4:**
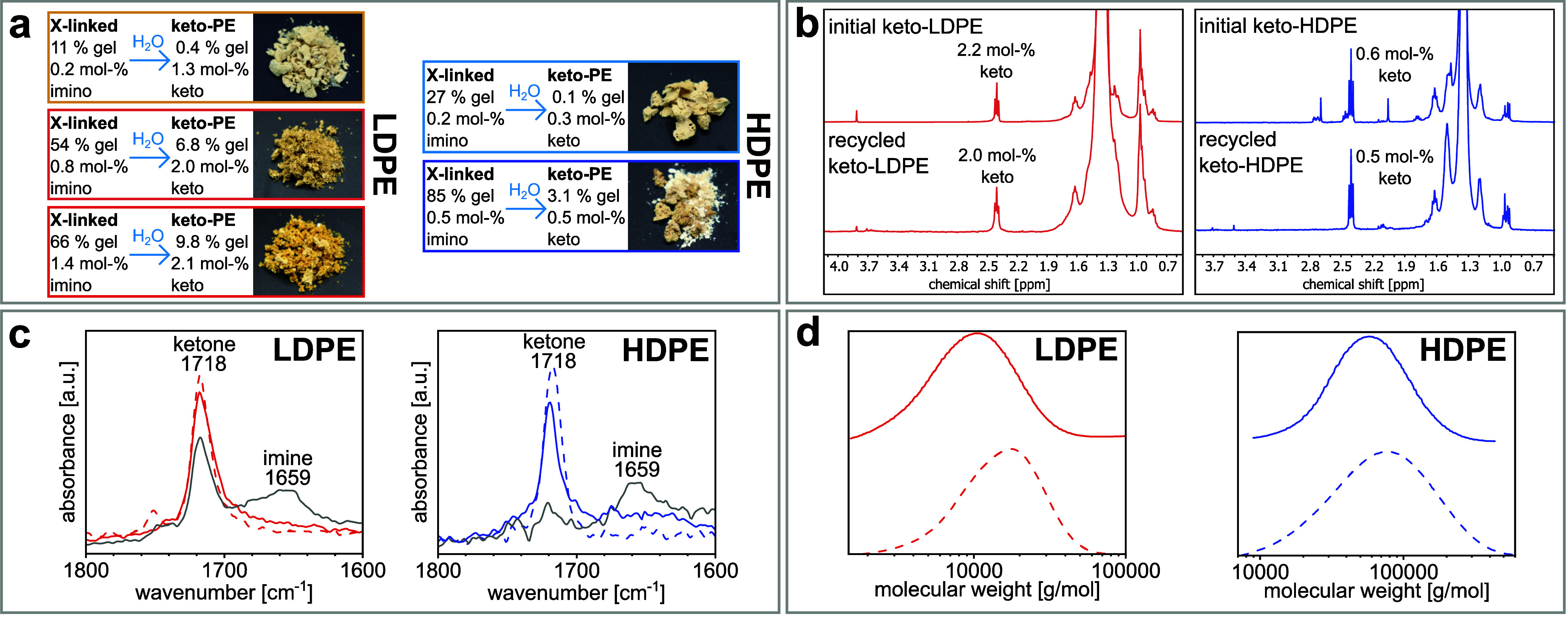
Data on recycled keto-PEs from hydrolysis of imino-cross-linked PEs. Dashed lines refer to the respective initial keto-PEs for reference. **a:** Photographs of recycled materials. Imino contents are determined by IR and keto contents by ^1^H NMR spectroscopy. Percentages are gel fractions of each sample before and after hydrolysis. **b:** Exemplary ^1^H NMR spectra of recycled and initial keto-PEs (373 K, C_2_D_2_Cl_4_). **c:** Representative IR spectra of recycled and initial keto-PEs. Gray spectra are from the respective cross-linked materials before hydrolysis. **d:** Comparative molecular weight distributions of recycled and initial keto-PEs as determined by GPC (cf. SI for further details and data).

Powdered imino-cross-linked PEs were hydrolyzed in an autoclave at 140 °C to yield the initial keto-PEs with good yields of 92–97% (cf. Figure 4 and Table S8 in SI). *p*-Toluenesulfonic acid was used as acid and 1,4-dioxane as solvent for LDPE-based polymers and toluene for HDPE-based polymers, respectively. The keto content of the recycled materials was virtually identical to that of the initial keto-PEs in all experiments, and imines were hydrolyzed quantitatively according to IR (Figure 4). However, 1,4-diketone motifs could not be detected by NMR in the soluble fractions of the recycled materials. This suggests that nonsoluble fractions are rich in nonhydrolyzable 2,5-disubstituted pyrroles. For cross-linked PEs with low to medium cross-link density (0.2–0.5 mol % imino content), the gel fraction of the material after hydrolysis was low (≤3%, cf. Table S8 in SI). In contrast, cross-linked PEs with 0.8–1.4 mol % imino content, that is, higher concentration of pyrrole motifs, had remaining gel fractions of 6–10% after hydrolysis. Molecular weight distributions of all recycled materials contain a small amount of high-molecular-weight fraction (*M*_p_ > 10^6^ g × mol^–1^) which indicates that also in the soluble part of recycled keto-PEs traces of nonhydrolyzable cross-links are present (cf. Figure S59).

In summary, we demonstrated that the newly found class of keto-polyethylenes, both keto-LDPE and keto-HDPE, can be efficiently cross-linked in the melt by imine formation with an appropriate diamine to yield recyclable mimics of cross-linked polyethylene. The materials exhibit high gel contents and tensile properties dominated by the cross-linked nature. Cross-linked LDPEs exhibit much higher elongations at break compared to reference keto-LDPEs. Cross-linked HDPEs deform uniformly as opposed to distinct necking of linear keto-HDPEs and show a pronounced and fast strain hardening. At the same time, the materials can be recycled to the initial melt-processable keto-PEs by acid-catalyzed hydrolysis at temperatures (ca. 140 °C) above the melting temperature of the crystalline segments of the X-PEs. Low keto contents of ≤1.5 mol % are ideal to retain PE-like thermal properties, achieve sufficient cross-link density, and maintain recyclability. The materials are stable in ambient air, which is a prerequisite for their application. This is likely a result of the polyethylene’s hydrophobic nature. Our findings provide a feasible approach for endowing traditionally nonreusable cross-linked polyethylenes with circularity and show how this can be achieved through low densities of functional groups in the polyethylene chains.

## Supporting Information Available

Materials and methods, experimental procedures, experimental and characterization data, and additional references (PDF)

## ABBREVIATIONS

DSC: differential scanning calorimetry
GPC: gel permeation chromatography
HDPE: high-density polyethylene
LDPE: low-density polyethylene
NMR: nuclear magnetic resonance
PE: polyethylene
TGA: thermogravimetric analysis
UHMWPE: ultrahigh-molecular-weight polyethylene
