# Supplementary material for: Circular Cross-Linked Polyethylene Enabled by In-Chain Ketones
Source: ACS Macro Lett. 2024 Nov 15;13(12):1655–61. doi: 10.1021/acsmacrolett.4c00660 (PMC11656715; doi:10.1021/acsmacrolett.4c00660)
Supplement: Supplementary file 1 — mz4c00660_si_001.pdf [file mz4c00660_si_001.pdf]

Supporting Information

Circular Crosslinked Polyethylene Enabled by In-Chain Ketones

Tobias O. Morgen and Stefan Mecking

Chair of Chemical Materials Science, Department of Chemistry, University of Konstanz, 78457 Konstanz, Germany.

Contents

1. Materials and Methods 2
1.1 General considerations 2
1.1.1 Solvents, reagents and materials 2
1.1.2 Analytical methods and general techniques 2
1.2 Experimental procedures 5
1.2.1 Synthesis of low-molecular weight reference imine 5
1.2.2 Condensation of keto-LDPEs with 1-aminotetradecane 5
1.2.3 Crosslinking of keto-LDPEs with diamine on small scale 6
1.2.4 Crosslinking in molds to prepare tensile specimens 6
1.2.5 Determination of gel fractions 8
1.2.6 Hydrolysis procedure 8
2. Experimental and characterization data 10
2.1 Keto-LDPE from free-radical ethylene/CO copolymerization 10
2.2 Keto-HDPE from catalytic ethylene/CO copolymerization 14
2.3 Data on low-molecular weight reference imine 19
2.4 Data on condensation products with monoamine 27
2.5 Crosslinking with diamine on small scale 33
2.6 Crosslinking in molds to prepare tensile specimens 38
2.6.1 Method 1 41
2.6.2 Method 2 45
2.7 Hydrolysis 52
2.8 Stability under Ambient Conditions 60
2.8.1 Stability under Air 60
2.8.2 Stability in water 61
3. References 62

1. Materials and Methods

1.1 General considerations

Non-crosslinked imino-functionalized polyethylenes (PEs) with high imino-content that are non-crystalline, oily substances at room temperature (r.t.) were stored in a glovebox and handled preferably under inert gas to prevent gradual hydrolysis by atmospheric moisture (observed within hours by ATR-IR). Dried, deoxygenated solvents were used in contact with these compounds.

In contrast, semicrystalline non-crosslinked imino-functionalized PEs and all crosslinked samples were considerably more stable when stored under air. However, their stability also depends on their macroscopic morphology, namely powder vs. bulk sample. The former, depending on the degree of crosslinking and crystallinity, were also found to hydrolyze significantly within days, so that they also had to be stored under inert gas. According to ATR-IR, imine hydrolysis on the surface of bulk samples by atmospheric moisture was fast (observed within minutes), whereas groups inside the sample remained intact over a long period.

1.1.1 Solvents, reagents and materials

If necessary, solvents were dried and degassed using standard laboratory techniques. Toluene (> 99 % from Carl Roth) was dried and deoxygenated by passing it through columns filled with BASF PuriStar R3-11 catalyst and molecular sieves (3 Å). 1,4-Dioxane (> 99.5 % from Carl Roth) was deoxygenated by freeze-pump-thaw degassing and water by distillation under a constant nitrogen stream. All NMR solvents (purchased from Deutero) were dried over molecular sieves for at least 1 week. 1-Aminotetradecane (> 95 %) was purchased from abcr and used as received. 1,12-Diaminododecane (> 98 %) from TCI chemicals was also used without further purification. Toluene sulfonic acid monohydrate ($TsOH \cdot H_2O$) with a purity of > 98.5 % from Merck was used as received.

Keto-functionalized low-density polyethylenes (keto-LDPEs) were prepared by free-radical copolymerization of ethylene and carbon monoxide initiated by di-*tert*-butyl peroxide in dimethyl carbonate. The respective setup, procedure and characterization were described elsewhere in more detail.[1] Keto-functionalized high-density polyethylenes (keto-HDPEs) were prepared according to literature by catalytic non-alternating copolymerization of ethylene and CO catalyzed by a phosphinophenolato Ni-catalyst in toluene.[2] The respective Ni-catalyst precursor with C_6F_5 and 2,6-dimethoxy-1,1'-biphenyl substitution was also prepared according to published work.[3]

1.1.2 Analytical methods and general techniques

Nuclear magnetic resonance (NMR) spectra were recorded on a Bruker Avance III HD 400 (^1H: 400 MHz, ^{13}C: 101 MHz). ^1H chemical shifts were referenced to the residual solvent proton signals ($CDCl_3$: 7.26 ppm, $C_2D_2Cl_4$: 6.00 ppm). ^{13}C chemical shifts were referenced to the carbon signal of the deuterated solvent ($CDCl_3$: 77.16 ppm, $C_2D_2Cl_4$: 73.78 ppm). NMR spectra of all keto-polyethylenes were acquired in 1,1,2,2-tetrachloroethane-d_2 at 100 °C. Functional group densities in mol-% are given with respect to the total number of ethylene and carbon monoxide repeat units in the polymer. Thus, all values for imino-polyethylenes are corrected by the number of additional methylene (and methyl) groups introduced into the material by the aliphatic amines (*cf.* Equations S1 and S2).

Gel permeation chromatography (GPC) was performed on a PolymerChar GPC-IR instrument equipped with an integrated four-capillary viscometer and an IR4 dual wavelength infrared detector (selective for methylene and methyl groups) on PSS Polefin Linear XL columns (3 × 30 cm) and with an additional guard column at 160 °C in 1,2-dichlorobenzene. Universal or linear calibration using narrow polystyrene or polyethylene standards, respectively, was employed. The former was used for keto-HDPE, the latter for keto-LDPE samples. Both were measured with a flow rate of 1.0 mL×min^{-1}. The raw data was evaluated with PSS WinGPC UniChrom software.

Differential scanning calorimetry (DSC) was performed on a Netzsch DSC 204 F1 with a bicyclic temperature program and heating/cooling rates of 10 K×min^{-1} on bulk polymer samples. For the measurements, the polymers were weighed into sealed 40 µL aluminum pans.

ATR-IR spectra of polymers were acquired on a Perkin Elmer Spectrum 100 instrument. Spectra of specimens were measured on newly cut cross sectional areas to probe the properties of the bulk material. For calculation of the keto content of initial keto-PEs, the ratio of the integral of the C=O signal (integration area: band between 1709-1715 cm^{-1} ± 65 cm^{-1}) to the integral of the C-H signal of the PE at ~2915 cm^{-1} (integration area: 2740-3030 cm^{-1}) was calculated and referenced with linear polyketone samples with known C=O content synthesized via ADMET copolymerization and subsequent hydrogenation (*cf.* Figure S1).[4]

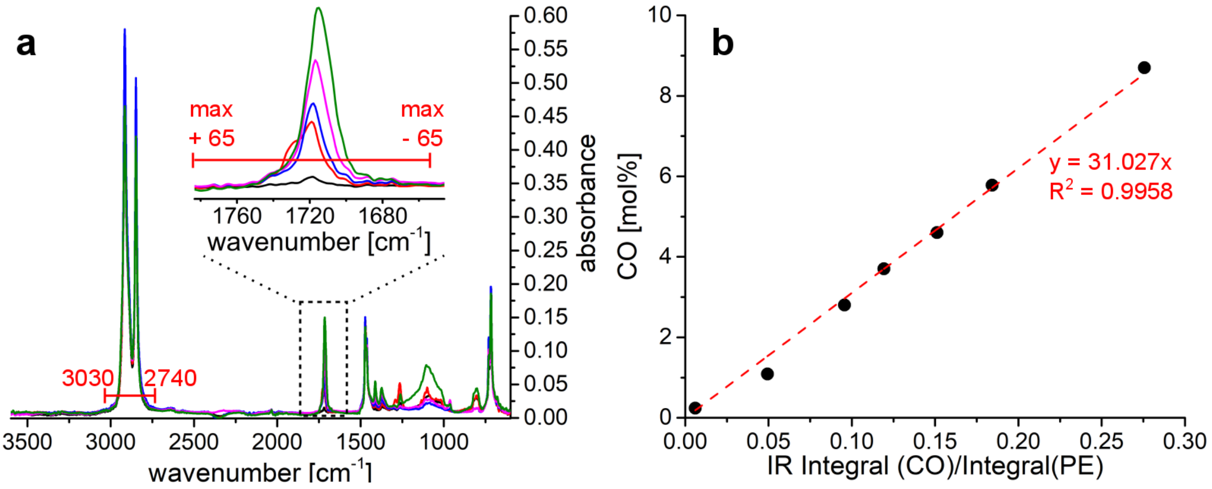

Figure S1. Referencing of IR spectra. **a**: Polyketones with known carbonyl contents were analyzed by ATR-IR. The samples were synthesized via ADMET copolymerization of docosa-1,21-dien-11-one and undeca-1,10-diene followed by hydrogenation.[4] The limits of integration for the determination of signal intensity ratios for carbonyl stretching vibrations (spectral position with max. absorbance ± 65 cm^{-1}) vs. the polyethylene C-H vibration (2740-3030 cm^{-1}) are depicted in red. **b**: The integral intensity ratio is directly proportional to the concentration of C=O groups in the polymer χ. That is, $\chi \approx n_{CO}/n_{C_2H_4}$ which is a valid approximation if $n_{C_2H_4} >> n_{CO}$.[1]

Due to overlapping IR signals, calculation of the keto- and imino-content in imino-functionalized PEs was implemented by peak deconvolution with Lorentzian functions in *Origin*. Therefore, 4 to 5 Lorentzian functions with different spectral positions were used to fit the IR spectrum in the range of 1550 – 1800 cm^{-1}: Isolated keto groups in a PE matrix evoke a band at 1718 cm^{-1} (1) which is gradually shifted down to ~1700 cm^{-1} (2) the higher the concentration of keto- and imino-groups in spatial proximity.[1,5] On the other hand, isolated imino-groups in a hydrocarbon medium evoke a band at 1659 cm^{-1} (3). The band of imino

groups also gradually shifts down to 1640-1650 cm^{-1} (4) when other keto or imino groups are in close proximity. Keto-LDPEs from free-radical ethylene/CO copolymerization in dimethyl carbonate also contain small amounts of methyl polymeryl carbonate end groups from radical chain transfer to the solvent.[1,6] Therefore, these groups were also present in resulting imino-functionalized LDPEs and called for consideration of a fifth band at 1751 cm^{-1} (5). After deconvolution, the integrals of bands related to keto groups were quantified with respect to the PE C-H vibration (2740-3030 cm^{-1}) according to the calibration line in Figure S1. Band integrals of imino groups were first multiplied by a factor of 1.172 (correction of the different molar absorption coefficients of keto and imino groups, *cf.* Section 2.3 for further details) and then quantified as keto groups. The so obtained keto and imino contents χ_{keto} and χ_{imino} are relating to the methylene (and methyl) groups of the whole sample, meaning those of PE and the alkyl chain of the amine/diamine. The values were then corrected by the dilution effect of the latter to obtain functional group densities $\chi_{keto,corr}$ and $\chi_{imino,corr}$ with respect to the polymer repeat units. The following equations were used for correction in the case of samples with 1-aminotetradecane:

(S1) $$\chi_{keto,corr} = \frac{100 \cdot \chi_{keto}}{100 - \chi_{imino} \cdot 7}$$

(S2) $$\chi_{imino,corr} = \frac{100 \cdot \chi_{imino}}{100 - \chi_{imino} \cdot 7}$$

Values of samples with 1,12-diaminododecane were corrected according to:

(S3) $$\chi_{keto,corr} = \frac{100 \cdot \chi_{keto}}{100 - \chi_{imino} \cdot 3}$$

(S4) $$\chi_{imino,corr} = \frac{100 \cdot \chi_{imino}}{100 - \chi_{imino} \cdot 3}$$

Note that imino functional group densities in samples containing significant amounts of unreacted amino groups after the condensation reaction were not directly accessible by ATR-IR, because one amine band strongly overlaps with the band of the imine (*cf.* Figures S31 and S32). However, the keto content in these samples was estimated as described in Figure S1.

Cryo milling was performed in a CryoMill by Retsch GmbH connected to a liquid nitrogen dewar. The samples were auto-precooled and then milled at a shaking rate of 30 Hz for 1 min followed by 30 s of cooling at 5 Hz. This procedure was repeated over six cycles in total.

Reference test specimens (ISO 527-2, type 5B) of keto-LDPE/HDPE for tensile testing were prepared by melt pressing of the materials in a custom-made mold with pistons pressed by two metal screw clamps (*cf.* Figures S2, S3 and Section 1.2.4). Four specimens can be prepared at the same time. 4× ca. 200 mg of the respective powdery sample were weighted into the cavities of the mold. Thin PTFE sheets above and below the individual samples ensured sufficient sealing to prevent the melt from flowing out. The filled mold with the tightened screw clamps was placed in a stainless steel vessel of 1.4 L inner volume equipped with a ball valve (*cf.* Figures S2 and S3). The latter was connected to a Schlenk line. After three cycles of applying vacuum and purging with nitrogen, the vessel was kept under vacuum (2×10^{-2} mbar) and placed

in a pre-heated aluminum heating block (typically 140 – 160 °C). After a certain period of time, the heater was turned off and the melts were allowed to slowly cool to r.t. under vacuum over night. The vessel was purged with nitrogen, opened and the specimens were pressed out after removing the bottom side of the mold. Specimens of imino-crosslinked PEs were prepared in the same setup but with a slightly modified process. The crosslinking condensations were carried out directly in the mold (*cf.* Section 1.2.4 for further details).

Tensile tests were performed on a Zwick Z005/1446 Retroline tC II instrument at a crosshead speed of 2 mm×min^{-1}. The tensile modulus was determined with a speed of 0.5 mm×min^{-1}. The Zwick Roell testXpert III software was used for data evaluation.

Thermogravimetric analysis (TGA) was performed on an STA 429 instrument by Netzsch. 10 mg of the respective sample were heated up to 670 °C under nitrogen and then to 920 °C under oxygen in order to burn residual carbon compounds.

1.2 Experimental procedures

1.2.1 Synthesis of low-molecular weight reference imine

1 mmol of 1,12-diaminododecane (200 mg, 1 equiv.) and 2 mmol of 9-heptadecanone (509 mg, 2 equiv.) were placed under nitrogen in an 8 mL vial closed with a silicone septum. The vessel was connected to both a Schlenk line and a membrane pump via cannula. The mixture was stirred and heated to 120 °C for 2 h. Subsequently, the internal pressure was gradually decreased to 10 mbar over the course of 3 h and then kept under these conditions for an additional hour. The mixture was allowed to cool down to r.t. under nitrogen and was used without any further purification. The product was obtained as a colorless oily liquid (610 mg) containing 83 % of imino, 8 % of amino and 9 % of keto groups (conversion of ketone to imine of 90 %).

^{1}H NMR (400 MHz, $CDCl_3$, 300 K): δ 3.25 (t, J = 7.4 Hz, 2H, C=N-CH_2), 2.21 – 2.11 (m, 4H, N=C-CH_2), 1.57 (v. quintet, J = 7.1 Hz, 2H, C=N-CH_2-CH_2), 1.48 (v. quintet, J = 7.5 Hz, 2H, N=C-CH_2-CH_2), 1.41 (v. quintet, J = 7.4 Hz, 2H, N=C-CH_2-CH_2), 1.26 (s, 28H, CH_2), 0.86 (m, 6H, CH_3).

^{13}C {^{1}H} NMR (101 MHz, $CDCl_3$, 300 K): δ 173.27 (*C*=N), 50.94 (N-*C*H_2), 40.58 (N=C-*C*H_2), 31.96 (*C*H_2-CH_2-CH_3), 31.38 (N-CH_2-*C*H_2), 30.93 (N=C-*C*H_2), 29.81 (*C*H_2), 29.73 (*C*H_2), 29.62 (*C*H_2), 29.47 (*C*H_2), 29.39 (*C*H_2), 29.26 (*C*H_2), 27.75 (N-CH_2-CH_2-*C*H_2), 27.19 (N=C-CH_2-*C*H_2), 26.63 (N=C-CH_2-*C*H_2), 22.76 (*C*H_2-CH_3), 14.21 (*C*H_3).

1.2.2 Condensation of keto-LDPEs with 1-aminotetradecane

100 mg of the respective keto-LDPE were placed in a glass vial together with a stir bar. 1-Aminotetradecane (4 equiv. with respect to keto groups in the polymer) was added and the vial was placed in a custom-made stainless steel vessel (1.4 L inner volume) connected to a Schlenk line and a pump. The vessel was evacuated and purged with nitrogen three times and was placed in an aluminum block preheated to 160 °C. While stirring with 50 rpm, the sample was kept at that temperature under N_2 for 1 h. Subsequently, the internal pressure was reduced to 400 mbar for another 6 h. The pressure was then reduced to 2×10^{-2} mbar for 2 h to remove the excess of 1-aminotetradecane from the polymer melt. Afterwards, the vessel was purged with nitrogen and cooled to room temperature. The obtained imino-functionalized LDPEs were analyzed

without further purification and were stored in a glovebox to prevent gradual hydrolysis by atmospheric moisture.

1.2.3 Crosslinking of keto-LDPEs with diamine on small scale

Preliminary crosslinking experiments were carried out by a procedure analogous to that described in Section 1.2.2 with keto-LDPEs and 1,12-diaminododecane. 100 mg of powdery keto-LDPE were mixed with the respective amount of diamine (1.0 or 2.0 equiv. with respect to keto groups in the polymer) in a vial. The reaction was then carried out according to the protocol described above. The samples were slight stirred (50 rpm) only during the first hour of the reaction before pressure reduction. The obtained imino-crosslinked LDPEs were analyzed without further purification and were stored in a glovebox.

1.2.4 Crosslinking in molds to prepare tensile specimens

Test specimens of imino-crosslinked PEs were prepared by melt pressing in the same setup as described for non-crosslinked reference keto-PEs (*cf.* Section 1.1.2) but with a slightly modified process. The crosslinking reaction was carried out directly in the mold during a shaping and a subsequent hardening step. Specimens of imino-crosslinked LDPEs were prepared by both Method 1 and 2, whereas the high melt viscosity of keto- and imino-functionalized HDPEs impeded specimen preparation by Method 1 in this case. When keto-HDPE powders were heated under N_2 in the mold (regardless of whether with or without diamine), gas inclusions were trapped inside the highly viscous liquid which apparently could not be removed by applying vacuum at a later stage. It turned out to be crucial to melt HDPE powders directly under high vacuum (10^{-2} mbar, 160 °C) to obtain defect-free specimens. This however, would call for a diamine crosslinker that is non-volatile under these conditions, which, to the best of our knowledge, is not commercially available. To circumvent this problem, we instead pursued a two-step method retaining 1,12-diaminododecane (Method 2).

<u>Method 1:</u>

1 g of the respective keto-LDPE and 1,12-diaminododecane (typically 0.95 equiv. of amino groups with respect to keto groups) were finely grinded in a cryomill (*cf.* Section 1.1.2). 4× ca. 200 mg of the powdery mixture were weighted into the cavities of the mold for specimen preparation according to ISO 527-2, type 5B (*cf.* Figure S2). Thin PTFE sheets above and below the individual samples ensured sufficient sealing to prevent the melt from flowing out. The upper pistons were introduced and pressed by two metal screw clamps. The filled and pressed mold was then placed in a stainless steel vessel of 1.4 L inner volume connected to a Schlenk line (*cf.* Figure S2). After three cycles of applying vacuum and purging with nitrogen, the vessel was kept under nitrogen and placed in a pre-heated aluminum heating block (160 °C). After 6 h of shaping, the heater was turned off and the vessel was allowed to cool to r.t. It was opened, the screw clamps, the upper pistons and upper PTFE sheets were removed and the open mold was again placed in the vessel. After threefold evacuation and purging with nitrogen, the specimens were further hardened by heating to 160 °C under N_2 for 5 h followed by 2 h at 160 °C under vacuum (10^{-2} mbar). The vessel was then cooled, purged with nitrogen, opened

and the specimens were pressed out after removing the bottom side of the mold.

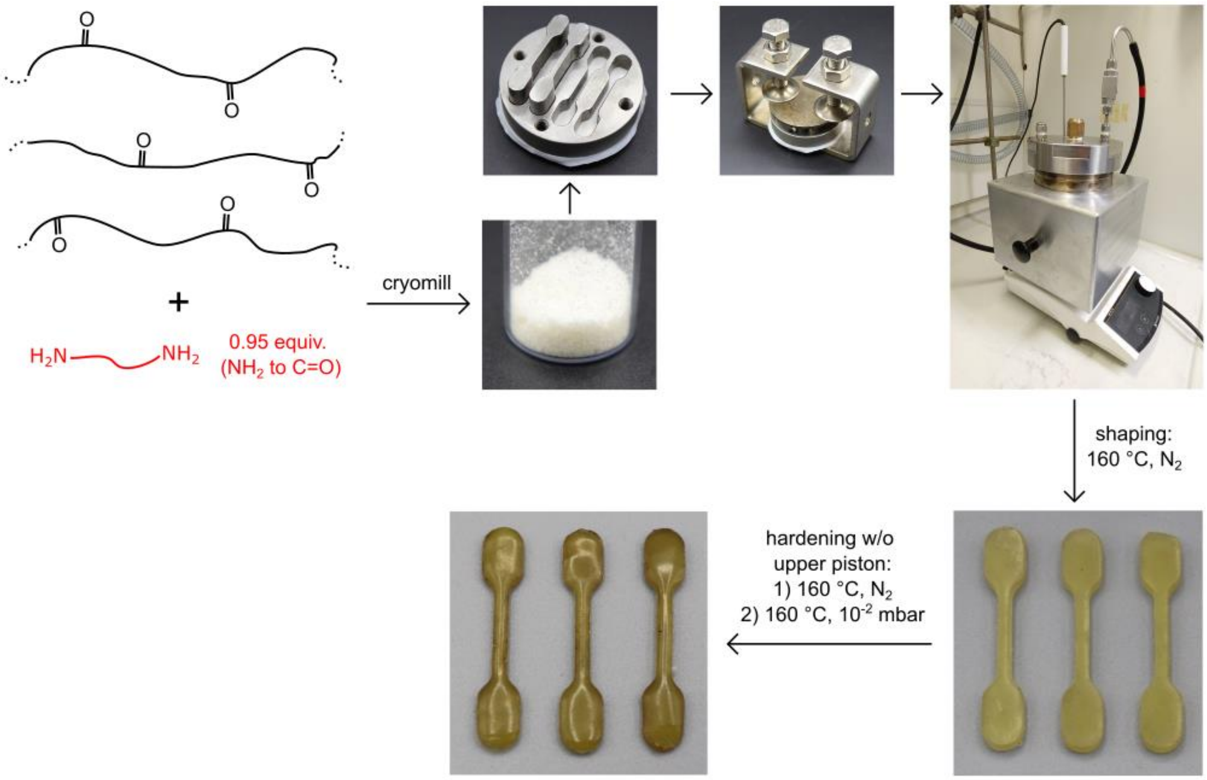

Figure S2. Preparation of tensile test specimens by Method 1: 1) Mixing of keto-PE and diamine by cryomilling. 2) Pressing of the powdery samples in a costum-made mold. 3) Crosslinking of the pressed mixture upon melting during shaping and hardening in a stainless steel vessel. Imino-crosslinked LDPEs were prepared with this method.

Method 2:

In a glass vial, 500 mg of the respective keto-LDPE or keto-HDPE were mixed with an excess of 1,12-diaminododecane (typically 20 – 30 equiv. of amino groups with respect to keto groups). The vial was placed in a Schlenk flask and purged with nitrogen. The amino-functionalized PEs (for analytics see Table S7 and Figures S44 - S46) were synthesized by melting the mixture at 160 °C for 6 h. Excess of diamine was then removed at the same temperature under vacuum (10^{-2} mbar) for 1 h. After cooling, the solidified material was removed and mixed with the respective amount of keto-LDPE/keto-HDPE (0.95 equiv. of amino groups with respect to keto groups). The mixture was grinded in a cryomill (*cf.* Section 1.1.2) and pressed in the mold for test specimen preparation as described (*cf.* Figure S3). In contrast to Method 1, the samples were directly molten under vacuum at 160 °C. After 6 h of shaping, the heater was turned off and the vessel was allowed to cool to r.t. It was opened, the screw clamps, the upper pistons and upper PTFE sheets were removed and the open mold was again placed in the vessel. After threefold evacuation and purging with nitrogen, the specimens were fully hardened for 7 h at

160 °C under vacuum (10^{-2} mbar). The vessel was cooled, purged with nitrogen, opened and the specimens were pressed out.

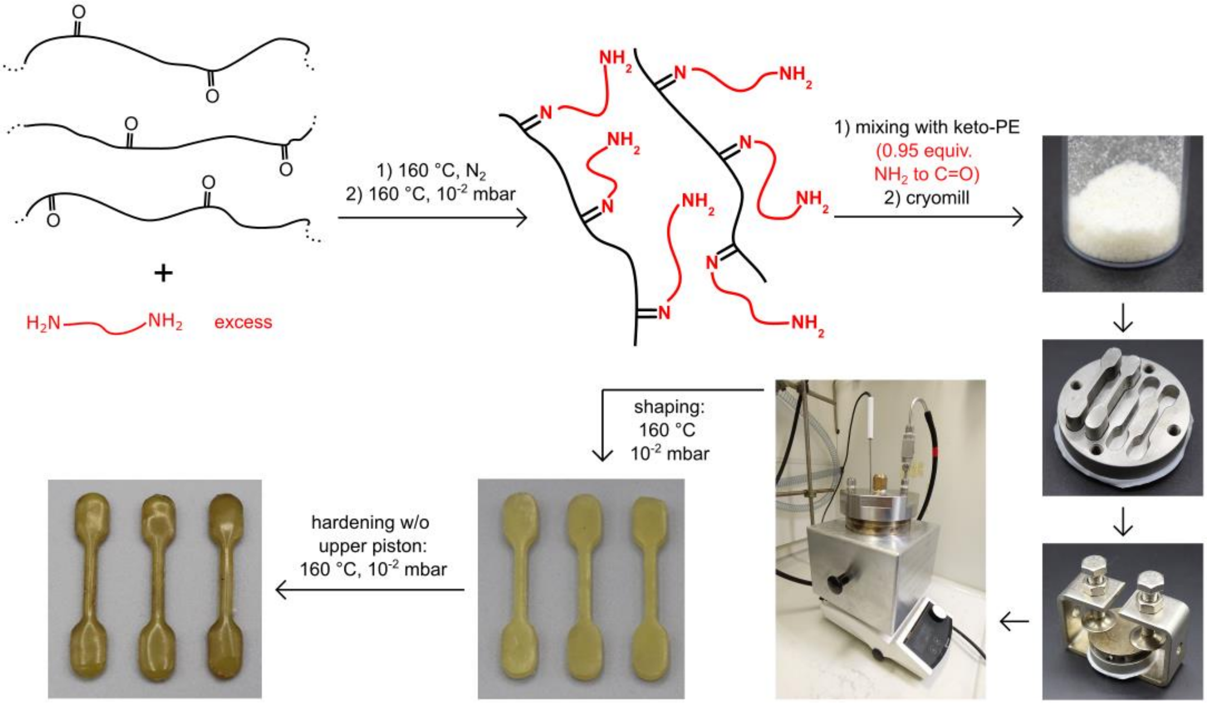

Figure S3. Preparation of tensile test specimens by Method 2: 1) Condensation of keto-PE with an excess of diamine. 2) Mixing of amino-functionalized PE and keto-PE by cryomilling. 3) Pressing of the powdery samples in a mold. 4) Crosslinking of the pressed mixture upon melting during shaping and hardening in a stainless steel vessel. Imino-crosslinked LDPEs and HDPEs were prepared with this method.

1.2.5 Determination of gel fractions

30 mg of imino-crosslinked PE in small pieces with thicknesses of 0.5 – 1 mm were weighted into glass vials. 10 mL of dry and O_2-free toluene was added to each vial in a glovebox and the vials were sealed. The samples were heated to 110 °C and slightly shaken for 6 h followed by rapid filtration through a steel mesh with a mesh size of 74 ± 15 µm. The separated insoluble pieces were then briefly washed with toluene, dried *in vacuo* and weighed.

The procedure was repeated both with non-crosslinked keto-LDPEs and non-crosslinked keto-HDPEs which were all entirely soluble under these conditions, so that no material remained on the mesh after hot filtration (*cf.* Table S4 and S5).

1.2.6 Hydrolysis procedure

Hydrolysis was carried out in a 200 mL stainless steel mechanically stirred pressure reactor (BüchiGlasUster) equipped with a heating jacket, in- and outlet valves, a manometer and a glass insert. The temperature was controlled by a thermostat connected to a thermocouple dipping

into the reaction mixture. CAUTION: High temperatures and high concentrations of *p*-toluenesulfonic acid damage stainless steel! Protection of equipment by glass fittings is highly recommended. With concentrations of ca. 250 mg of *p*-toluenesulfonic acid monohydrate per 100 mL of solvent, we did not observe any signs of corrosion during the experiments described here.

Specimens of imino-crosslinked LDPEs were cut to small pieces and then finely grinded in a cryomill (for details *cf.* Section 1.1.2). 800 mg of the respective powdery sample and 240 mg of *p*-toluenesulfonic acid monohydrate (>2 equiv. with respect to total amount of imino + keto groups in the polymer) were weighed into the glass insert. The latter was then placed inside the reactor followed by cautious evacuation and purging with nitrogen three times. 100 mL of deoxygenated 1,4-dioxane and 1.5 mL of water (>100 equiv. with respect to C=N + C=O in the polymer) were transferred into the vessel by cannula, the reactor was closed and heated to 140 °C while stirring with 500 rpm. After 72 h, stirring was interrupted, the mixture was cooled to 90 °C and carefully added to acidic 750 mL of methanol (750 mL MeOH + 10 mL concentrated HCl). The precipitated polymer was filtered off, washed with methanol and vacuum dried at 50 °C for >24 h.

Imino-crosslinked HDPEs were hydrolyzed in the same setup following a similar procedure but in another reaction medium: 400 mg of the milled polymer sample and 240 mg of *p*-toluenesulfonic acid monohydrate (>15 equiv. with respect to total amount of imino + keto groups in the polymer) were weighed into the glass insert. After degassing as described above, 100 mL of O_2-free toluene and 1.5 mL of water (>1000 equiv. with respect to C=N + C=O in the polymer) were added to the vessel via cannula transfer. The reactor was closed and heated to 140 °C while stirring with 500 rpm. After 72 h, the reaction was cooled and work-up was performed as described above.

2. Experimental and characterization data

2.1 Keto-LDPE from free-radical ethylene/CO copolymerization

Table S1. Summarized polymerization results, molecular weights, thermal properties and microstructures of initial keto-LDPEs used in this work.

#	p_{CO} [%][a]	yield [g]	M_n [10^3 g×mol^{-1}] (M_w/M_n)[b]	T_m [°C] (% cryst.)[c]	χ(CO) [mol-%][d]	br/1000 C atoms[e]	I/D3/D2/D1 [%][f]
L1	0.7	11.23	14.4 (1.5)	109 (44)	0.7 (0.6)	n.d.	1 % D1
L2	1.1	10.60	12.3 (1.7)	109 (37)	1.2 (1.3)	n.d.	2 % D1
L3	1.4	8.42	12.1 (1.4)	108 (33)	2.2 (2.0)	7	4 % D1
L4	2.0	8.36	11.0 (1.4)	105 (36)	4.9 (3.6)	8	75/13/6/6
L5	2.9	7.67	10.0 (1.5)	104 (32)	5.1 (4.5)	8	72/15/6/7
L6	4.9	7.59	9.95 (1.4)	102 (28)	7.6 (5.8)	8	61/17/13/9
L7	7.0	6.00	9.65 (1.4)	97 (25)	12.6 (10.2)	7	46/19/19/16

Reaction conditions: 140 °C, 400 bar max. total pressure, 3 h, 75 mL dimethyl carbonate, 4 mM di-*tert*-butyl peroxide. [a]Initial partial pressure of CO p_{CO} / (p_{CO}+p_E). [b]Determined by GPC in 1,2-dichlorobenzene at 160 °C via linear calibration against PE standards. [c]Peak melting point and degree of crystallinity determined by DSC, 2nd heating cycle (10 K×min^{-1}). [d]Keto content with respect to ethylene repeat units determined by ^{1}H NMR (and by ATR-IR, *cf.* Figure S1). [e]Branches per 1000 carbon atoms determined by ^{13}C NMR. [f]Ratio of isolated keto motifs (I) and double keto motifs separated by either three (D3), two (D2) or one (D1) ethylene repeat unit. Determined by ^{1}H NMR (entries L1-L3) or ^{13}C NMR (entries L4-L6).

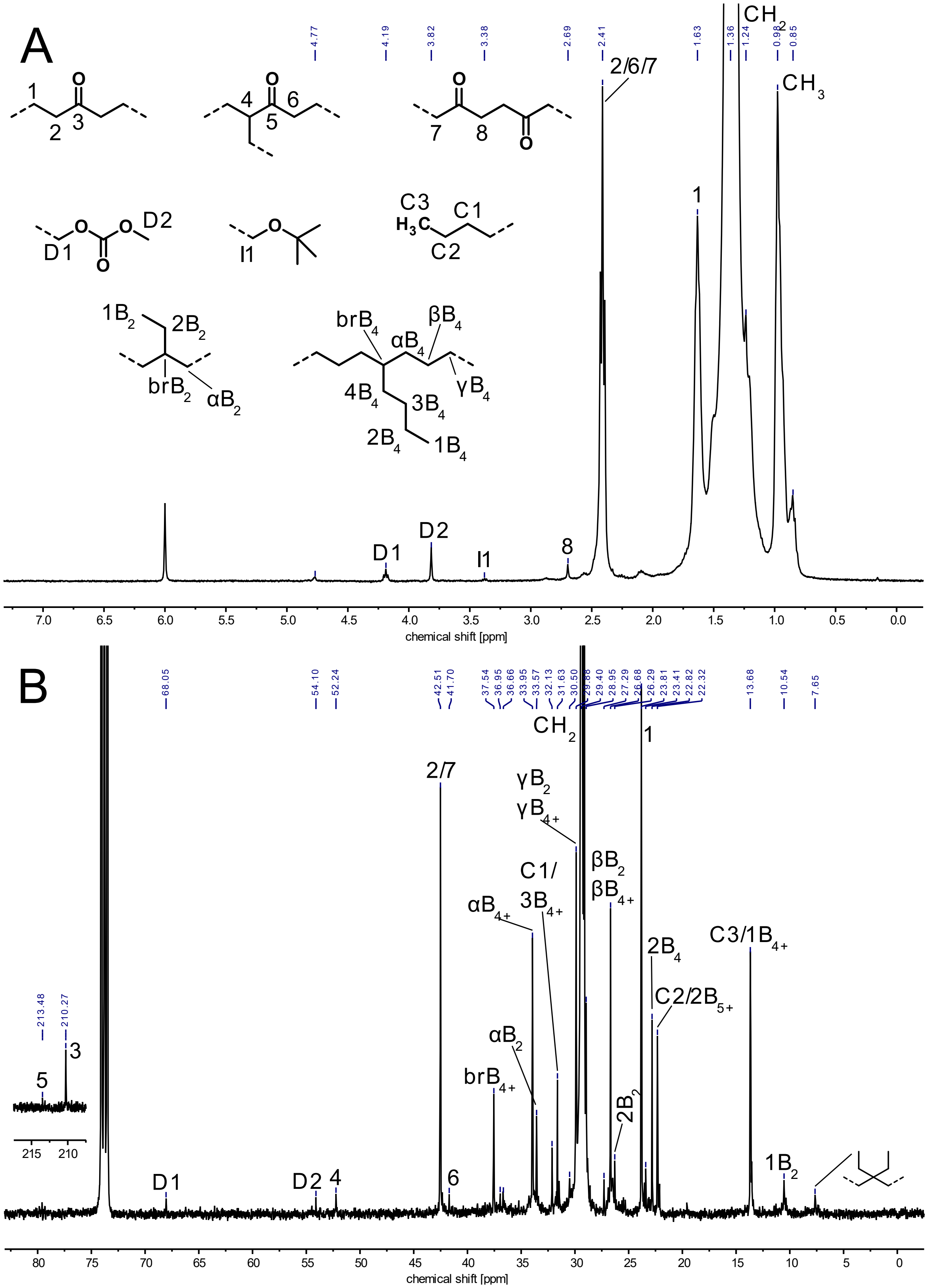

Figure S4. **A**: ^{1}H NMR and **B**: ^{13}C {^{1}H} NMR spectrum ($C_2D_2Cl_4$, 373 K) of a keto-LDPE with 2.2 mol-% keto groups obtained from free-radical copolymerization prior to condensation with amines (Table S1, entry L3).

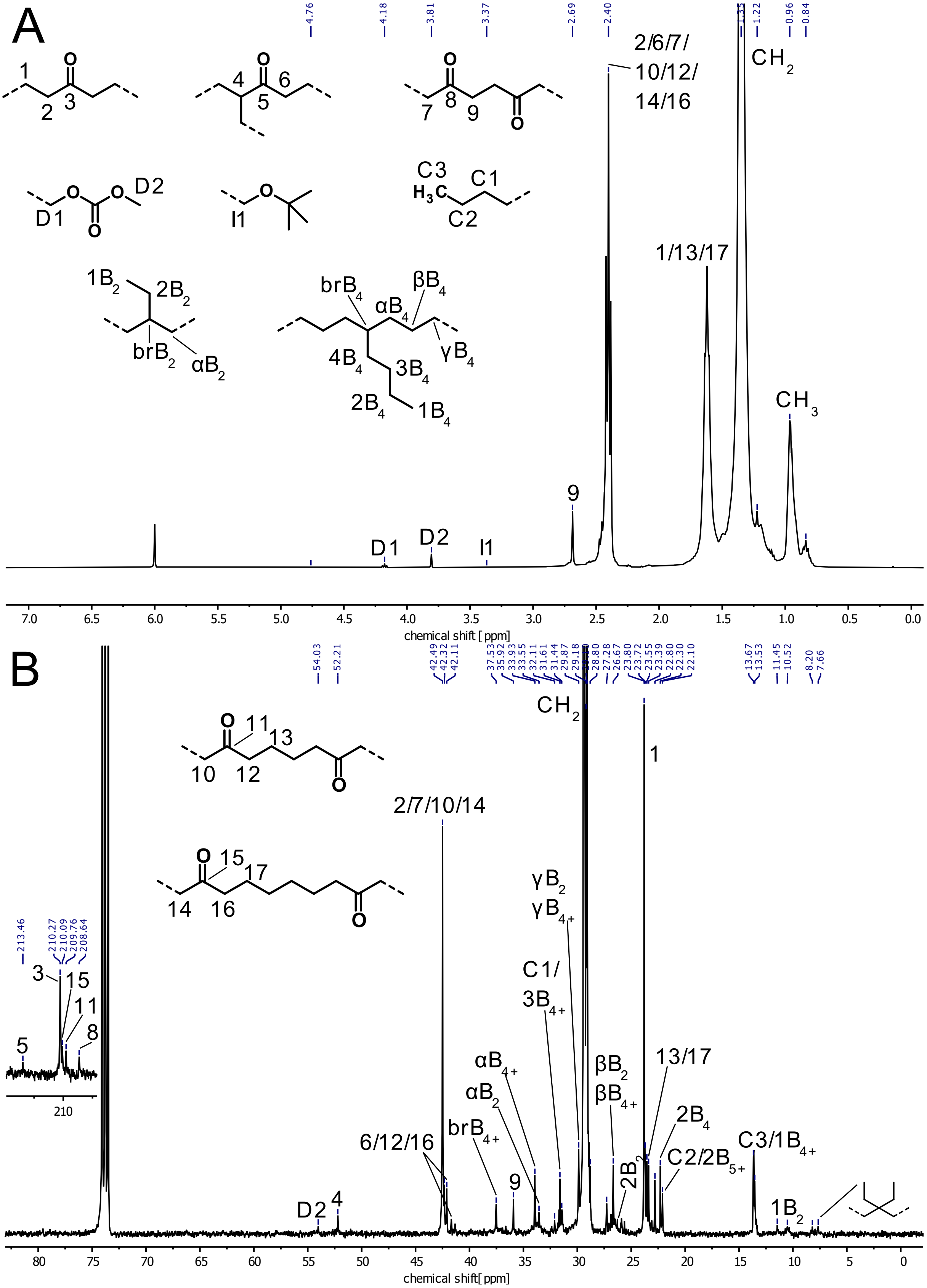

Figure S5. **A:** ^{1}H NMR and **B**: ^{13}C {^{1}H} NMR spectrum ($C_2D_2Cl_4$, 373 K) of a keto-LDPE with 7.6 mol-% keto groups obtained from free-radical copolymerization prior to condensation with amines (Table S1, entry L6).

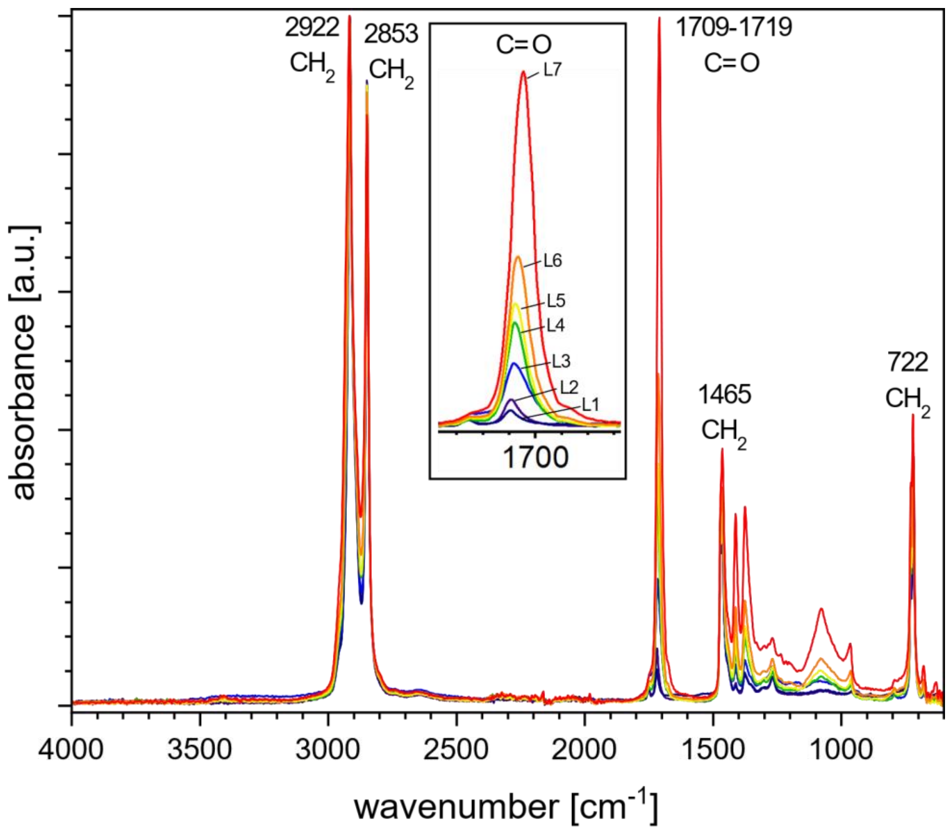

Figure S6. ATR-IR spectra of keto-LDPEs (0.7 – 12.6 mol-% keto) summarized in Table S1. Spectra are normalized to the maximum at 2922 cm^{-1}.

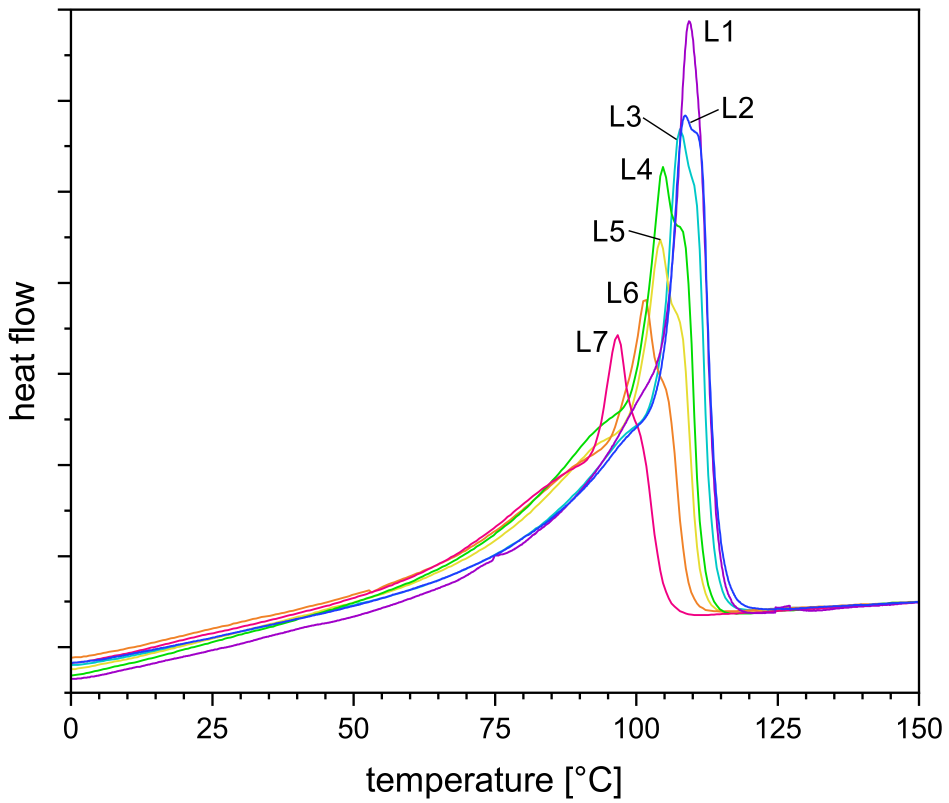

Figure S7. 2nd DSC heating curves of keto-LDPEs (0.7 – 12.6 mol-% keto) of Table S1.

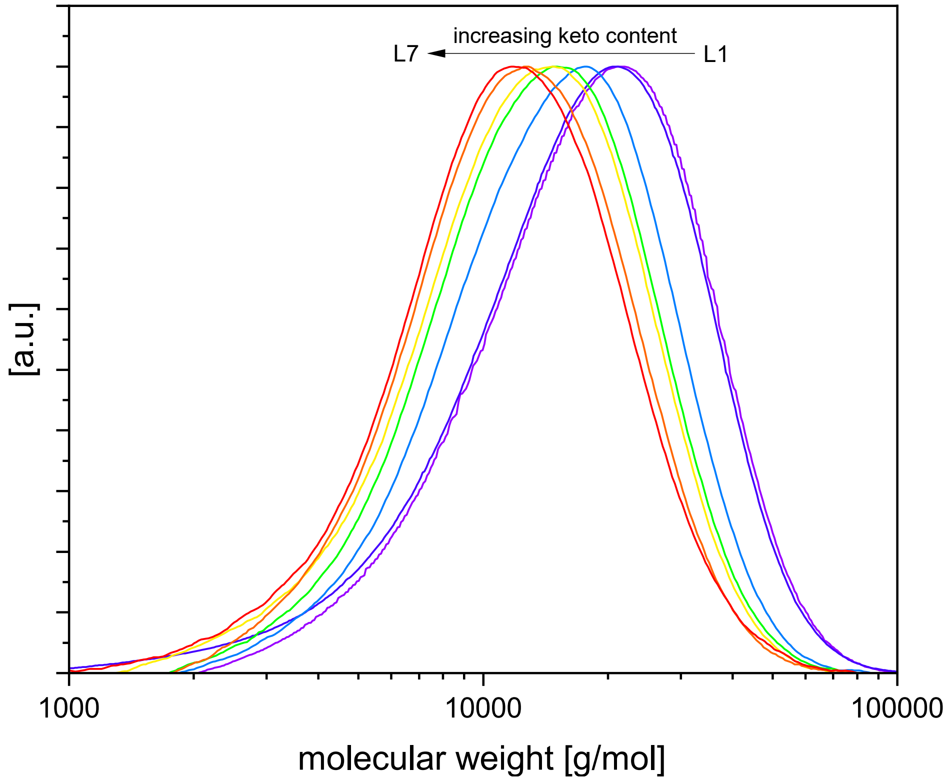

Figure S8. GPC molecular weight distributions of keto-LDPEs (0.7 – 12.6 mol-% keto) of Table S1.

2.2 Keto-HDPE from catalytic ethylene/CO copolymerization

Table S2. Summarized polymerization results, molecular weights, thermal properties and microstructures of initial keto-HDPEs.

#	CO in feed [mol-%][a]	yield [g]	M_n [10^3 g×mol^{-1}] (M_w/M_n)[b]	T_m [°C] (% cryst.)[c]	χ(CO) [mol-%][d]	br/1000 C atoms[e]	I/D1/A [%][f]
H1	0.2	2.02	53.9 (1.7)	134 (68)	0.4 (0.4)	0.5	69/17/14
H2	0.3	1.90	51.9 (1.8)	135 (67)	0.6 (0.6)	0.6	68/18/14

Reaction conditions: 10 µmol Ni precatalyst, 100 °C, 5 bar total pressure, 100 mL toluene, 1 h. [a]Carbon monoxide content in the feed gas. [b]Determined by GPC in 1,2-dichlorobenzene at 160 °C via universal calibration against polystyrene standards. [c]Melting point and degree of crystallinity determined by DSC, 2nd heating cycle (10 K×min^{-1}). [d]Keto content with respect to ethylene repeat units determined by ^{1}H NMR (and by ATR-IR, *cf.* Figure S1). [e]Branches per 1000 carbon atoms determined by ^{1}H NMR. [f]Ratio of isolated keto motifs (I), double keto motifs separated by one ethylene repeat unit (D1) and alternating segments (A). Determined by ^{1}H NMR.

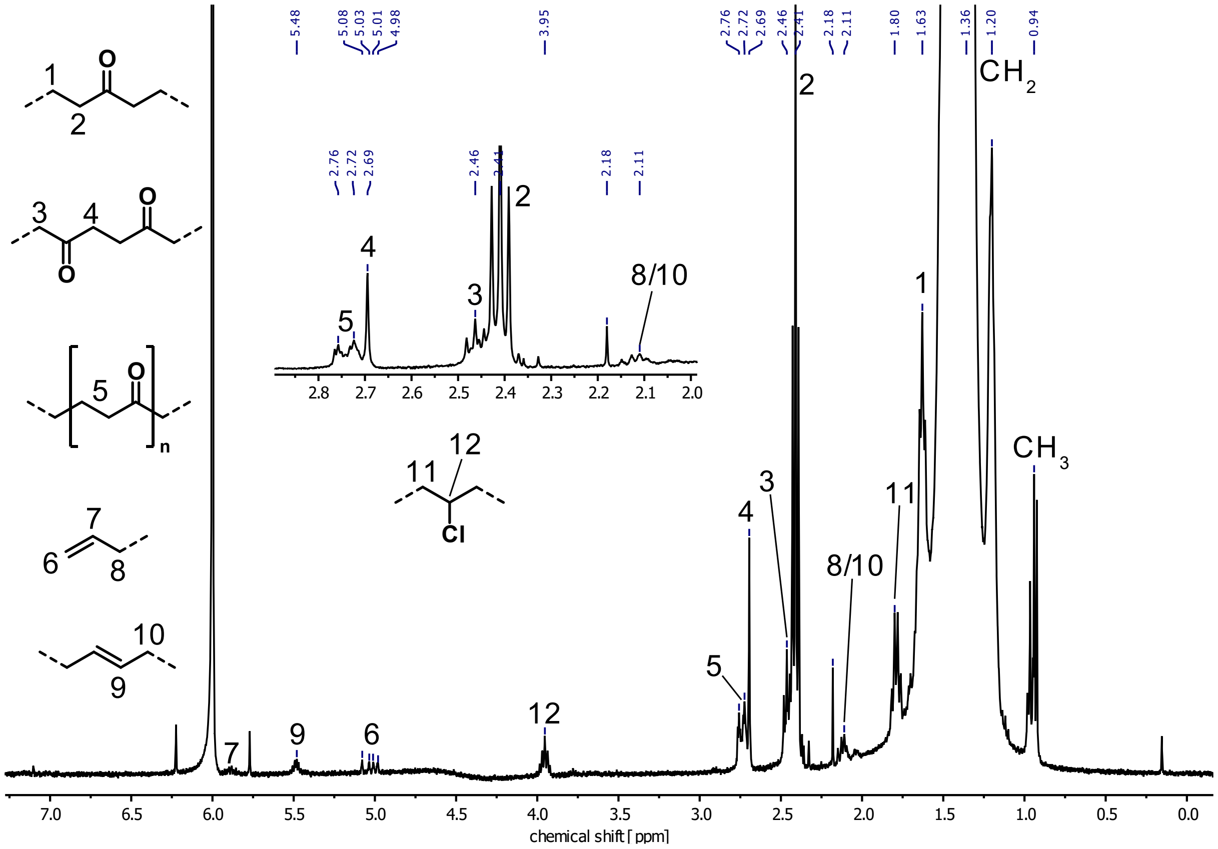

Figure S9. ^{1}H NMR spectrum ($C_2D_2Cl_4$, 373 K) of a keto-HDPE with 0.4 mol-% keto content from catalytic copolymerization prior to condensation with amines. Note the occurrence of backbone chlorination[7] by the solvent due to long measurement times at high temperature.

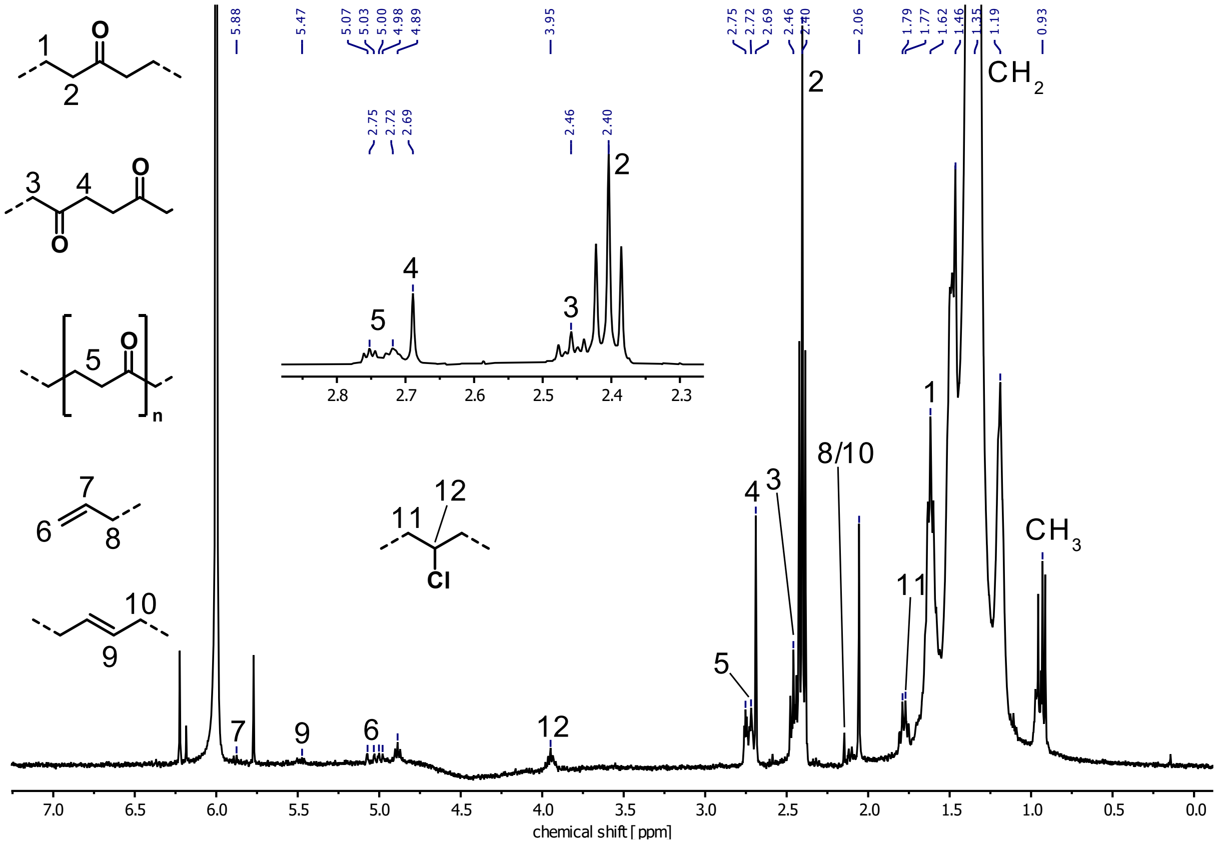

Figure S10. ^{1}H NMR spectrum ($C_2D_2Cl_4$, 373 K) of a keto-HDPE with 0.6 mol-% keto content from catalytic copolymerization prior to condensation with amines. Note the occurrence of backbone chlorination[7] by the solvent due to long measurement times at high temperature.

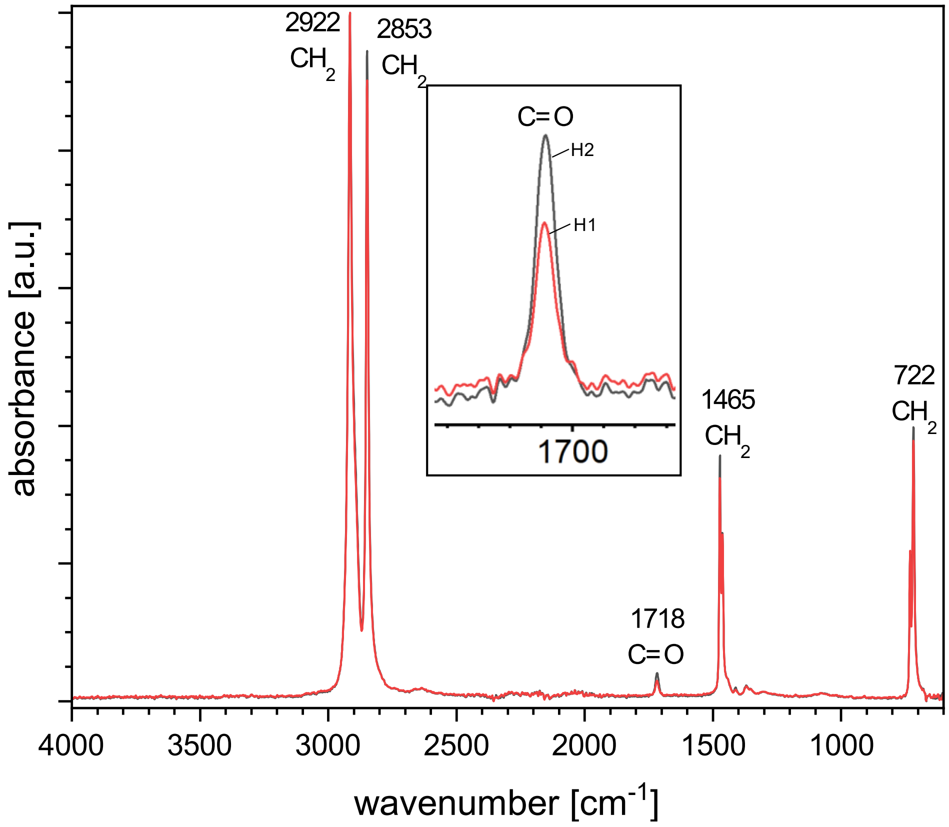

Figure S11. ATR-IR spectra of keto-HDPE (0.4 and 0.6 mol-% keto) summarized in Table S2. Spectra are normalized to the maximum at 2922 cm^{-1}.

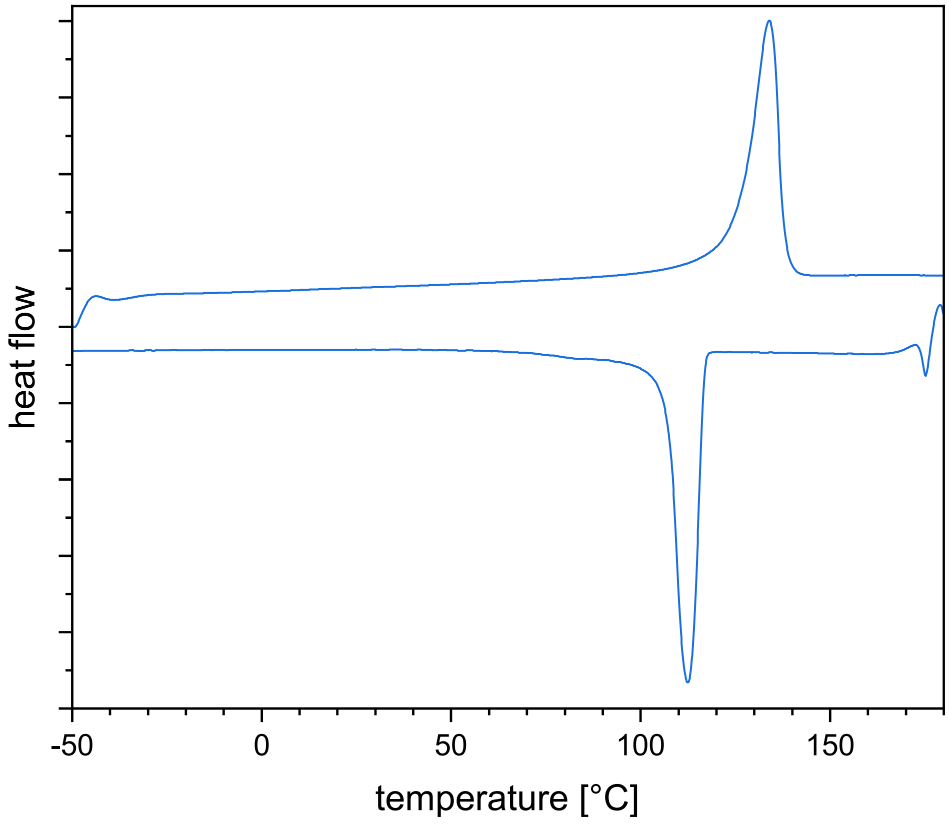

Figure S12. Exemplary DSC measurement (10 K×min^{-1}) of a keto-HDPE with 0.4 mol-% keto groups (Table S2, entry H1).

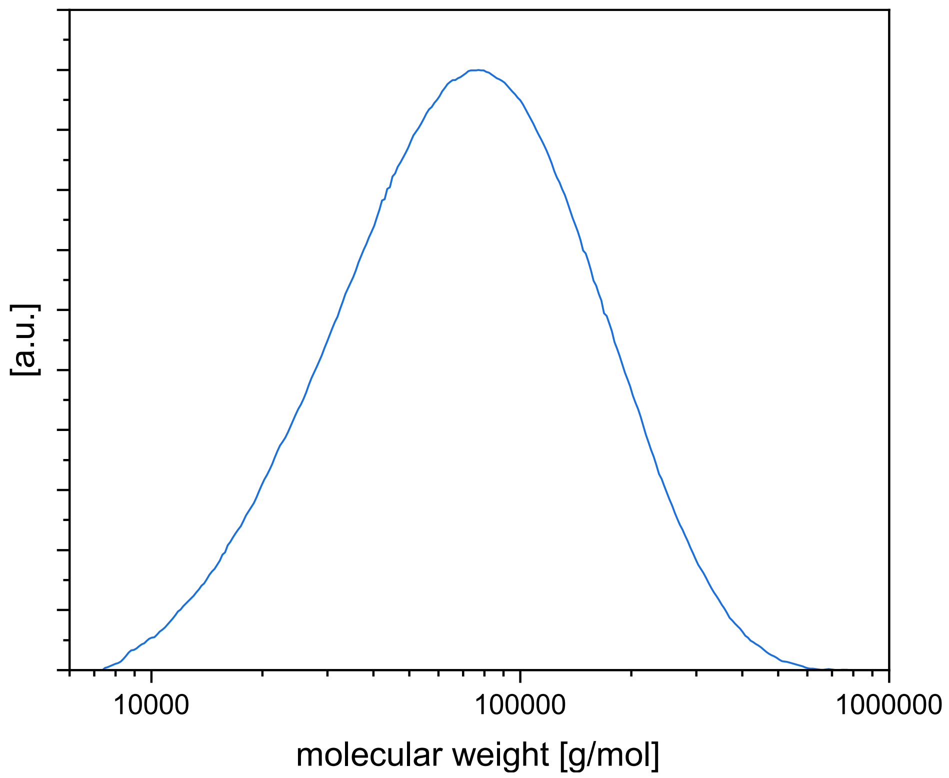

Figure S13. Exemplary GPC molecular weight distribution of keto-HDPE with 0.6 mol-% keto groups (Table S2, entry H2)

2.3 Data on low-molecular weight reference imine

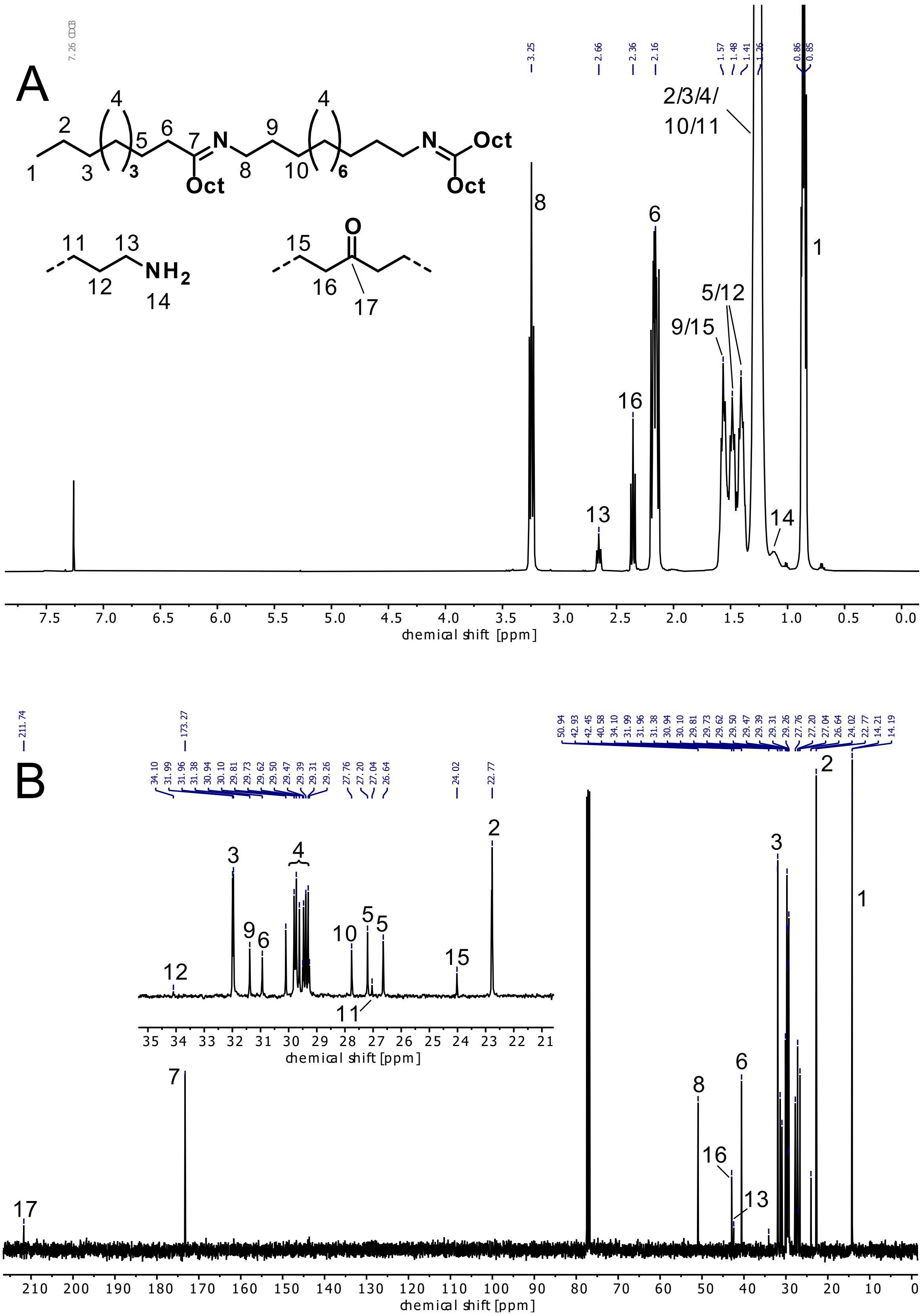

Figure S14. **A**: ^{1}H NMR and **B**: ^{13}C $\{^{1}H\}$ NMR spectrum ($CDCl_3$, 300 K) of the aliphatic diimine *N,N'*-(dodecane-1,12-diyl)bis(heptadecan-9-imine) used as reference substance. 90 % of the initial keto groups were converted to imino groups.

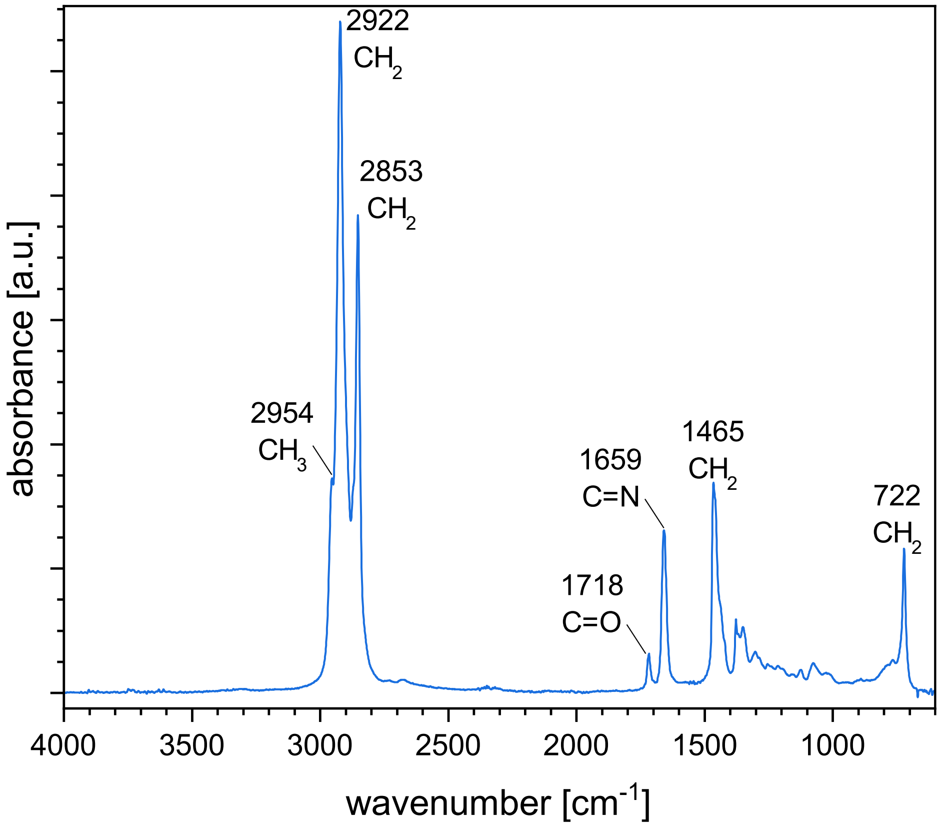

Figure S15. ATR-IR spectrum of the low-molecular weight reference imine which contains residual 9-heptadecanone (*cf.* Figure S14).

Direct quantification of imino:keto ratios (defined e.g. as the mole fraction of imine $x_{imino} = n_{imino} / (n_{imino} + n_{keto})$ by the integral intensities I_{imino} and I_{keto} of respective bands in ATR-IR is not possible because their molar absorption coefficients are different. To do so, a correction factor *a* has to be determined:

$$(S4) \quad x_{imino} = \frac{a \cdot I_{imino}}{a \cdot I_{imino} + I_{keto}}$$

If x_{imino} is known for a set of reference samples (e.g. determined by NMR), *a* can be determined from the slope of a linear regression line

$$(S5) \quad \frac{1}{a} \cdot \frac{I_{keto}}{I_{imino}} + 1 = \frac{1}{x_{imino}}$$

$$\text{with } x = \frac{I_{keto}}{I_{imino}} \text{ and } y = \frac{1}{x_{imino}}$$

The oily ketone/imine mixture obtained from the reaction described in Section 1.2.1 was mixed with defined amounts of 9-heptadecanone to prepare a suitable model system for the

detemerination of a in an all hydrocarbon environment. The mole fraction of imine in each sample was measured by ^1H NMR and compared to the respective band integrals in ATR-IR (Figure S4). The latter were determined by band deconvolution with two Lorentzian functions at 1718 and 1659 cm^{-1} as described in Section 1.1.2 (Figure S16).

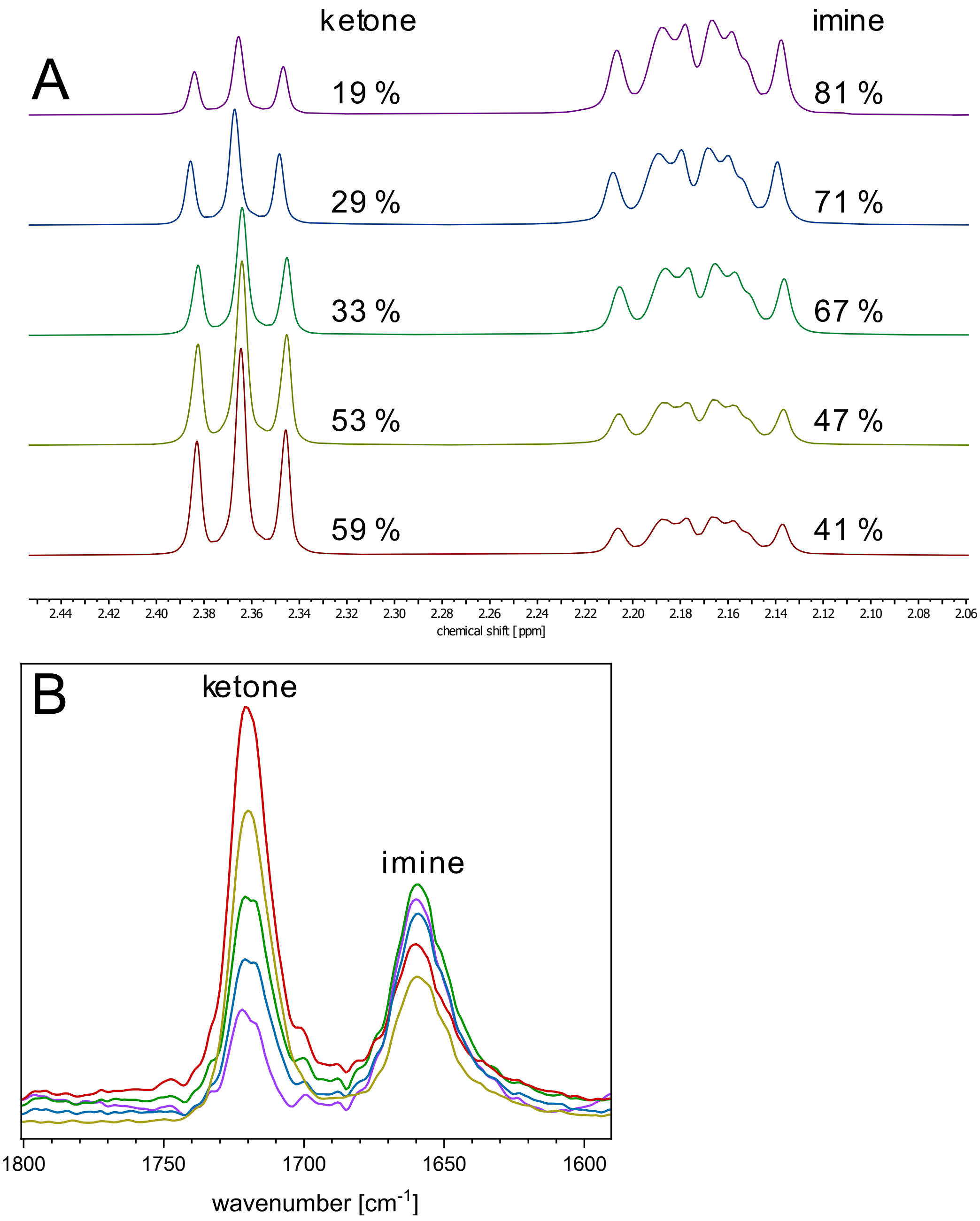

Figure S16. **A**: ^1H NMR ($CDCl_3$, 300 K, normalized to the CH_2 backbone peak at 1.26 ppm) and **B**: ATR-IR spectra (normalized to CH_2 band at 2922 cm^{-1}) of physical mixtures of 9-heptadecanone and diimine *N,N'*-(dodecane-1,12-diyl)bis(heptadecan-9-imine).

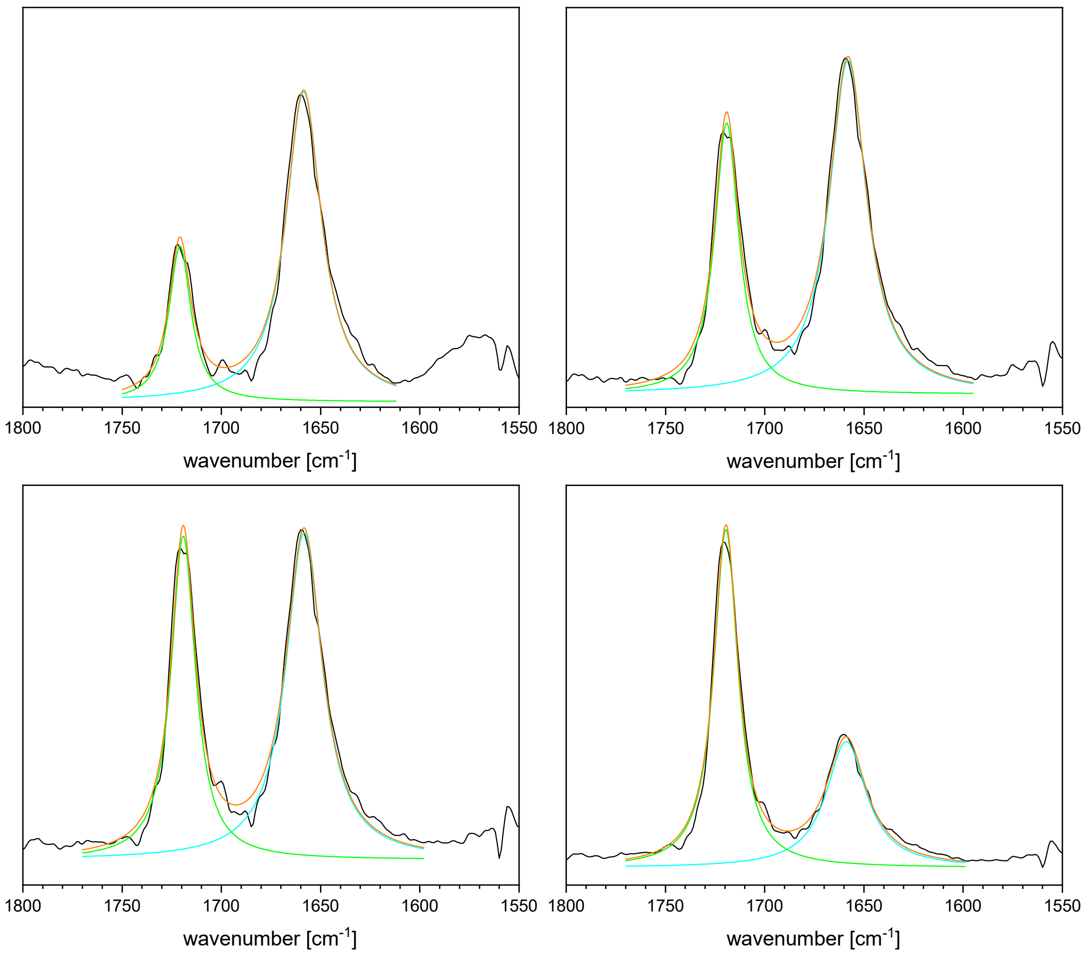

Figure S17. Exemplary ATR-IR band deconvolutions of imino (1659 cm^{-1}) and keto (1718 cm^{-1}) bands with two Lorentzian functions for mixtures of 9-heptadecanone and diimine *N,N'*-(dodecane-1,12-diyl)bis(heptadecan-9-imine).

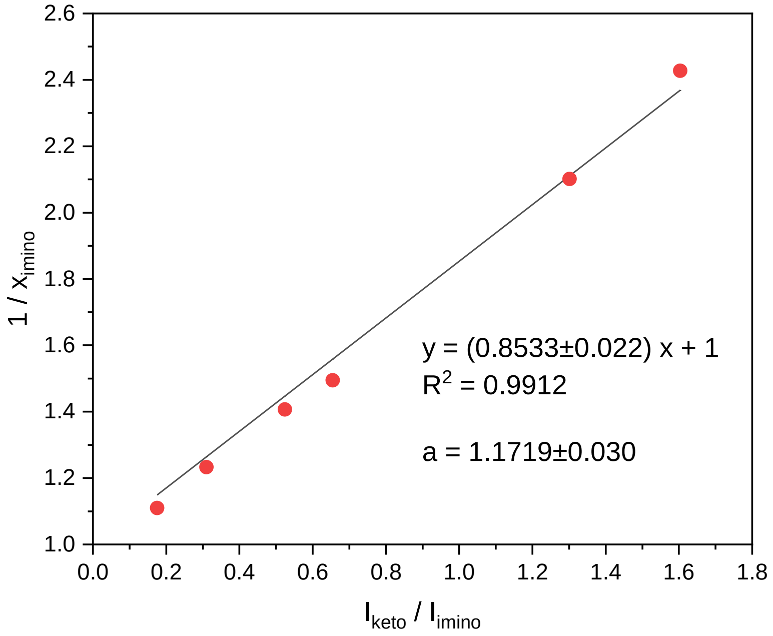

Figure S18. Linear regression for the determination of the molar absorption coefficient ratio of keto vs. imino groups $\varepsilon_{keto}/\varepsilon_{imino}$ in aliphatic surroundings. ε_{keto} is larger by a factor of 1.172 ± 0.03.

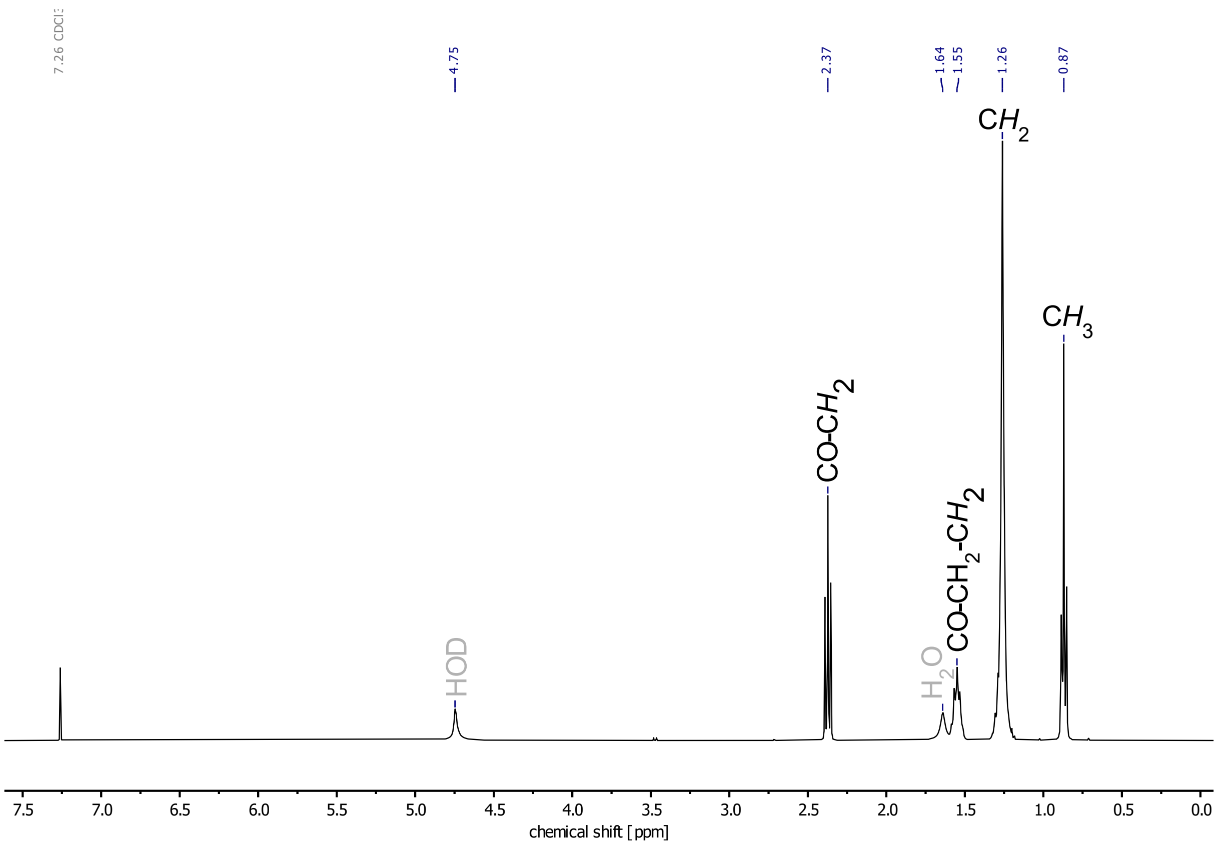

Figure S19. ^{1}H NMR spectrum ($CDCl_3$, 300 K) of hydrolyzed *N,N'*-(dodecane-1,12-diyl)bis (heptadecan-9-imine) after extraction of the crude aqueous mixture with $CDCl_3$ shows signals exclusively attributed to 9-heptadecanone. Hydrolysis conditions: 25 mg of diimine in 6 mL of H_2O (ca. 4500 equiv.), pH = 1 (HCl, 8 equiv.), 5 min, 95 °C. The other hydrolysis product (dodecane-1,12-diammonium chloride) remains in the aqueous phase during extraction with deuterated chloroform.

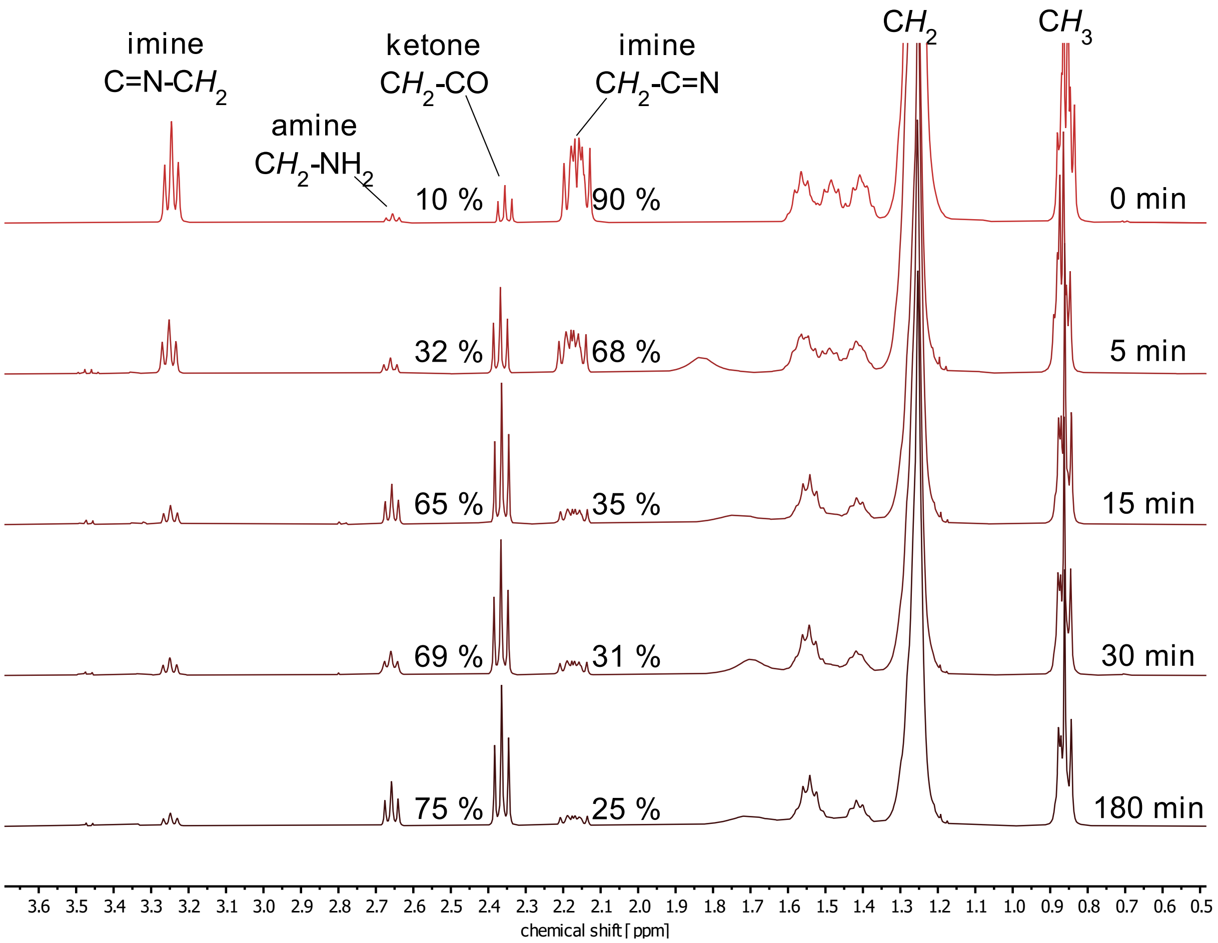

Figure S20. ^{1}H NMR spectra ($CDCl_3$, 300 K) of the hydrolysis of *N,N'*-(dodecane-1,12-diyl)bis (heptadecan-9-imine) after different reaction times in neat water. Spectra are normalized to the CH_2 backbone peak at 1.26 ppm. The individual crude aqueous reaction mixtures were quickly cooled to r.t. after the given time intervals, extracted with $CDCl_3$ and then measured directly. Hydrolysis conditions: 25 mg of diimine in 6 mL of neat H_2O (ca. 4500 equiv.), 95 °C. Imine hydrolysis in neat water is considerably slower as compared to reactions in the presence of an acid (*cf.* Figure S19). In the absence of acid, the hydrolysis reaction ceases after a certain time due to gradually increasing pH (up to 11-12 in this case) caused by the released amine hydrolysis product.

Note that the separation of the two hydrolysis products is facilitated under acidic conditions, since the protonated diamine remains dissolved in the aqueous phase whereas 9-heptadecanone forms a hydrophobic phase. The latter can be efficiently extracted with organic solvent (Figure S19). In contrast, the diamine obtained from hydrolysis reactions at neutral/basic pH was soluble in the organic phase (Figure S20), so that no direct separation of ketone and amine was possible in this case.

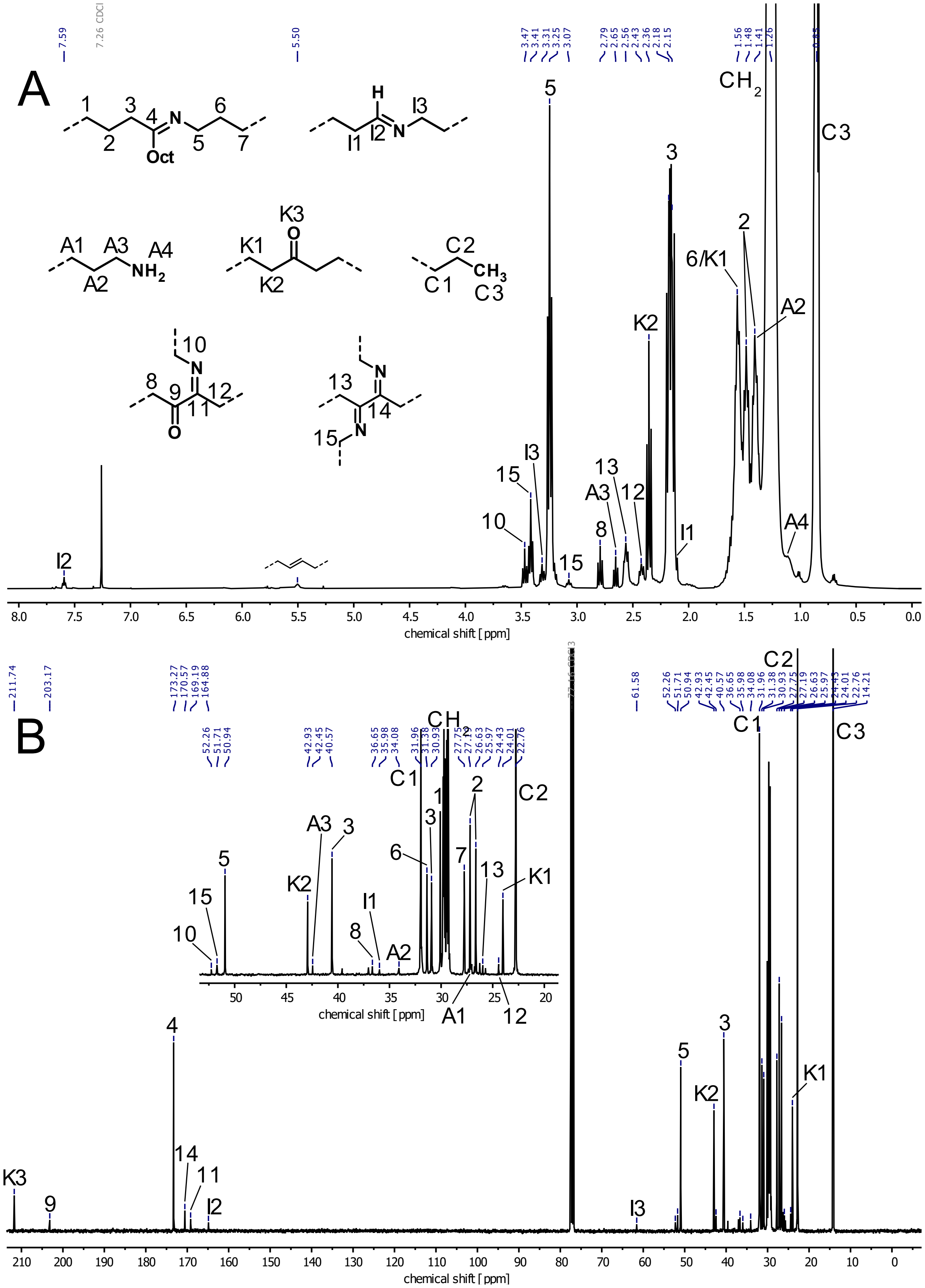

Figure S21. **A**: ^{1}H NMR and **B**: ^{13}C {^{1}H} NMR spectrum (CDCl$_3$, 300 K) of *N,N'*-(dodecane-1,12-diyl)bis(heptadecan-9-imine) synthesized in the presence of oxygen at 120 °C. Besides the product and residual educts, side products from oxidation of amine and imine are observed.

The condensation of aliphatic ketones and amines at elevated temperatures is sensitive to oxidation by air oxygen (*cf.* Figure S21). To understand how the observed oxidation side products form and which functional groups are prone to oxidation, the following substances/mixtures were exposed to air at 120 °C for 7 h:

1) 9-heptadecanone
2) 1 equiv. 1,12-diaminododecane + 2 equiv. heptadecane
3) 1 equiv. 1,12-diaminododecane + 2 equiv. 9-heptadecanone
4) *N,N'*-(dodecane-1,12-diyl)bis(heptadecan-9-imine) + traces of 1,12-diaminododecane and 9-heptadecanone

1,12-Diaminododecane was mixed with heptadecane to obtain a homogeneous melt and to simulate the diluting effect of the 9-heptadecanone present during the imine synthesis. No oxidation reaction was observed in 1), whereas formation of an aldimine was observed in 2) (*cf.* Figure S22).

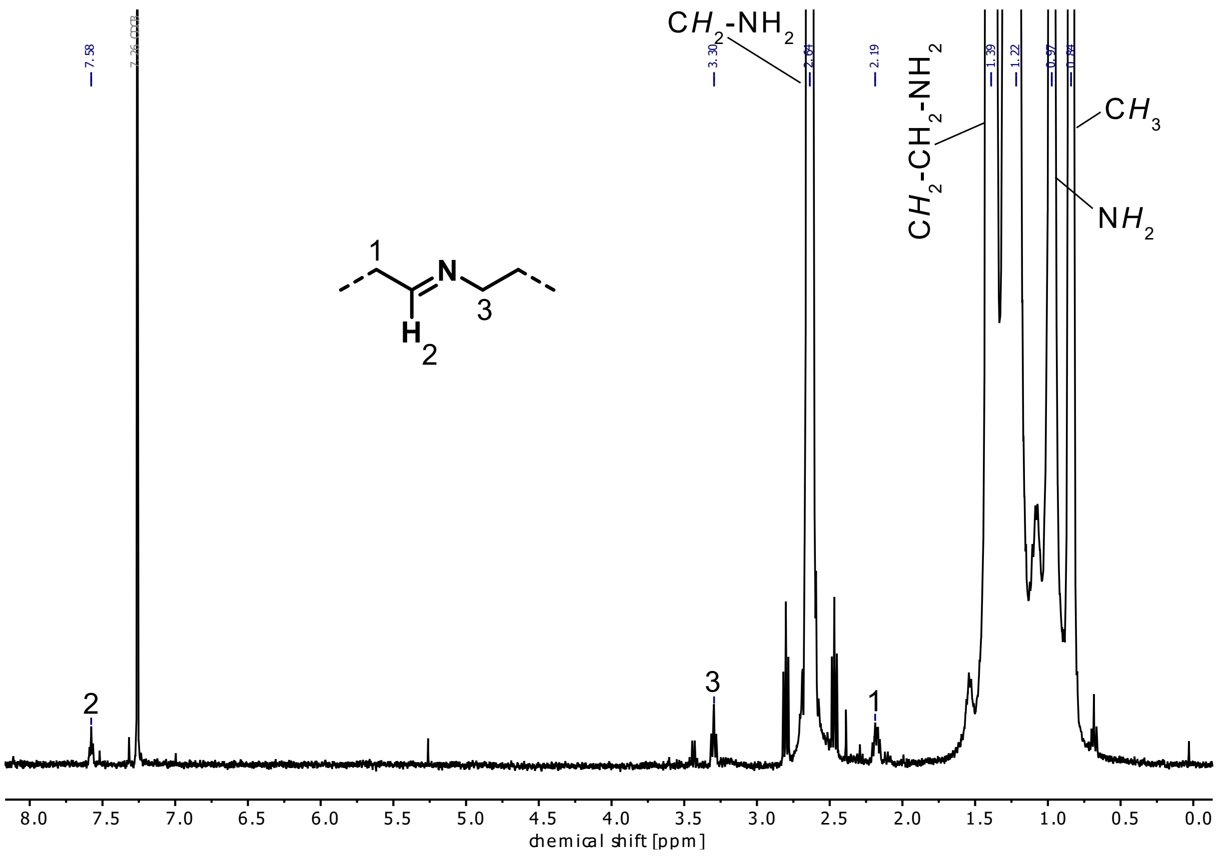

Figure S22. ^{1}H NMR spectrum ($CDCl_3$, 300 K) of 1,12-diaminododecane (+ heptadecane) containing an aldimine oxidation product after exposure to air at 120 °C for 7 h.

Sample 3) contained 11 mol-% of aldimine, 78 mol-% of α-keto imine and 11 mol-% of α-diimine. In contrast, sample 4) contained no aldimine, 59 mol-% of α-keto imine and 41 mol-% of α-diimine. These observations imply that aldimines are formed exclusively by oxidation of amino groups (*cf.* Figure S23) during the condensation reaction. Imino groups are oxidized in

α-position to yield α-keto imines which can then react with amino groups to form α-diimines. Note that NMR spectra indicated that, proceeding from the identified species, further decomposition and oxidation reactions occur to yield yet unknown olefinic species. In summary, these experiments underline the importance of O_2-free conditions for all reactions involving imino and amino groups at elevated temperatures.

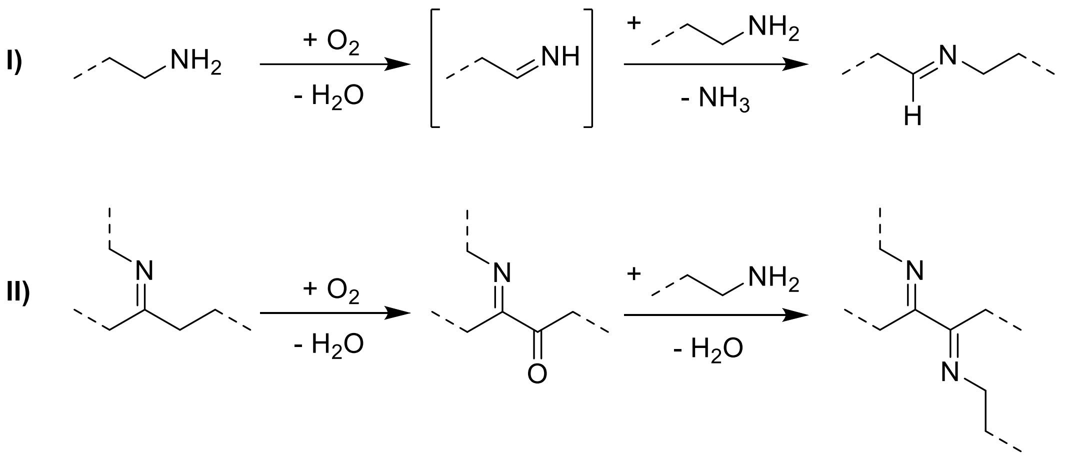

Figure S23. Oxidative side reactions during the condensation of keto-PEs and aliphatic amines in the presence of oxygen at elevated temperatures.

2.4 Data on condensation products with monoamine

Table S3. Summarized data of imino-functionalized LDPEs from condensation of keto-LDPEs with 1-aminotetradecane.

#	initial keto-LDPE (keto mol-%)	T_m [°C] (% cryst.)[a]	T_c [°C] (% cryst.)[b]	molar ratio imino:keto[c]	χ(C=O) [mol-%][d]	χ(C=N) [mol-%][d]
I1	L3 (2.2)	102 (35)	88 (39)	68 : 32	0.7	1.4
I2	L4 (4.9)	92 (27)	76 (29)	71 : 29	0.9	2.1
I3	L6 (7.6)	9, 51, 78 (25)	2, 39, 61 (27)	80 : 20 (80 : 20)	0.9 (1.2)	3.5 (5.8)[e]
I4	L7 (12.6)	7, 51 (19)	-1, 39 (21)	83 : 17 (82 : 18)	1.2 (1.7)	5.9 (7.7)[f]

Condensation conditions: 100 mg of keto-LDPE, 4 equiv. of amine with respect to keto groups, 160 °C, 50 rpm, gradually reduced pressure over 7 h, 2 h at 2×10^{-2} mbar. [a]Peak melting point and degree of crystallinity determined by DSC, 2nd heating cycle (10 K×min^{-1}). [b]Peak crystallization point and degree of crystallinity determined by DSC, 1st cooling cycle (10 K×min^{-1}). [c]Determined by band deconvolution of ATR-IR spectra considering the different molar absorption coefficients of imino and keto groups (*cf.* Figure S18). Values in brackets are determined by ^{1}H-NMR in $CDCl_3$ (300 K). [d]Keto and imino content with respect to ethylene repeat units of the polymer. Determined by ATR-IR spectra. Values in brackets are determined by ^{1}H-NMR in $CDCl_3$ (300 K). [e]0.3 mol-% of pyrrole according to ^{1}H-NMR. [f]0.9 mol-% of pyrrole according to ^{1}H-NMR.

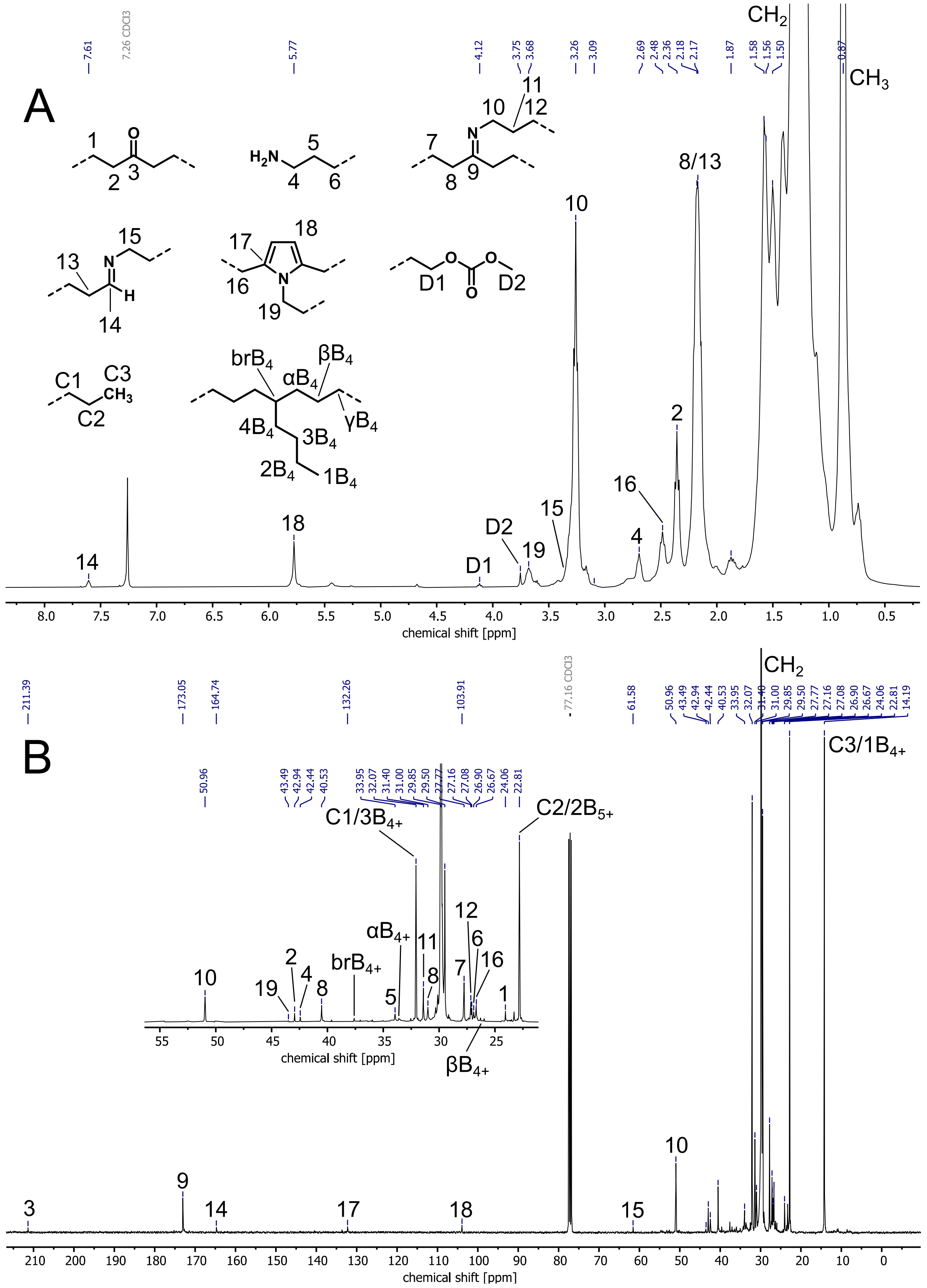

Figure S24. **A**: ^{1}H NMR and **B**: ^{13}C $\{^{1}H\}$ NMR spectrum ($CDCl_3$, 300 K) of imino-functionalized LDPE synthesized by condensation of keto-LDPE (7.6 mol-% keto groups) with 1-aminotetradecane (Table S3, entry I3).

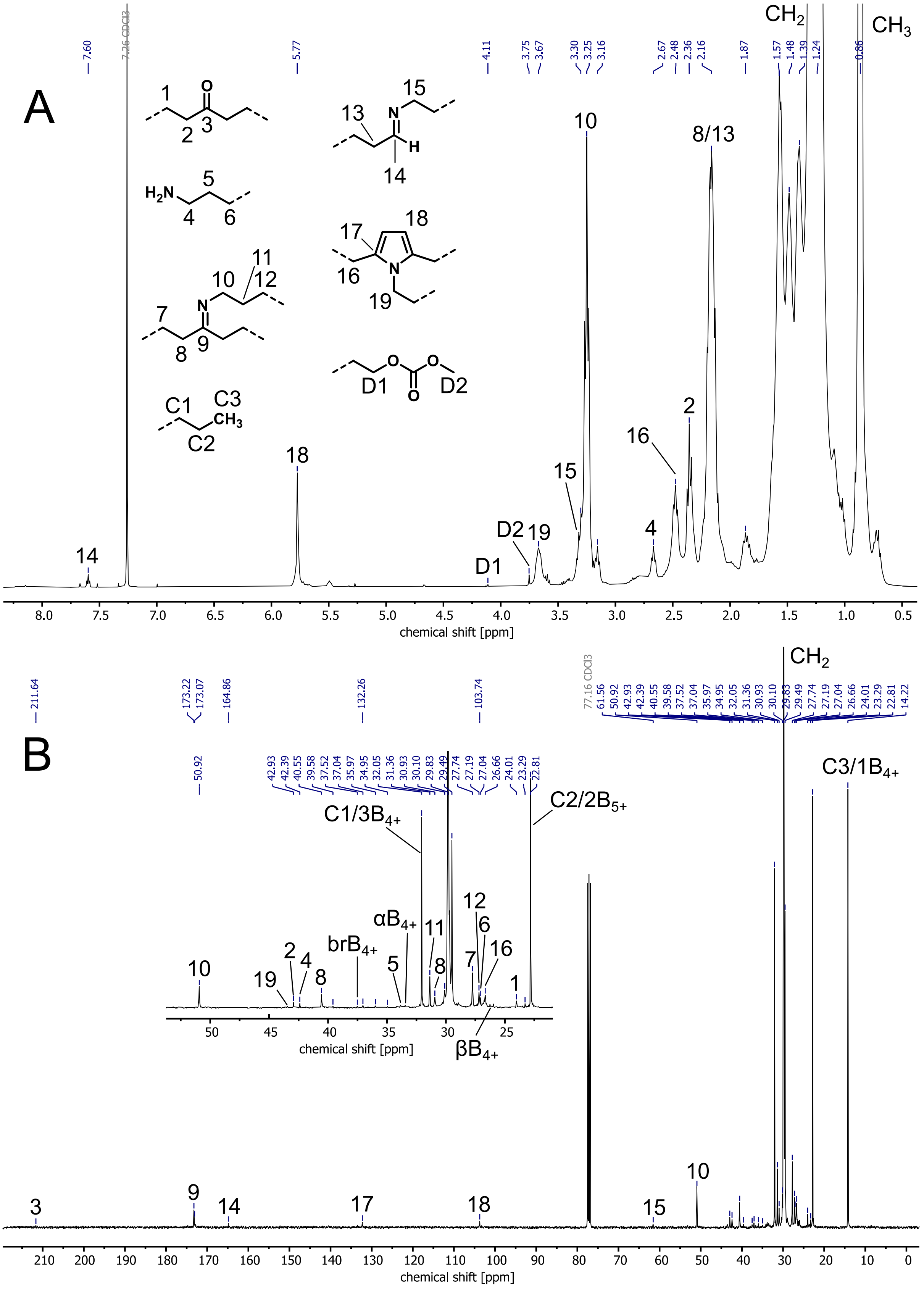

Figure S25. A: ^{1}H NMR and **B**: ^{13}C {^{1}H} NMR spectrum ($CDCl_3$, 300 K) of imino-functionalized LDPE synthesized by condensation of keto-LDPE (12.6 mol-% keto groups) with 1-aminotetradecane (Table S3, entry I4).

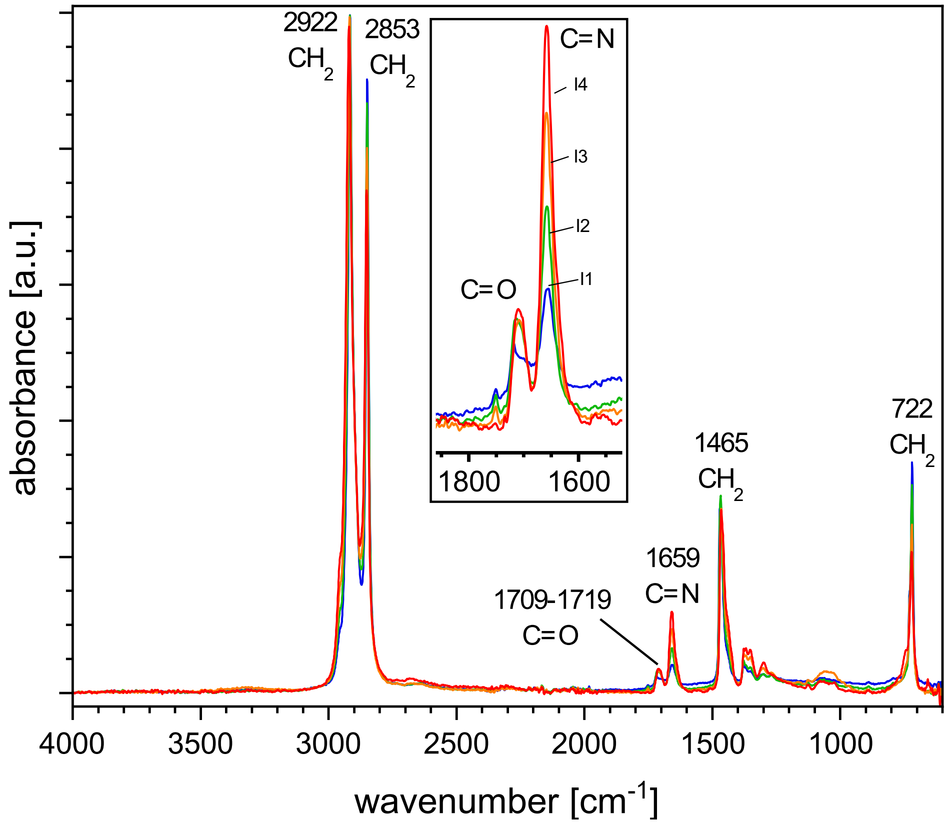

Figure S26. ATR-IR spectra of imino-functionalized LDPEs (0.7 – 1.2 mol-% keto, 1.4 – 5.9 mol-% imino groups) summarized in Table S3. Spectra are normalized to the maximum at 2922 cm^{-1}.

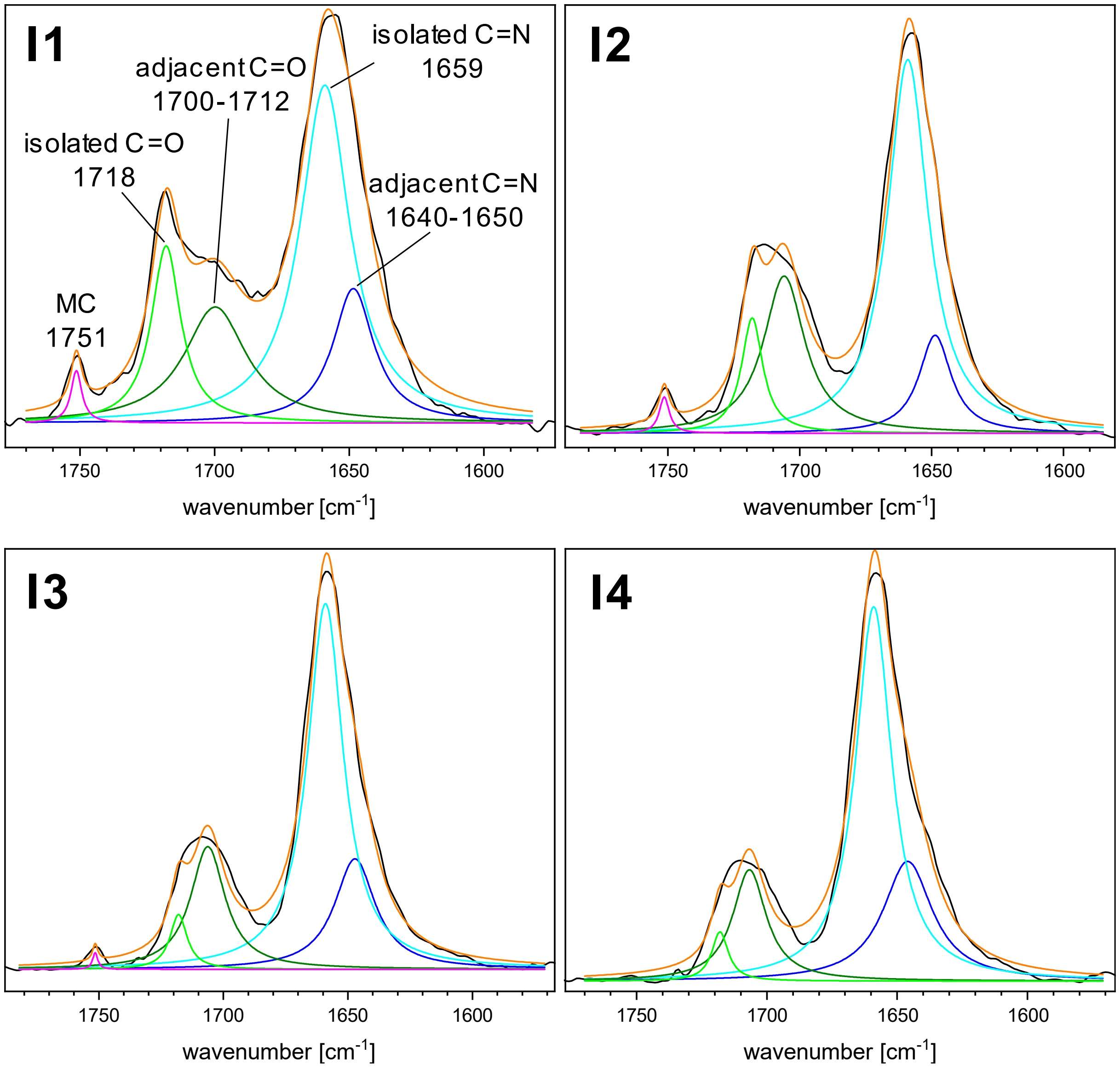

Figure S27. ATR-IR deconvolution of bands evoked by isolated imino groups (1659 cm^{-1}), imino groups in close proximity to ketones/imines (1640 – 1650 cm^{-1}), isolated keto groups (1718 cm^{-1}), keto groups in close proximity to ketones/imines (1700 – 1712 cm^{-1}) and methyl carbonate end groups (MC, 1751 cm^{-1}) with five Lorentzian functions for imino-functionalized LDPEs I1 – I4 of Table S3. Carbonate groups are incorporated into the polymer by chain transfer reactions to the solvent dimethyl carbonate during synthesis of the initial keto-LDPEs.

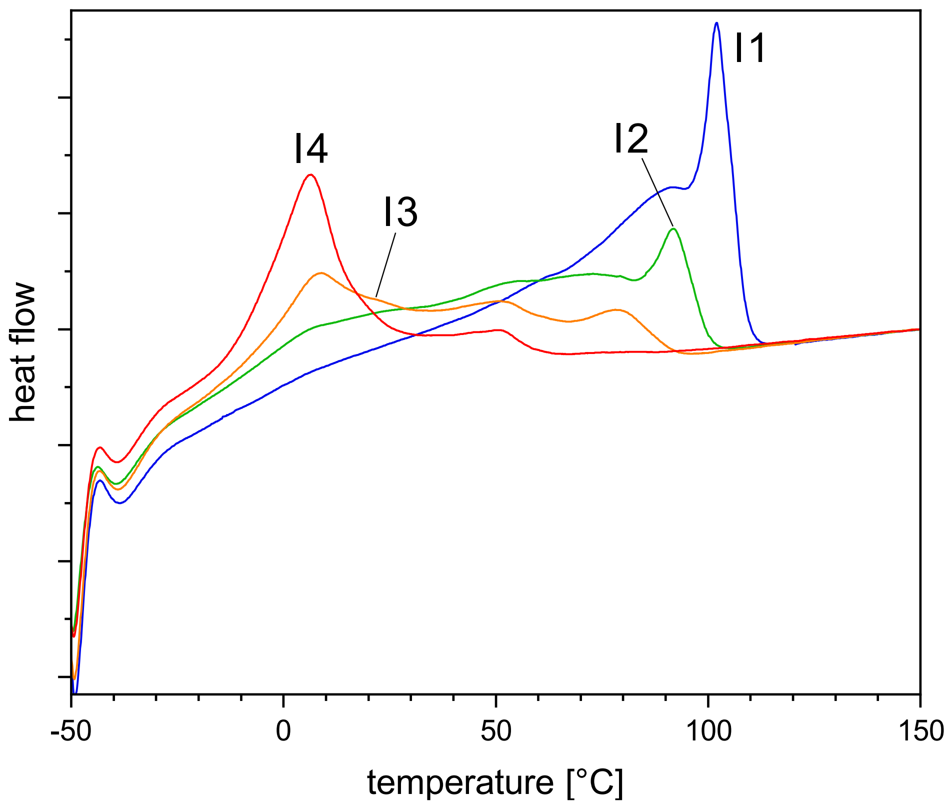

Figure S28. 2nd DSC heating curves of imino-functionalized LDPEs (1.4 – 5.9 mol-% imino) of Table S3.

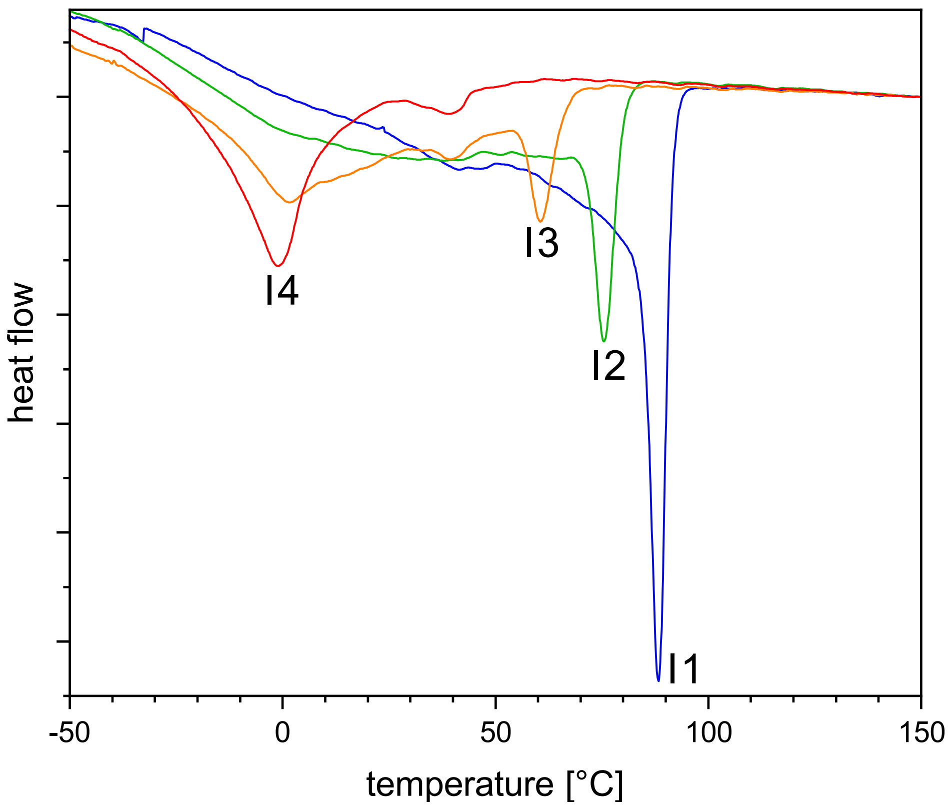

Figure S29. 1st DSC cooling curves of imino-functionalized LDPEs (1.4 – 5.9 mol-% imino) of Table S3.

2.5 Crosslinking with diamine on small scale

Table S4. Summarized data of imino-crosslinked LDPEs from preliminary condensations of keto-LDPEs with 1,12-diaminododecane on small scale. Non-crosslinked keto-LDPEs L3, L4, L6 and L7 are given for comparison.

#	initial keto-LDPE (keto mol-%)	amount of amine (NH_2 : keto) [equiv.]	T_m [°C] (% cryst.)[a]	molar ratio imino:keto[b]	χ(C=O) [mol-%][c]	χ(C=N) [mol-%][c]	gel fraction [wt.-%][d]
L3	-	-	108 (33)	only keto	2.2	0	0
L4	-	-	105 (36)	only keto	4.9	0	0
L6	-	-	102 (28)	only keto	7.6	0	0
L7	-	-	97 (25)	only keto	12.6	0	0
XI1	L3 (2.2)	1.00	109 (34)	- [e]	1.6[e]	- [e]	70
XI2	L4 (4.9)	1.00	89 (21)	57 : 43	1.5	2.0	97
XI3	L6 (7.6)	1.00	73 (13)	67 : 33	1.4	2.8	97
XI4	L7 (12.6)	1.00	3 (1)	66 : 34	2.3	4.5	95
XI5	L3 (2.2)	1.05	106 (35)	- [e]	1.2[e]	- [e]	66
XI6	L4 (4.9)	1.05	92 (25)	- [e]	1.2[e]	- [e]	86
XI7	L6 (7.6)	1.05	76 (15)	- [e]	1.6[e]	- [e]	86
XI8	L7 (12.6)	1.05	-2 (<1)	- [e]	1.9[e]	- [e]	88
XI9	L3 (2.2)	2.00	101 (30)	- [e]	0.5[e]	- [e]	86
XI10	L4 (4.9)	2.00	80 (20)	- [e]	1.1[e]	- [e]	95
XI11	L6 (7.6)	2.00	57 (11)	- [e]	1.0[e]	- [e]	93
XI12	L7 (12.6)	2.00	-2 (3)	- [e]	1.1[e]	- [e]	94

Condensation conditions: 100 mg of keto-LDPE, 160 °C, 50 rpm, gradually reduced pressure over 7 h, 2 h at 2×10^{-2} mbar. [a]Peak melting point and degree of crystallinity determined by DSC, 2nd heating cycle (10 $K\times min^{-1}$). [b]Determined by band deconvolution of ATR-IR spectra considering the different molar absorption coefficients of imino and keto groups (*cf.* Figure S18). [c]Keto and imino content with respect to ethylene repeat units of the polymer. Determined by ATR-IR spectra. [d]Determined by weighing the samples before and after extraction with hot toluene (6 h, 110 °C). [e]Quantification of imino content from ATR-IR not possible due to a considerable number of amino groups in the sample. Keto content was estimated from the ratio of the C=O band intensity vs. the CH_2 band intensity (2922 cm^{-1}) and the method of Figure S1.

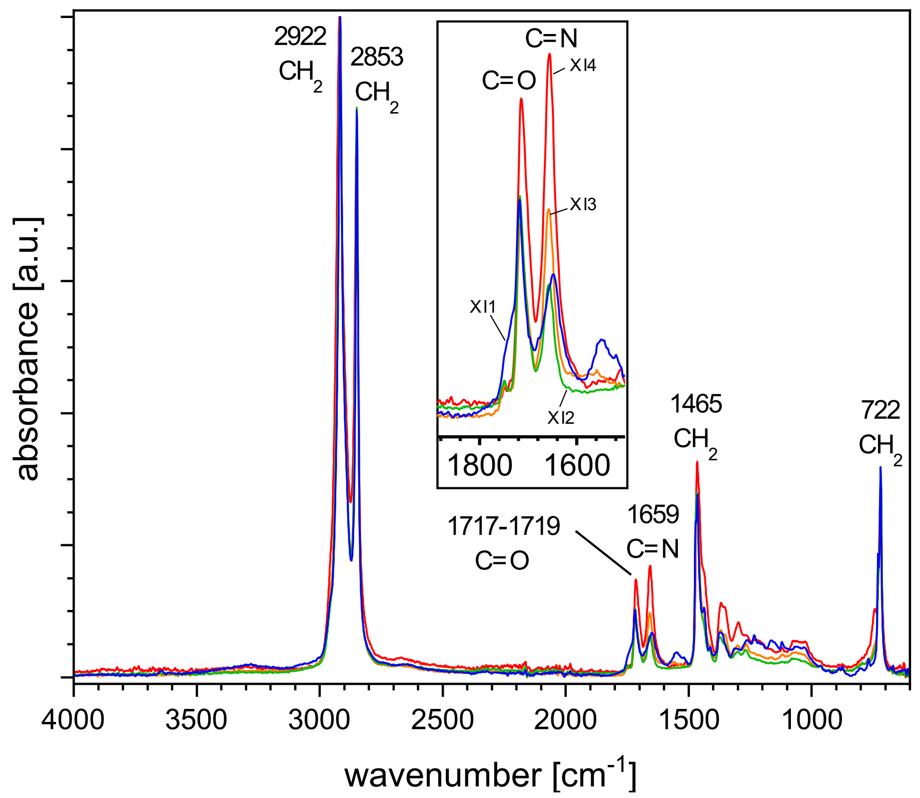

Figure S30. ATR-IR spectra of imino-crosslinked LDPEs synthesized with a 1:1 ratio of NH_2 to C=O (1.4 – 2.3 mol-% keto, 2.0 – 4.5 mol-% imino groups) summarized in Table S4. Spectra are normalized to the maximum at 2922 cm^{-1}.

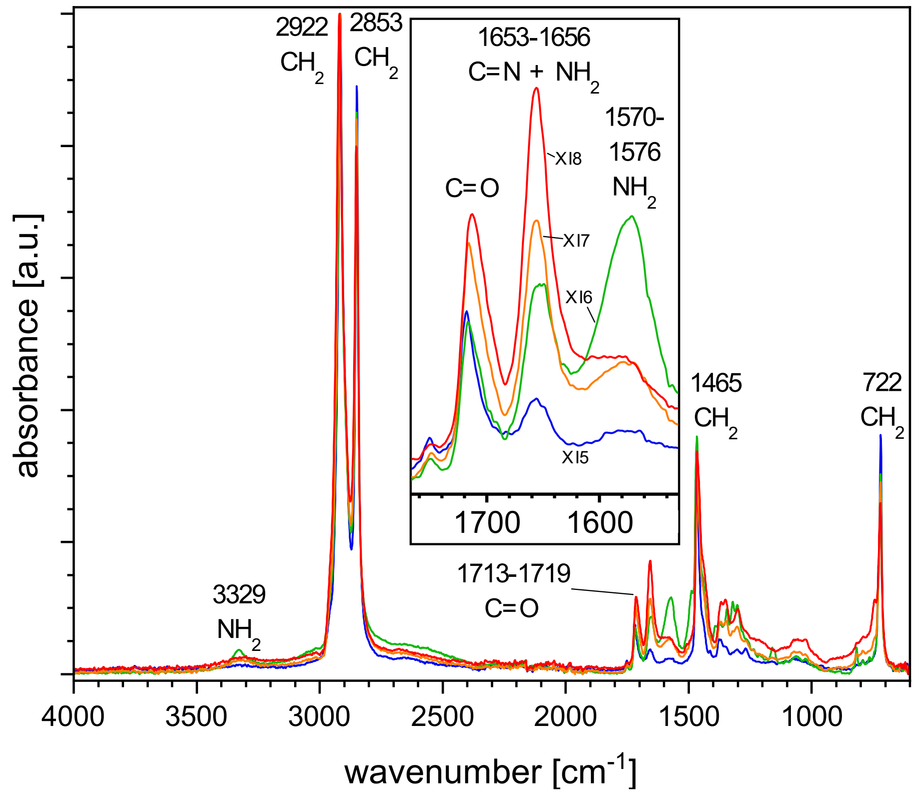

Figure S31. ATR-IR spectra of imino-crosslinked LDPEs synthesized with a 1.05:1.00 ratio of NH_2 to C=O summarized in Table S4. Spectra are normalized to the maximum at 2922 cm^{-1}.

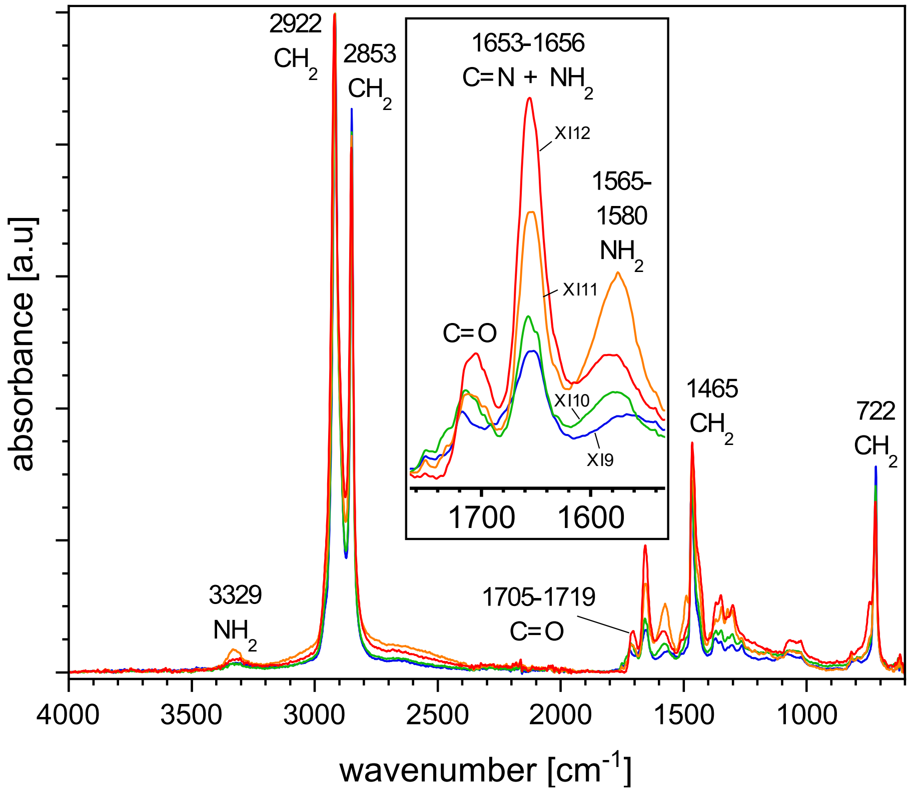

Figure S32. ATR-IR spectra of imino-crosslinked LDPEs synthesized with a 2:1 ratio of NH_2 to C=O summarized in Table S4. Spectra are normalized to the maximum at 2922 cm^{-1}.

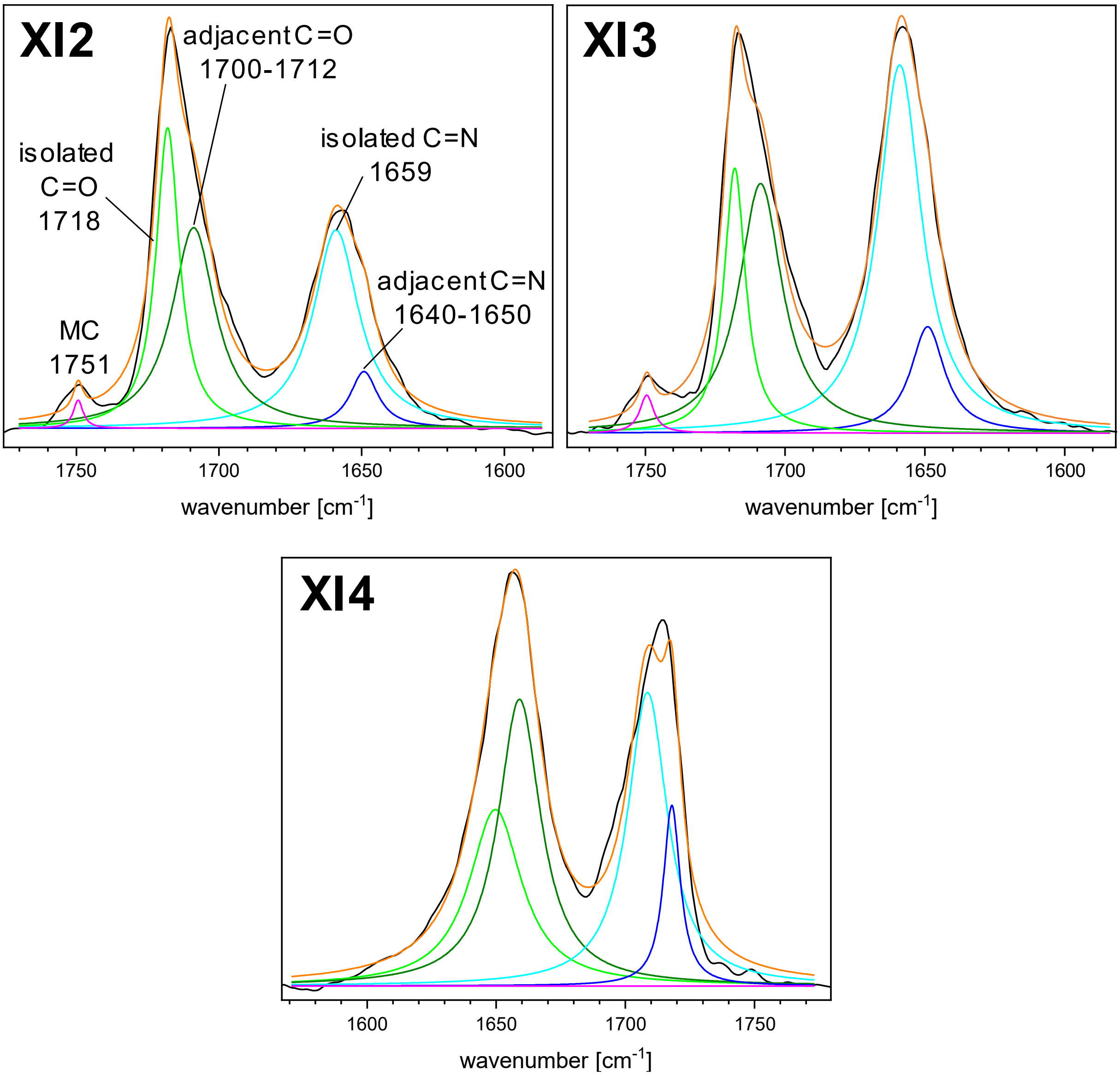

Figure S33. Exemplary ATR-IR deconvolution of bands evoked by isolated imino groups (1659 cm^{-1}), imino groups in close proximity to ketones/imines (1640 – 1650 cm^{-1}), isolated keto groups (1718 cm^{-1}), keto groups in close proximity to ketones/imines (1700 – 1712 cm^{-1}) and methyl carbonate end groups (MC, 1751 cm^{-1}) with five Lorentzian functions for imino-crosslinked LDPEs XI2 – XI4 of Table S4. Carbonate groups are incorporated into the polymer by chain transfer reactions to the solvent dimethyl carbonate during synthesis of the initial keto-LDPEs.

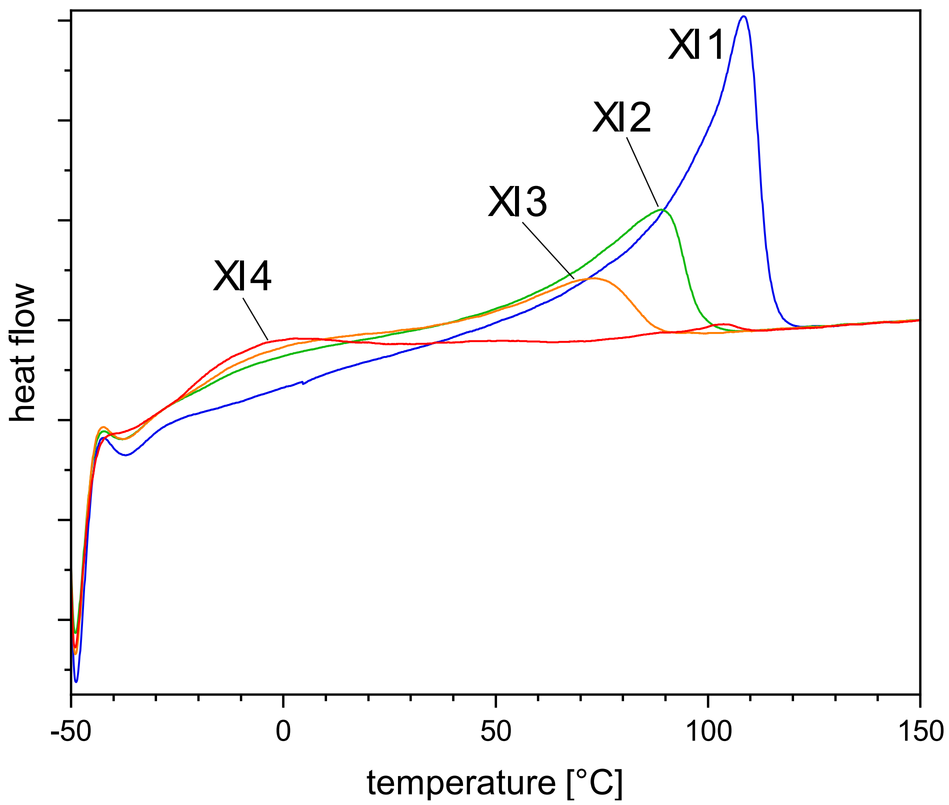

Figure S34. Exemplary 2nd DSC heating curves of imino-crosslinked LDPEs (2.0 – 4.5 mol-% imino) of Table S4.

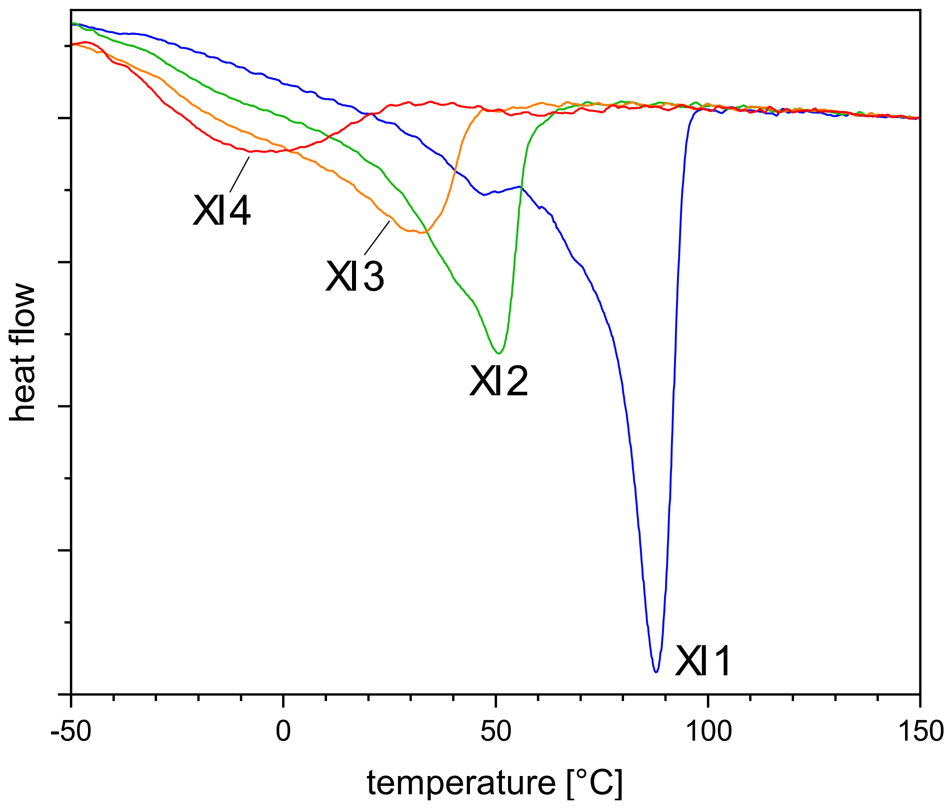

Figure S35. Exemplary 1st DSC cooling curves of imino-crosslinked LDPEs (2.0 – 4.5 mol-% imino) of Table S4.

2.6 Crosslinking in molds to prepare tensile specimens

Table S5. Summarized data of imino-crosslinked PEs from either direct condensation of keto-PEs with 1,12-diaminododecane (Method 1, suitable for LDPEs) or condensation via a two-step procedure over an amino-functionalized PE intermediate (Method 2, suitable for LDPEs and HDPEs). Non-crosslinked keto-LDPEs L1 – L4 and keto-HDPEs H1, H2 are given as reference.

#	initial keto-PE (keto mol-%)	crosslinking method	T_m [°C] (% cryst.)[a]	molar ratio imino:keto[b]	χ(C=O) [mol-%][c]	χ(C=N) [mol-%][c]	gel fraction [wt.-%][d]
L1	-	-	109 (44)	only keto	0.7	0	0
L2	-	-	109 (37)	only keto	1.2	0	0
L3	-	-	108 (33)	only keto	2.2	0	0
L4	-	-	105 (36)	only keto	4.9	0	0
H1	-	-	134 (68)	only keto	0.4	0	0
H2	-	-	135 (67)	only keto	0.6	0	0
XIL1	L1 (0.7)	1	111 (38)	40 : 60	0.6	0.4	28
XIL2	L2 (1.2)	1	111 (35)	46 : 54	0.7	0.6	35
XIL3	L3 (2.2)	1	106 (32)	60 : 40	0.9	1.4	66
XIL4	L4 (4.9)	1	96 (24)	39 : 61	2.3	1.5	86
XIL5	L1 (0.7)	2	112 (39)	17 : 83	0.8	0.2	39
XIL6	L2 (1.2)	2	111 (37)	22 : 78	0.7	0.2	11
XIL7	L3 (2.2)	2	105 (31)	44 : 56	1.0	0.8	54
XIH1	H1 (0.4)	2	136 (61)	57 : 43	0.2	0.2	27
XIH2	H2 (0.6)	2	136 (53)	74 : 26	0.2	0.5	85

For details on condensation conditions by Method 1 or 2 *cf.* Section 1.2.4. [a]Peak melting point and degree of crystallinity determined by DSC, 2nd heating cycle (10 K×min^{-1}). [b]Determined by band deconvolution of ATR-IR spectra considering the different molar absorption coefficients of imino and keto groups (*cf.* Figure S18). [c]Keto and imino content with respect to ethylene repeat units of the polymer. Determined by ATR-IR spectra acquired on newly cut cross sectional areas of the specimens. [d]Determined by weighing the samples before and after extraction with hot toluene (6 h, 110 °C).

Table S6. Mechanical properties in tensile testing of imino-crosslinked PEs and respective non-crosslinked keto-PE references.[a]

<table>
<tr><th>#</th><th>χ(C=O) [mol-%][b]</th><th>χ(C=N) [mol-%][b]</th><th>gel fraction [wt.-%][c]</th><th>E_t [MPa]</th><th>ε_{max} [%]</th><th>σ_{max} [MPa]</th><th>σ_Y [MPa]</th><th>ε_b [%]</th></tr>
<tr><td>L1</td><td>0.7</td><td>0</td><td>0</td><td>191±27</td><td>9.7±1.7</td><td colspan="2">9.7±0.5</td><td>11.7±2.4</td></tr>
<tr><td>L2</td><td>1.2</td><td>0</td><td>0</td><td>215±51</td><td>11.8±0.5</td><td colspan="2">10.4±0.5</td><td>13.5±1.1</td></tr>
<tr><td>L3</td><td>2.2</td><td>0</td><td>0</td><td>243</td><td>5.1</td><td colspan="2">7.4</td><td>5.4</td></tr>
<tr><td>L4</td><td>4.9</td><td>0</td><td>0</td><td>239±48</td><td>10.6±1.6</td><td colspan="2">9.3±0.3</td><td>12.4±2.4</td></tr>
<tr><td>XIL1</td><td>0.6</td><td>0.4</td><td>28</td><td>249 ±2</td><td>11.6±2.9</td><td colspan="2">10.2±0.1</td><td>43±21</td></tr>
<tr><td>XIL2</td><td>0.7</td><td>0.6</td><td>35</td><td>201±13</td><td>29±12</td><td colspan="2">9.7±0.2</td><td>37±12</td></tr>
<tr><td>XIL3</td><td>0.9</td><td>1.4</td><td>66</td><td>104±14</td><td>54.7±8.0</td><td colspan="2">8.1±0.3</td><td>58.4±7.6</td></tr>
<tr><td>XIL4</td><td>2.3</td><td>1.5</td><td>86</td><td>74.7±8.3</td><td>19.0±0.1</td><td colspan="2">6.1±0.2</td><td>19.6±0.2</td></tr>
<tr><td>XIL5</td><td>0.8</td><td>0.2</td><td>39</td><td>266±8</td><td>12.0±0.2</td><td colspan="2">11.0±0.2</td><td>67±11</td></tr>
<tr><td>XIL6</td><td>0.7</td><td>0.2</td><td>11</td><td>208±34</td><td>48.1±3.2</td><td colspan="2">9.1±0.2</td><td>103±14</td></tr>
<tr><td>XIL7</td><td>1.0</td><td>0.8</td><td>54</td><td>178±4</td><td>50±15</td><td colspan="2">10.3±0.3</td><td>51±15</td></tr>
<tr><td>H1</td><td>0.4</td><td>0</td><td>0</td><td>819±43</td><td>6.4±0.3</td><td colspan="2">22.5±0.2</td><td>831±197</td></tr>
<tr><td>H2</td><td>0.6</td><td>0</td><td>0</td><td>974±43</td><td>7.5±0.4</td><td colspan="2">24.2±0.6</td><td>1144±18</td></tr>
<tr><td>XIH1</td><td>0.2</td><td>0.2</td><td>27</td><td>1070±64</td><td>662±94</td><td>27.2±2.1</td><td>24.9±0.5</td><td>673±104</td></tr>
<tr><td>XIH2</td><td>0.2</td><td>0.5</td><td>85</td><td>807±80</td><td>245±11</td><td>24.4±0.8</td><td>20.8±0.3</td><td>248±10</td></tr>
</table>

[a]Tensile testing conditions according to ISO 527-2, type 5B (melt pressed dogbone-shaped specimens with 35 × 6 × 1 mm, 2 mm×min^{-1} crosshead speed). [b]Keto and imino content with respect to ethylene repeat units of the polymer. Determined by ATR-IR spectra acquired on newly cut cross sectional areas of the specimens. [c]Determined by weighing the samples before and after extraction with hot toluene (6 h, 110 °C).

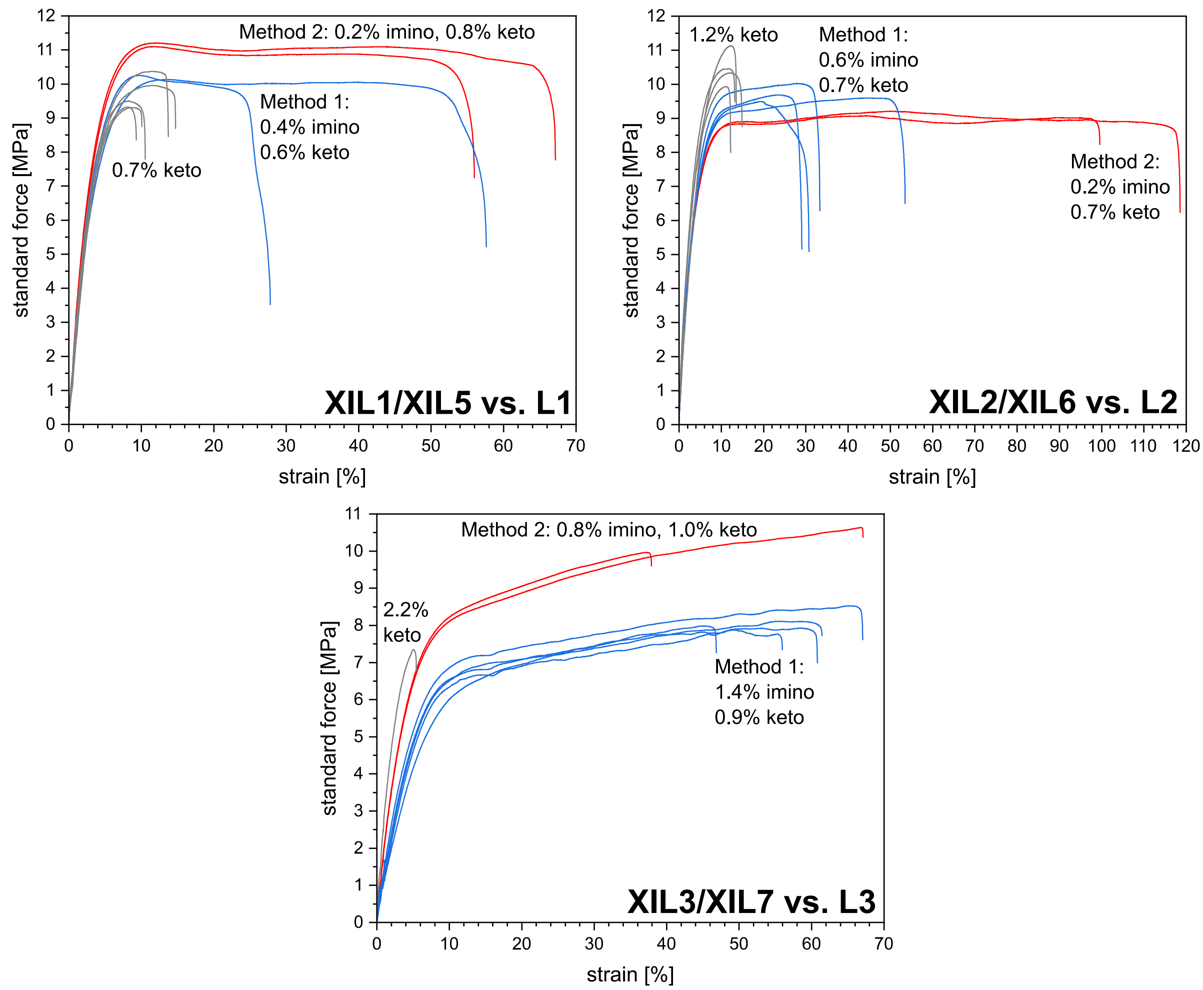

Figure S36. Stress-strain curves of imino-crosslinked LDPEs prepared by Method 1 or 2 and respective non-crosslinked keto-LDPE reference samples (melt pressed dogbone specimens, ISO 527-2, type 5B with 35 × 6 × 1 mm, 2 mm×min^{-1} crosshead speed).

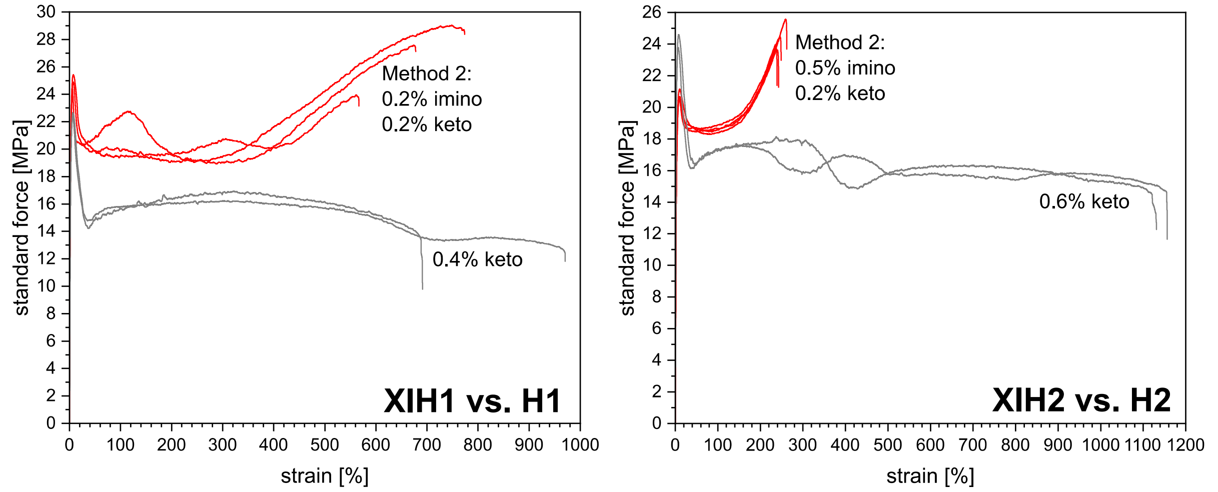

Figure S37. Stress-strain curves of imino-crosslinked HDPEs prepared by Method 2 and respective non-crosslinked keto-HDPE reference samples (melt pressed dogbone specimens, ISO 527-2, type 5B with 35 × 6 × 1 mm, 2 mm×min^{-1} crosshead speed).

2.6.1 Method 1

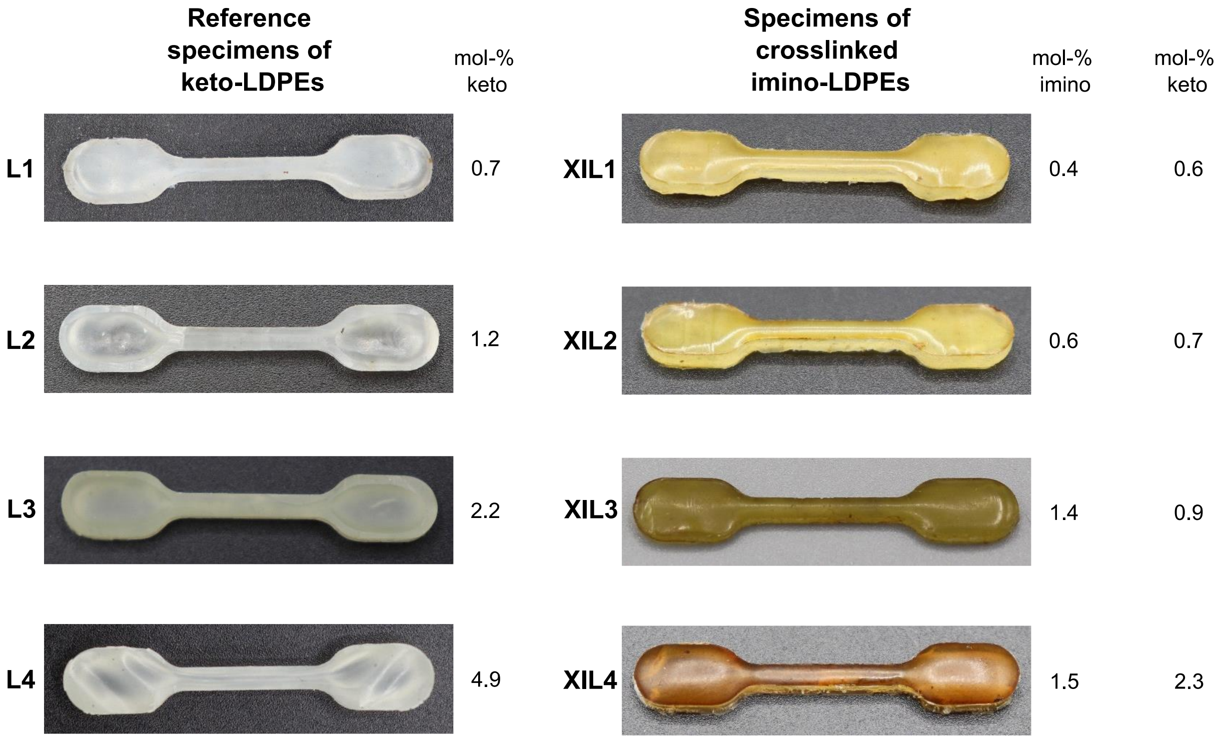

Figure S38. Exemplary photographs of keto-LDPE reference specimens L1 – L4 and specimens of imino-crosslinked LDPEs XIL1 – XIL4 prepared by Method 1 (*cf.* Table S5).

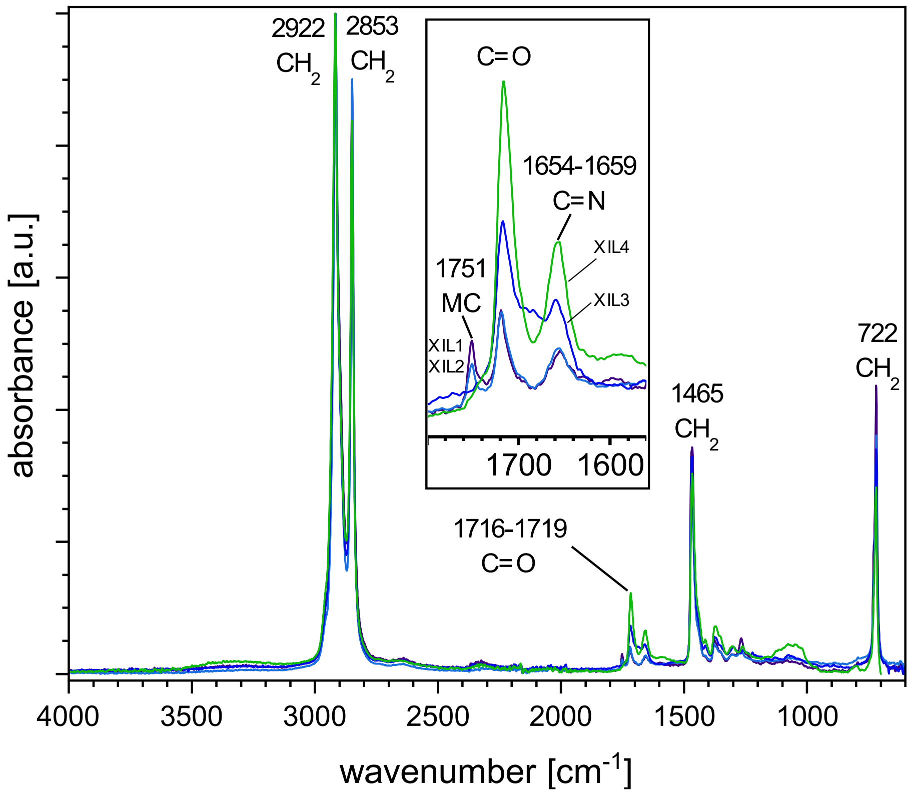

Figure S39. ATR-IR spectra of imino-crosslinked LDPE specimens XIL1 – XIL4 prepared by Method 1 (*cf.* Table S5). Spectra are normalized to the maximum at 2922 cm^{-1}.

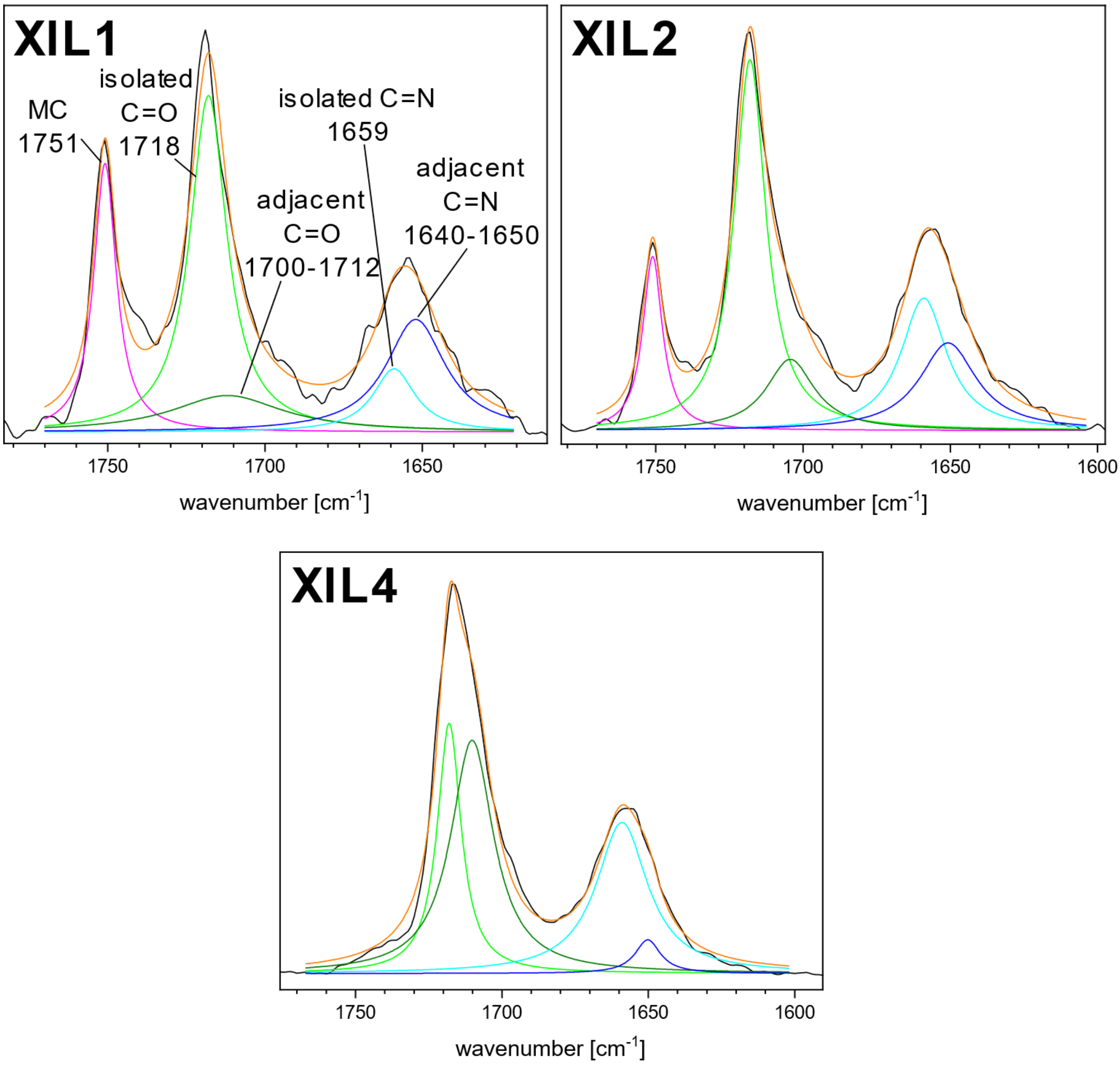

Figure S40. Exemplary ATR-IR deconvolution of bands evoked by isolated imino groups (1659 cm^{-1}), imino groups in close proximity to ketones/imines (1640 – 1650 cm^{-1}), isolated keto groups (1718 cm^{-1}), keto groups in close proximity to ketones/imines (1700 – 1712 cm^{-1}) and methyl carbonate end groups (MC, 1751 cm^{-1}) with five Lorentzian functions for imino-crosslinked LDPE specimens XIL1, XIL2, XIL4 prepared by Method 1 (*cf.* Table S5). Carbonate groups are incorporated into the polymer by chain transfer reactions to the solvent dimethyl carbonate during synthesis of the initial keto-LDPEs.

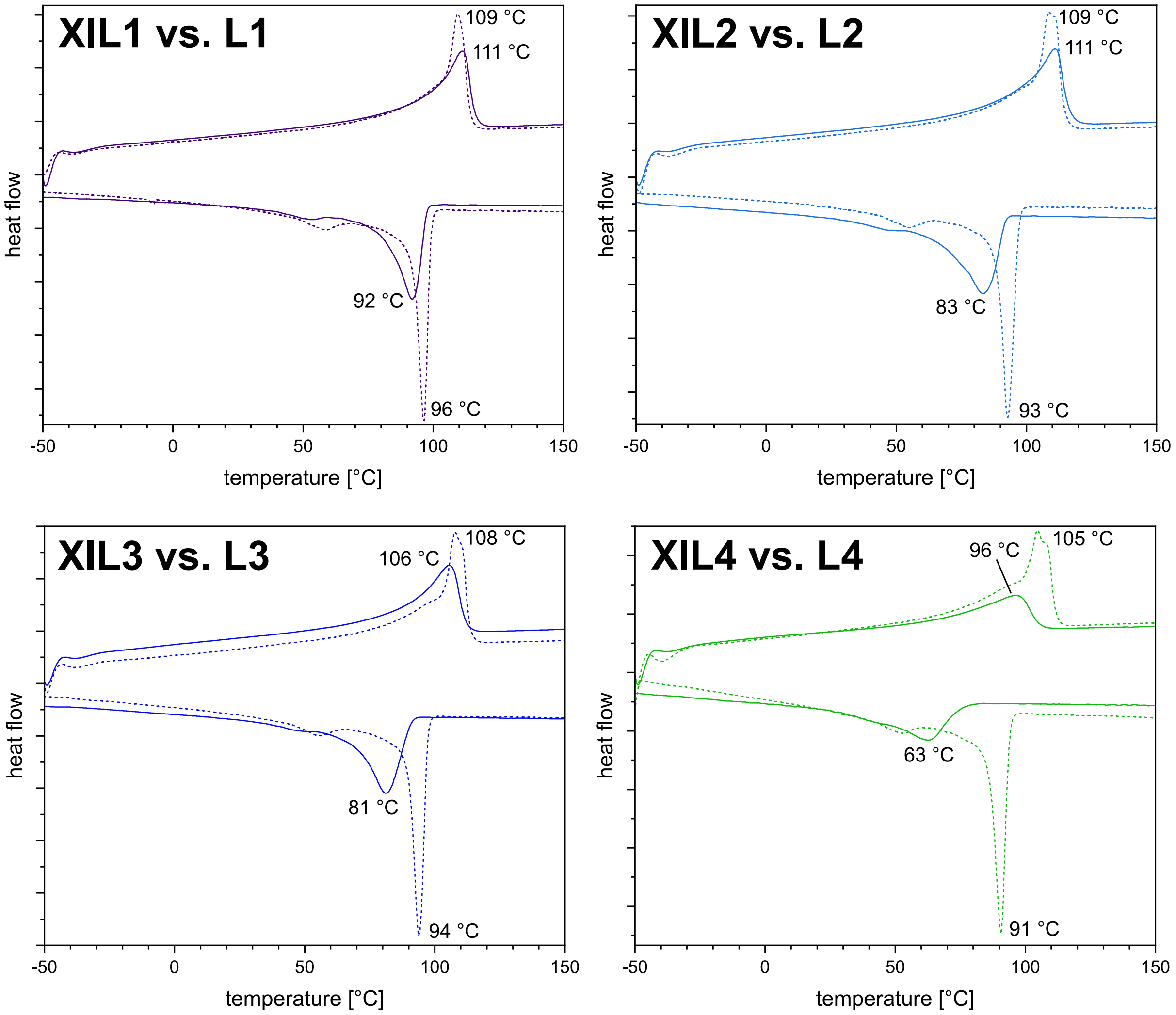

Figure S41. Comparative 2nd heating and 1st cooling DSC curves of imino-crosslinked LDPEs XIL1 – XIL4 and respective initial keto-LDPEs L1 – L4 of Table S5.

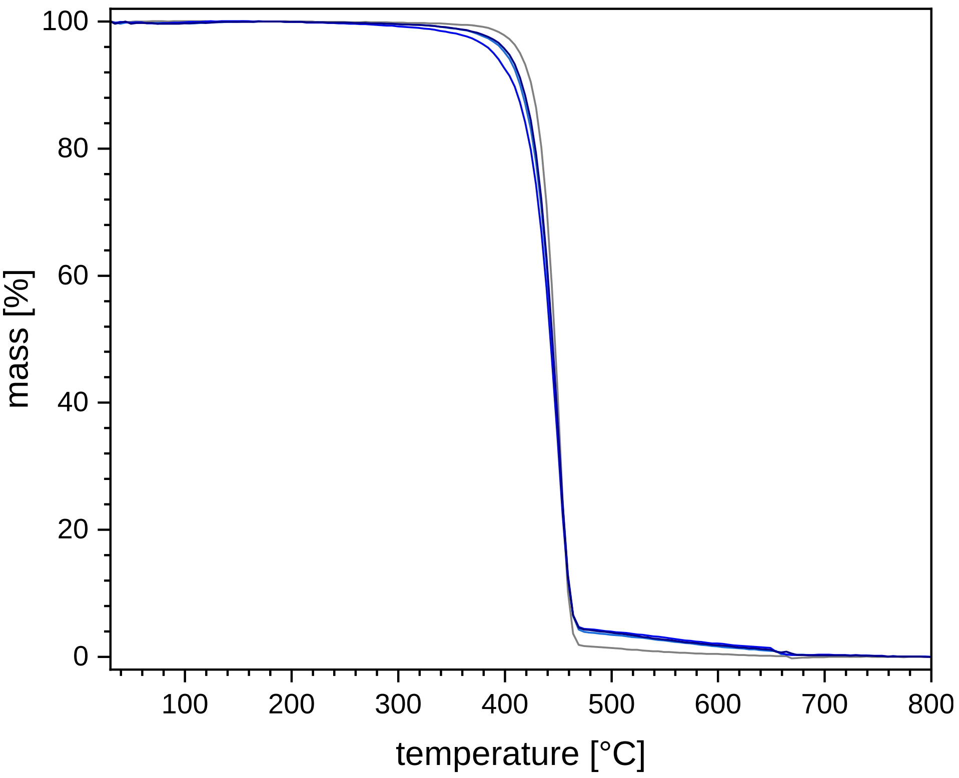

Figure S42. Comparative thermogravimetric analysis (TGA) of commercial LDPE and imino-crosslinked LDPEs XIL1 – XIL3 of Table S5.

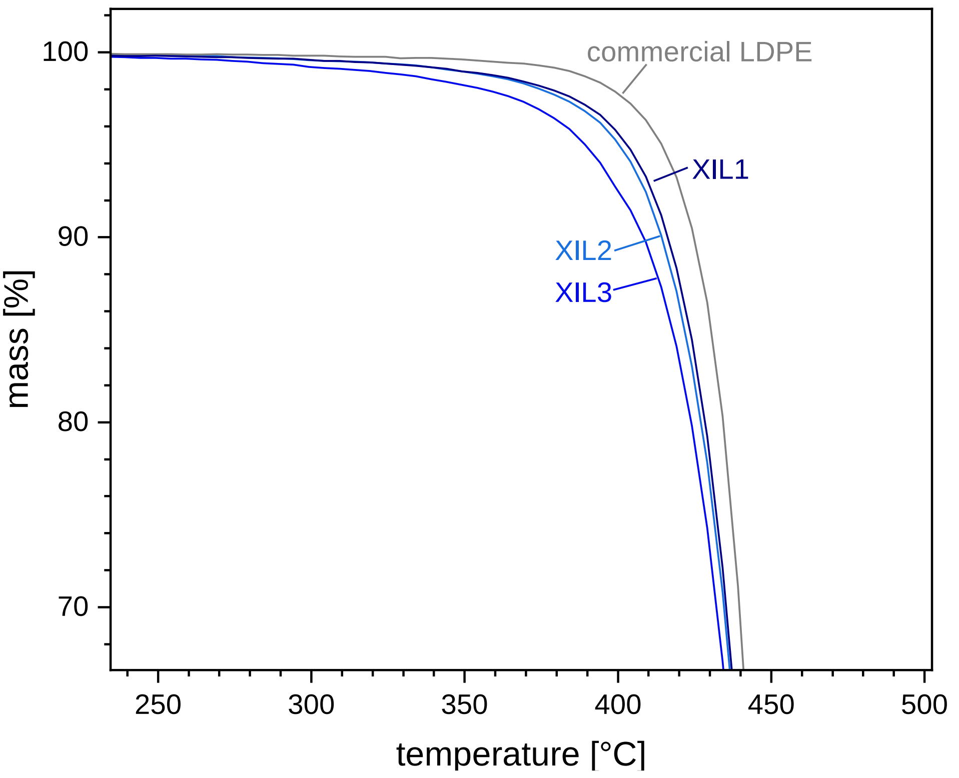

Figure S43. Comparative thermogravimetric analysis (TGA) of commercial LDPE and imino-crosslinked LDPEs XIL1 – XIL3 in the temperature range of 250 – 500 °C.

2.6.2 Method 2

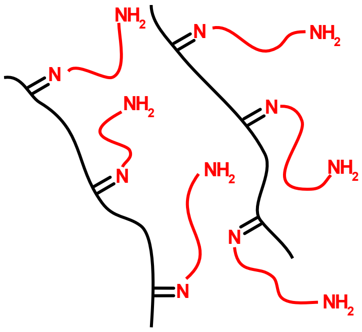

Table S7. Properties of the intermediate amino-functionalized PEs synthesized from keto-PEs and large excess of 1,12-diaminododecane. Initial keto-LDPEs L1 – L3 and keto-HDPEs H1, H2 are given as reference.[a]

#	initial keto-PE (keto mol-%)	χ(C=O) [mol-%][a]	T_m [°C] (% cryst.)[b]	T_c [°C] (% cryst.)[c]
L1	-	0.7	109 (44)	96 (48)
L2	-	1.2	109 (37)	93 (40)
L3	-	2.2	108 (33)	94 (40)
H1	-	0.4	134 (68)	112 (67)
H2	-	0.6	135 (67)	112 (67)
AIL1	L1 (0.7)	0.06[d]	110 (37)	91 (39)
AIL2	L2 (1.2)	0.09[d]	109 (38)	87 (38)
AIL3	L3 (2.2)	0.02[d]	103 (28)	74 (29)
AIH1	H1 (0.4)	<0.01[d]	134 (57)	109 (57)
AIH2	H2 (0.6)	<0.01[d]	133 (50)	107 (51)

[a]Reaction conditions: 500 mg of keto-PE, 10 – 15 equiv. of 1,12-diaminododecane with respect to keto groups, 160 °C, 6 h under N_2, 1 h at 10^{-2} mbar (for further details on condensation conditions *cf.* Section 1.2.4). [a]Keto content with respect to ethylene repeat units of the polymer. Determined by ATR-IR spectra. [b]Peak melting point and degree of crystallinity determined by DSC, 2nd heating cycle (10 K×min^{-1}). [c]Peak crystallization point and degree of crystallinity determined by DSC, 1st cooling cycle (10 K×min^{-1}). [d]Quantification of imino content from ATR-IR not possible due to a considerable number of amino groups in the sample. Keto content was estimated from the ratio of the C=O band intensity vs. the CH_2 band intensity (2922 cm^{-1}) and the method of Figure S1.

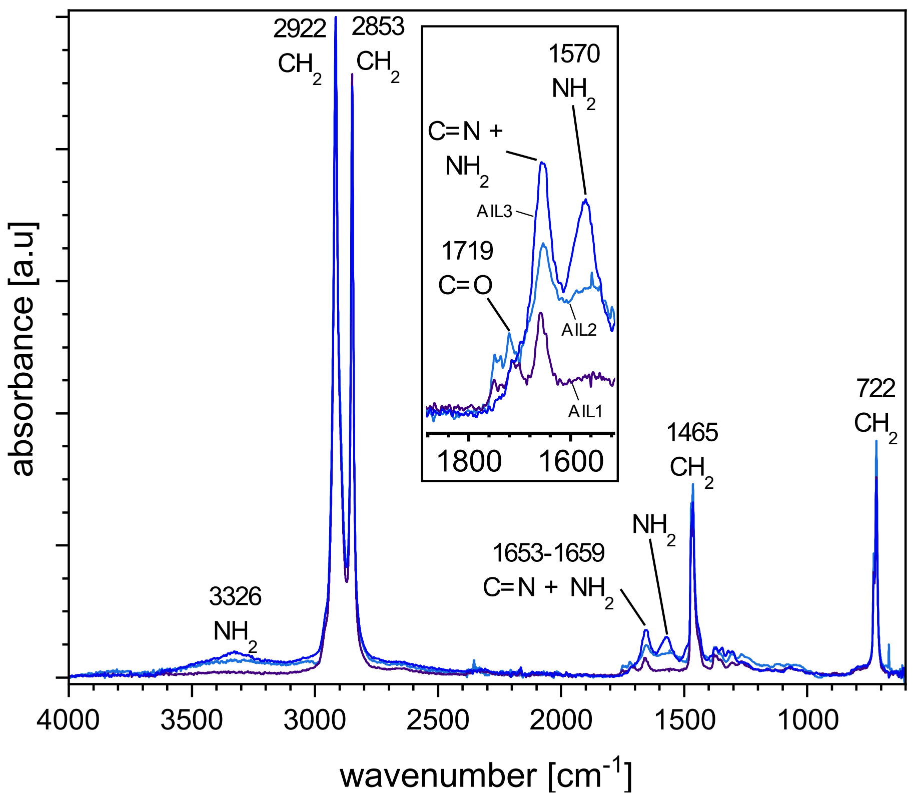

Figure S44. ATR-IR spectra of amino-functionalized LDPEs AIL1 – AIL3 (*cf.* Table S7). Spectra are normalized to the maximum at 2922 cm^{-1}.

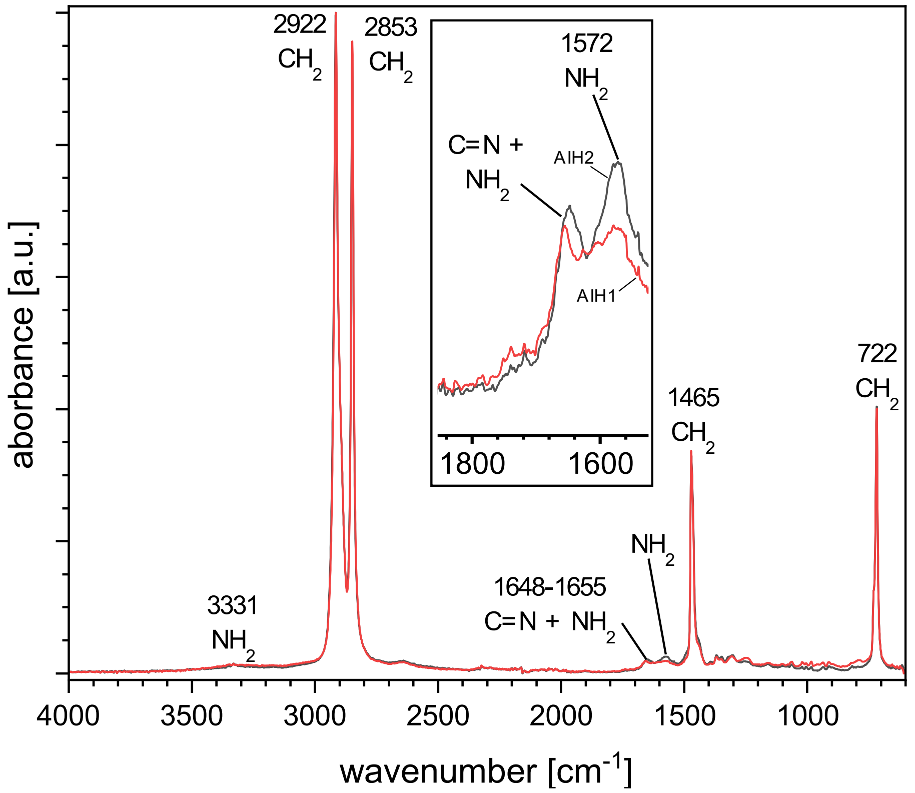

Figure S45. ATR-IR spectra of amino-functionalized HDPEs AIH1, AIH3 (*cf.* Table S7). Spectra are normalized to the maximum at 2922 cm^{-1}.

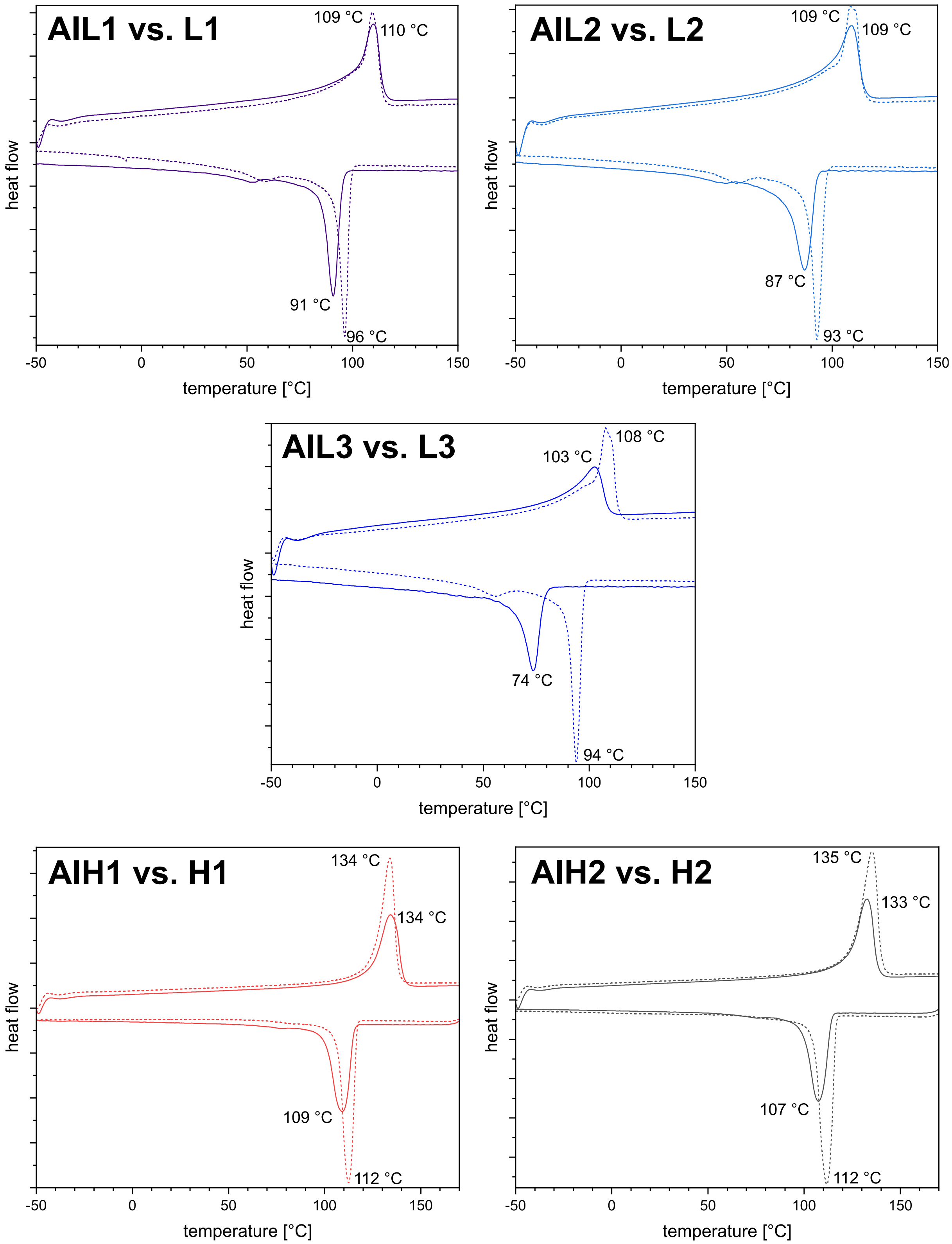

Figure S46. Comparison of 2nd heating and 1st cooling DSC curves of amino-functionalized LDPEs AIL1 – AIL3 and -HDPEs AIH1, AIH2 with respective initial keto-LDPEs L1 – L3 and -HDPEs H1, H2 of Table S7.

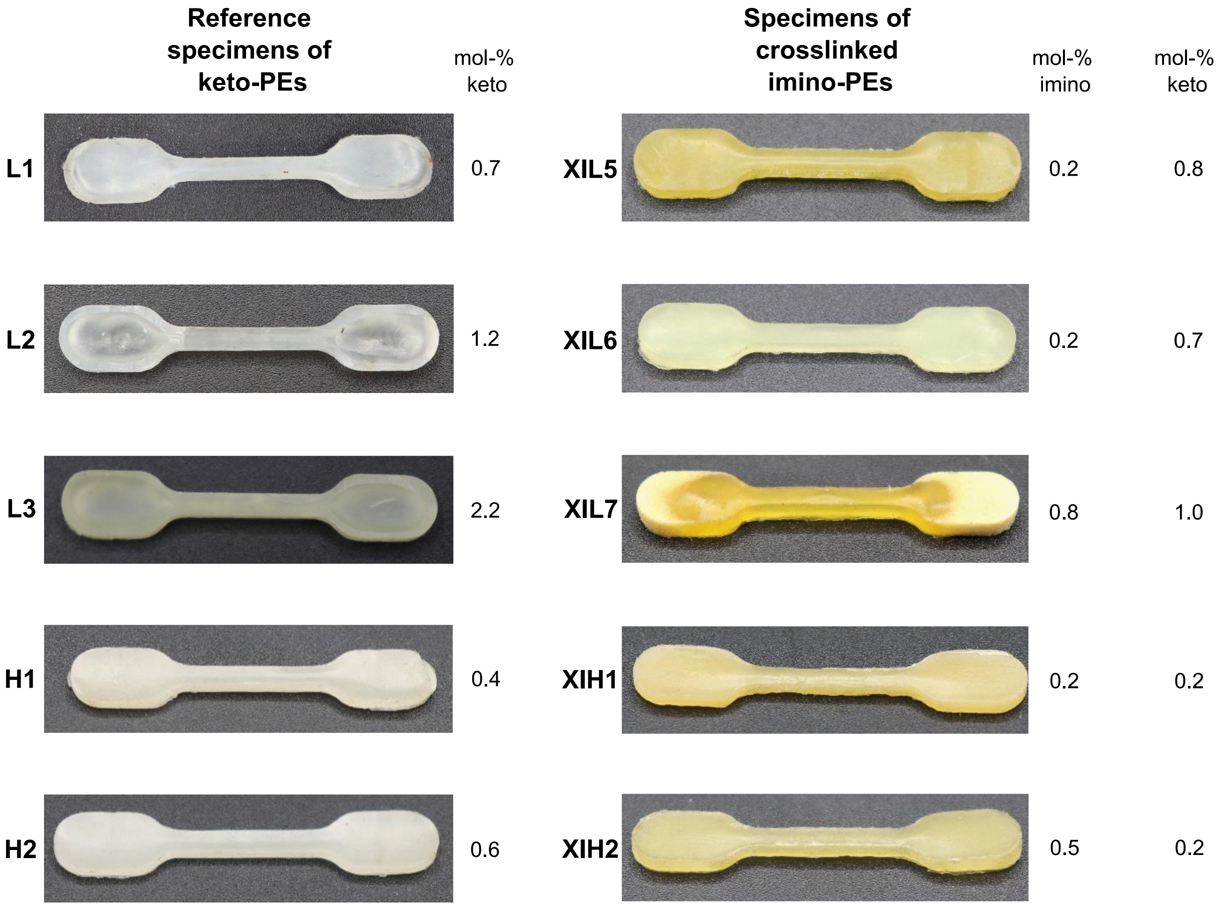

Figure S47. Exemplary photographs of keto-PE reference specimens L1 – L3, H1, H2 and specimens of imino-crosslinked PEs XIL5 – XIL7, XIH1, XIH2 prepared by Method 2 (*cf.* Table S5).

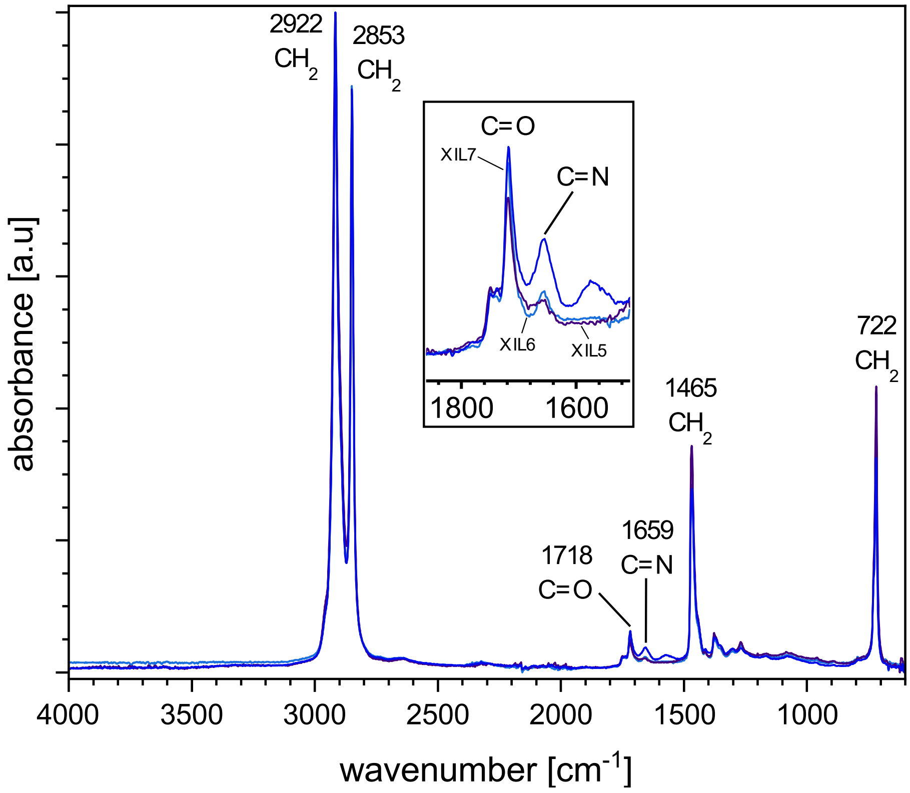

Figure S48. ATR-IR spectra of imino-crosslinked LDPE specimens XIL5 – XIL7 prepared by Method 2 (*cf.* Table S5). Spectra are normalized to the maximum at 2922 cm^{-1}.

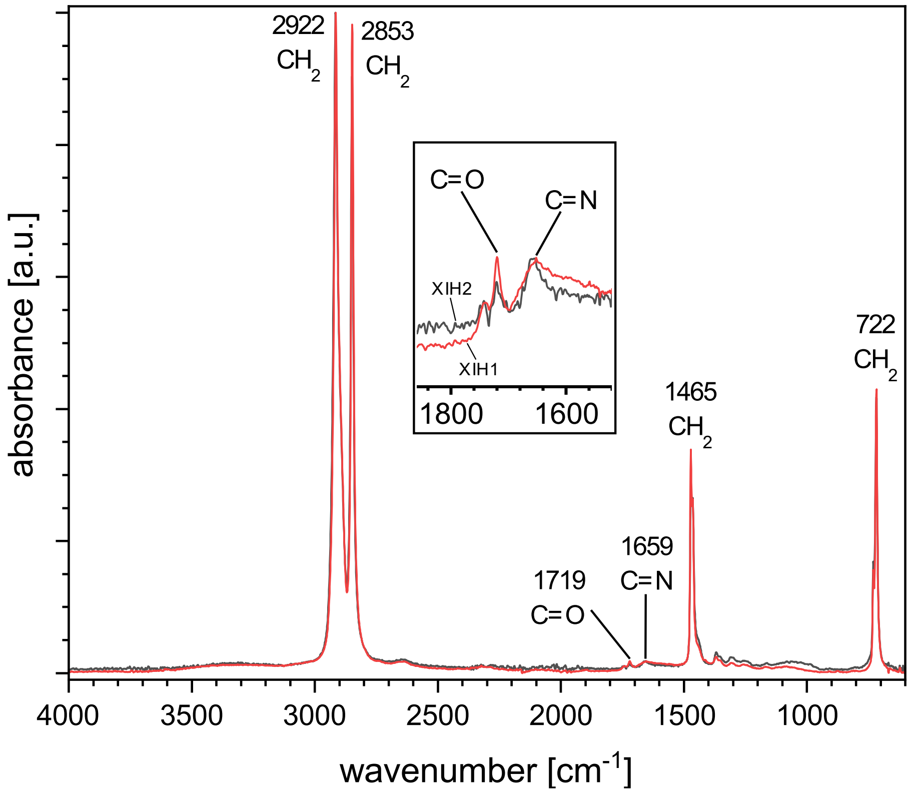

Figure S49. ATR-IR spectra of imino-crosslinked HDPE specimens XIH1, XIH2 prepared by Method 2 (*cf.* Table S5). Spectra are normalized to the maximum at 2922 cm^{-1}.

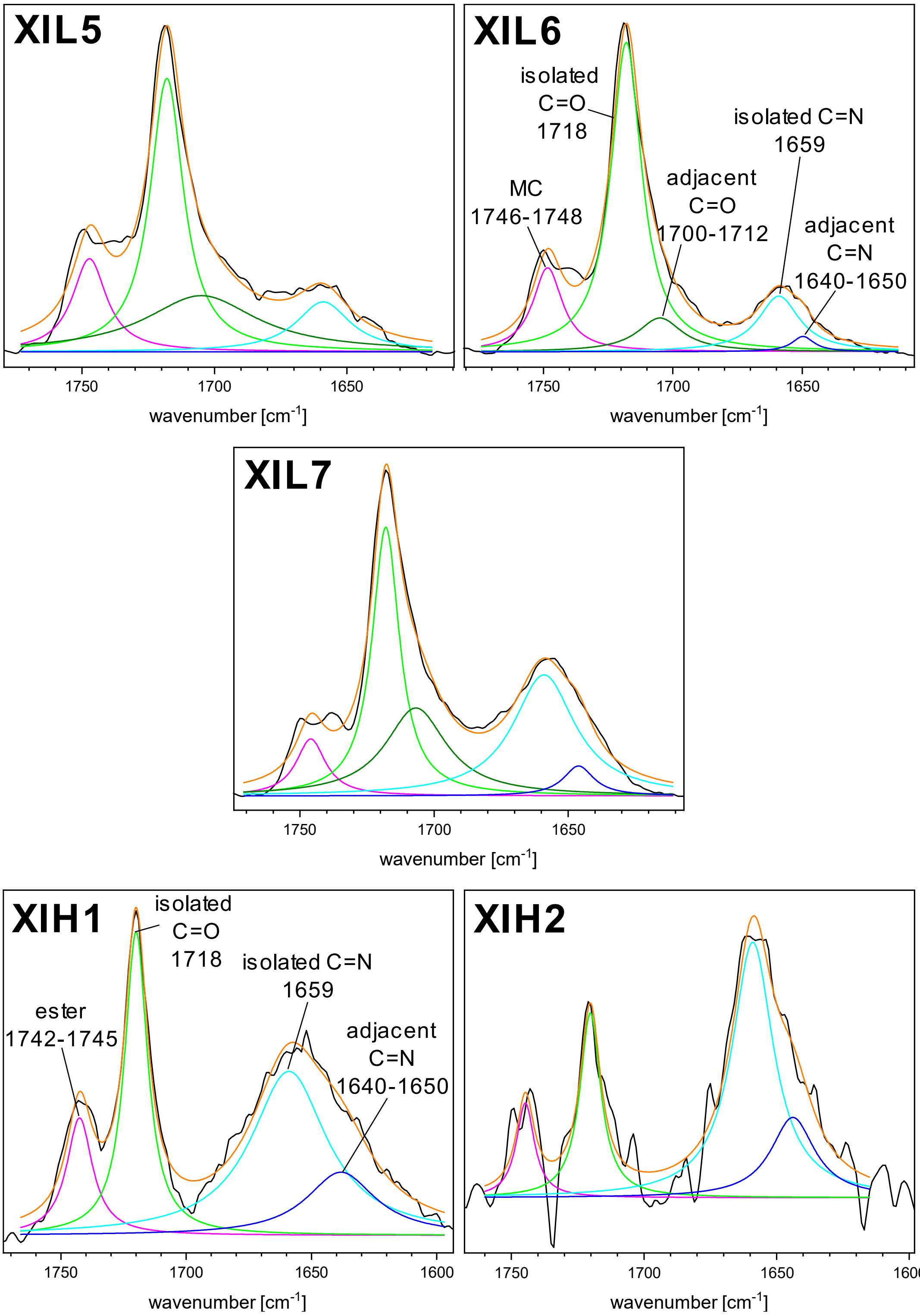

Figure S50. ATR-IR deconvolution of bands evoked by isolated imino groups (1659 cm^{-1}), imino groups in close proximity to ketones/imines (1640 – 1650 cm^{-1}), isolated keto groups (1718 cm^{-1}), keto groups in close proximity to ketones/imines (1700 – 1712 cm^{-1}) and methyl carbonate (MC) / esters groups with five Lorentzian functions for imino-crosslinked PE specimens prepared by Method 2 (*cf.* Table S5). Carbonate groups are incorporated into the polymer by chain transfer reactions to the solvent dimethyl carbonate during synthesis of the initial keto-LDPEs. Aliphatic esters likely stem from oxidation by trace impurities of air oxygen during prolonged heating needed for crosslinking of keto-HDPEs.

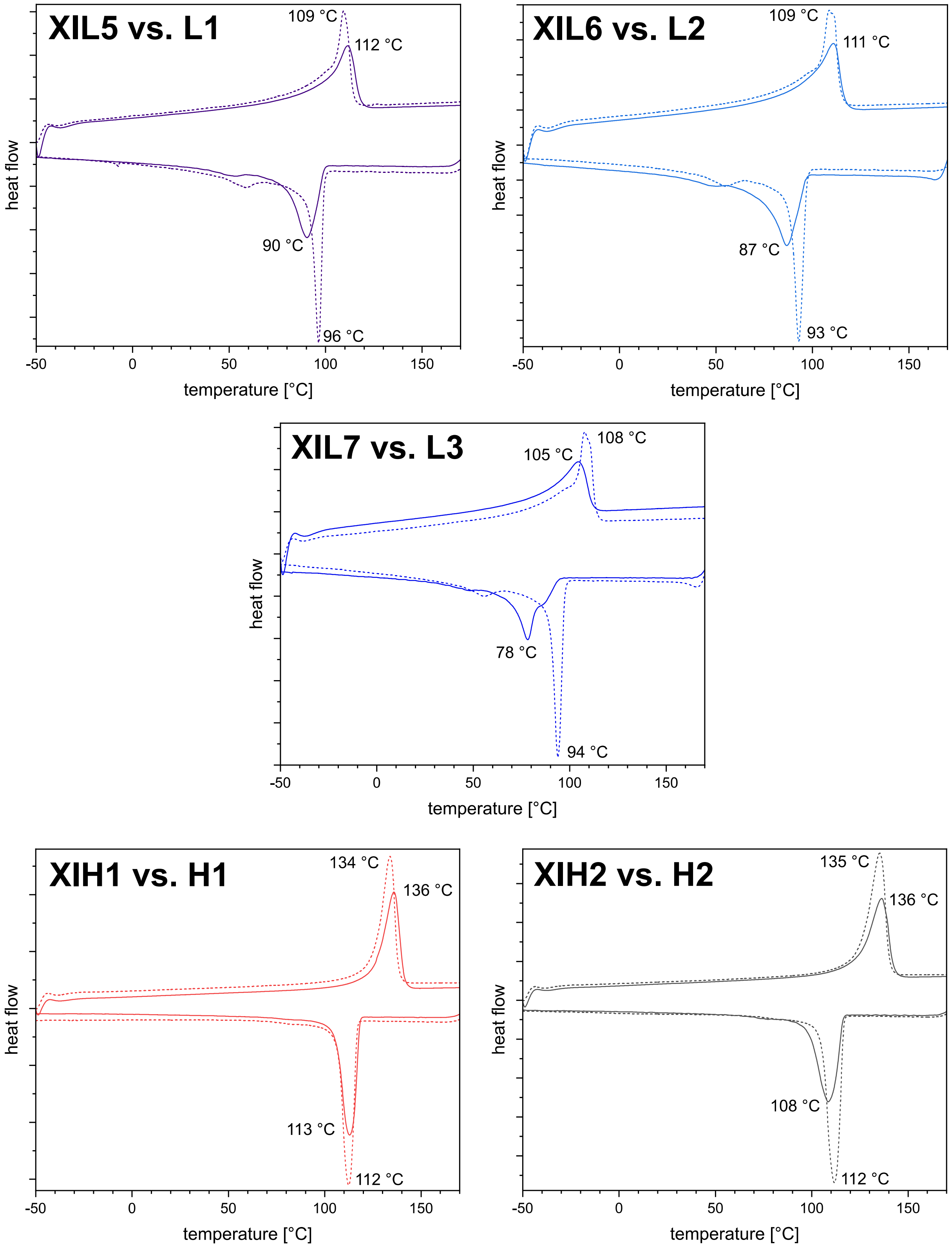

Figure S51. Comparison of 2nd heating and 1st cooling DSC curves of imino-crosslinked LDPEs XIL5 – XIL7 and HDPEs XIH1, XIH2 with respective initial keto-LDPEs L1 – L3 and -HDPEs H1, H2 of Table S5.

2.7 Hydrolysis

Table S8. Data on recycled keto-PEs after acidic hydrolysis of imino-crosslinked PEs and comparison with properties of initial keto-PEs.[a]

#	yield [%][b]	gel fraction before hyd. [wt.-%][c]	gel fraction after hyd. [wt.-%][c]	M_n initial [10^3 g×mol^{-1}] (M_w/M_n)[d]	M_n after hyd. [10^3 g×mol^{-1}] (M_w/M_n)[d]	T_m initial [°C] (% cryst.)[e]	T_m after hyd. [°C] (% cryst.)[e]	χ(CO) initial [mol-%][f]	χ(CO) after hyd. [mol-%][f]
L1H	96	39	0.7	14.4 (1.5)	8.3 (2.2)[g]	109 (44)	110 (38)	0.7 (0.6)	0.7 (1.0)
L2H	92	11	0.4	12.3 (1.7)	10.9 (1.6)[g]	109 (37)	110 (38)	1.2 (1.3)	1.3 (1.2)
L3H	95	66	9.8	12.1 (1.4)	11.6 (1.5)[g]	108 (33)	109 (36)	2.2 (2.0)	2.1 (2.2)
L3H'	93	54	6.8	12.1 (1.4)	5.9 (2.3)[g]	108 (33)	107 (36)	2.2 (2.0)	2.0 (2.2)
H1H	97	27	<0.1	53.9 (1.7)	43.9 (1.5)[g]	134 (68)	140 (61)	0.4 (0.4)	0.3 (0.4)
H2H	92	85	3.1	51.9 (1.8)	48.2 (1.5)[g]	135 (67)	139 (58)	0.6 (0.6)	0.5 (0.6)

[a]Hydrolysis conditions: 800 mg of imino-crosslinked LDPE or 400 mg of imino-crosslinked HDPE, 240 mg of *p*-TsOH×H_2O, 100 mL of 1,4-dioxane (LDPEs) or 100 mL of toluene (HDPEs), 1.5 mL of water, 140 °C, 72 h, 500 rpm. [b]Mass of recycled polymer after hydrolysis vs. initial mass of imino-crosslinked PE. [c]Comparison of gel fraction before and after hydrolysis. Determined by weighing the samples before and after extraction with hot toluene (6 h, 110 °C). [d]Comparison of average molecular weight and polydispersity of initial and recycled keto-PEs. Determined by GPC in 1,2-dichlorobenzene at 160 °C via linear calibration against PE standards. [e]Comparison of peak melting point and degree of crystallinity of initial and recycled keto-PEs. Determined by DSC, 2nd heating cycle (10 K×min^{-1}). [f]Comparison of keto content with respect to ethylene repeat units determined by ^{1}H NMR (and by ATR-IR, *cf.* Figure S1) of initial and recycled keto-PEs. [g]Sample contains minor amounts of high-molecular-weight fractions with >10^6 g×mol^{-1}.

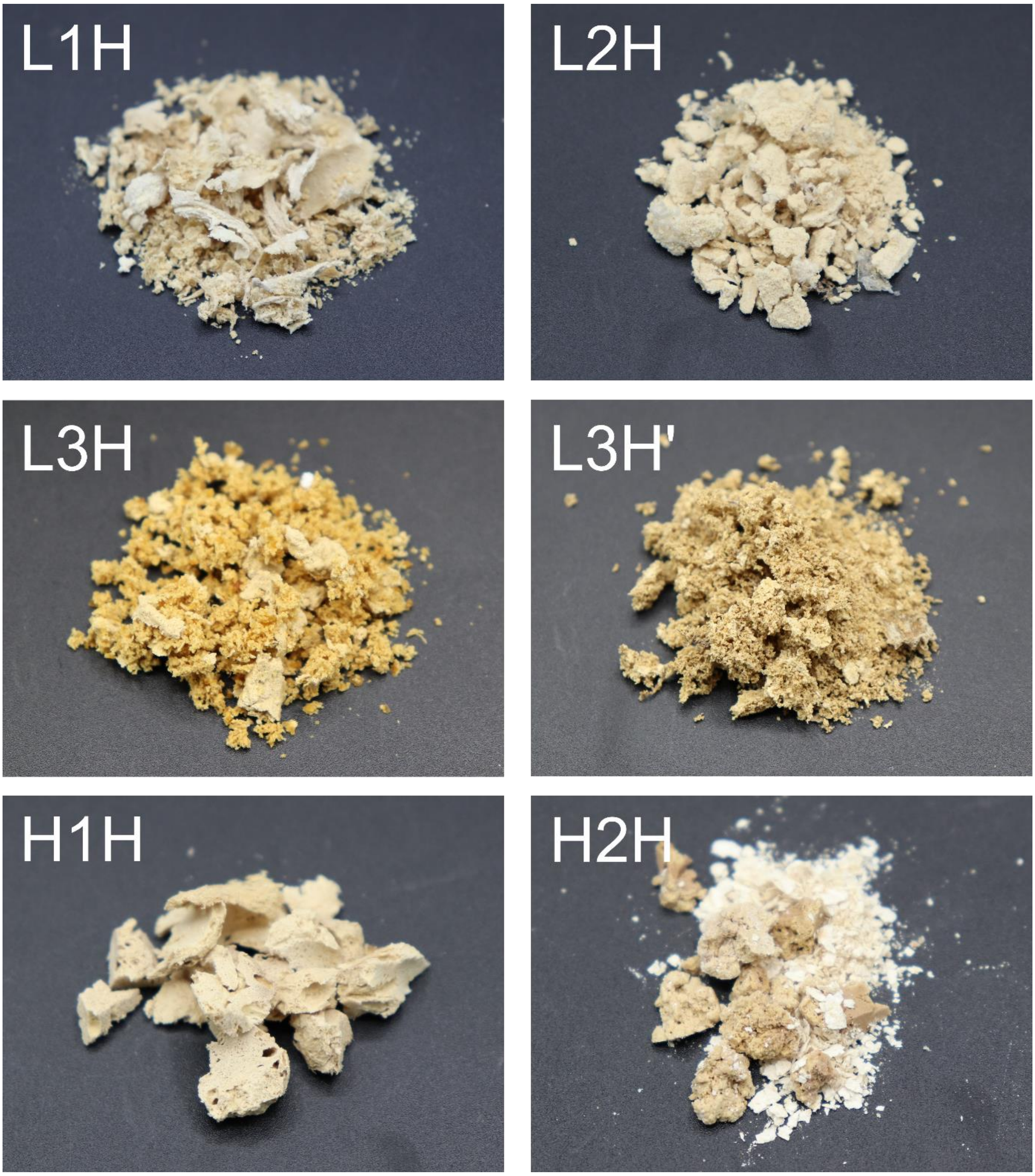

Figure S52. Photographs of recycled keto-LDPEs L1H – L3H, L3H' and -HDPEs H1H, H2H from acidic hydrolysis of imino-crosslinked PEs (*cf.* Table S8).

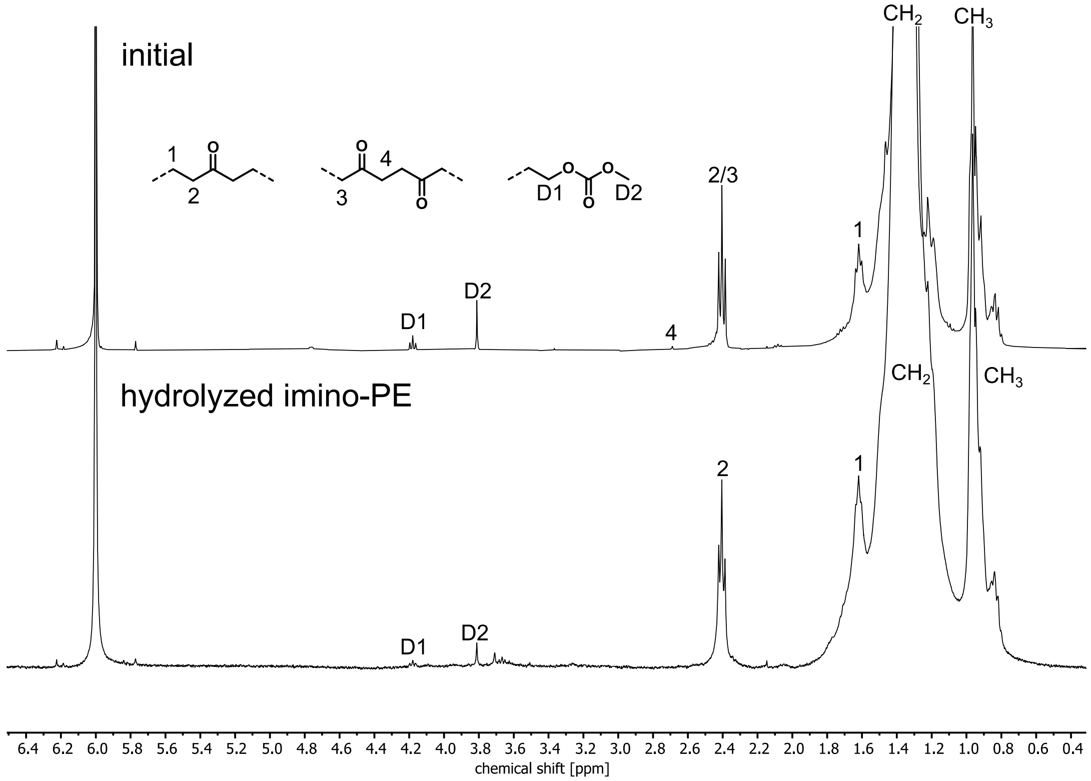

Figure S53. Comparative ^{1}H NMR spectra ($C_2D_2Cl_4$, 373 K) of initial keto-LDPE L1 (0.7 mol-% keto groups) and recycled keto-LDPE L1H (0.7 mol-% keto groups) from acidic hydrolysis of imino-crosslinked LDPE XIL5 (*cf.* Table S5 and S8).

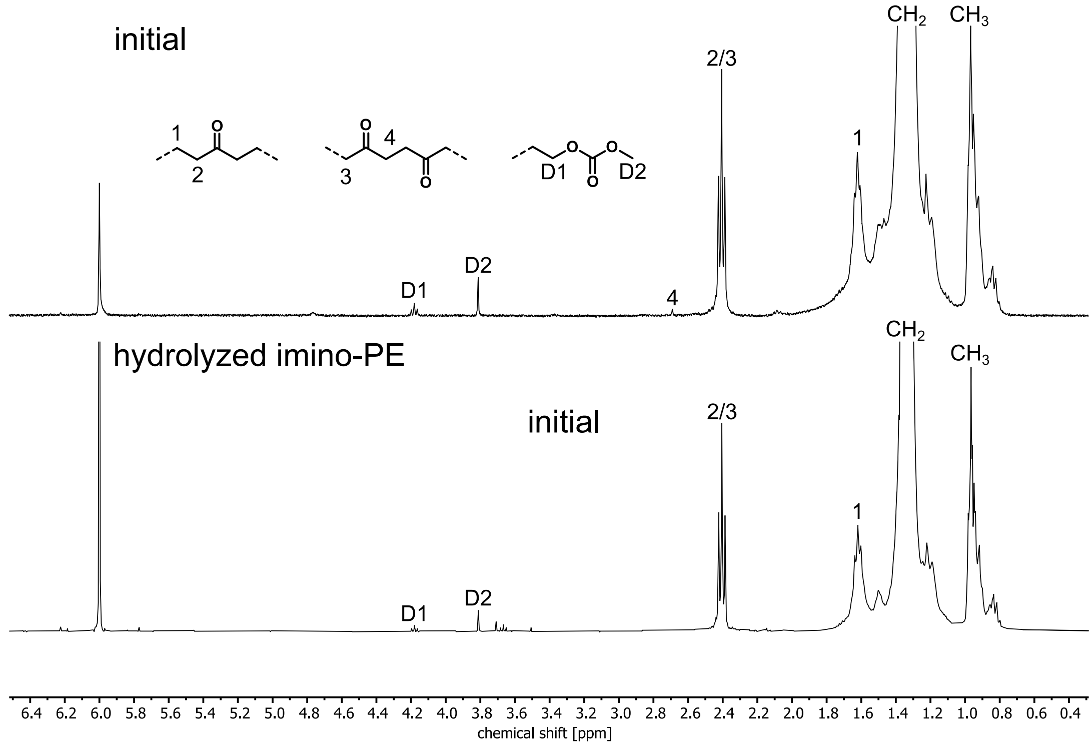

Figure S54. Comparative ^{1}H NMR spectra ($C_2D_2Cl_4$, 373 K) of initial keto-LDPE L2 (1.2 mol-% keto groups) and recycled keto-LDPE L2H (1.3 mol-% keto groups) from acidic hydrolysis of imino-crosslinked LDPE XIL6 (*cf.* Table S5 and S8).

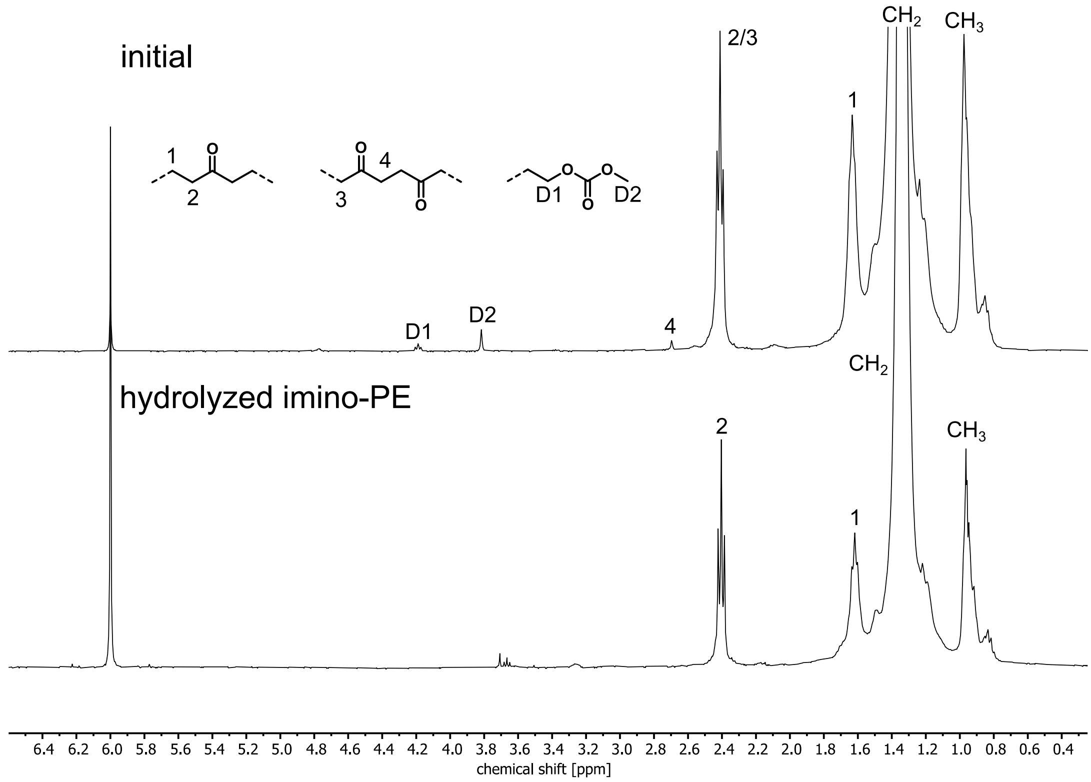

Figure S55. Comparative ^{1}H NMR spectra ($C_2D_2Cl_4$, 373 K) of initial keto-LDPE L3 (2.2 mol-% keto groups) and recycled keto-LDPE L3H (2.1 mol-% keto groups) from acidic hydrolysis of imino-crosslinked LDPE XIL3 (*cf.* Table S5 and S8).

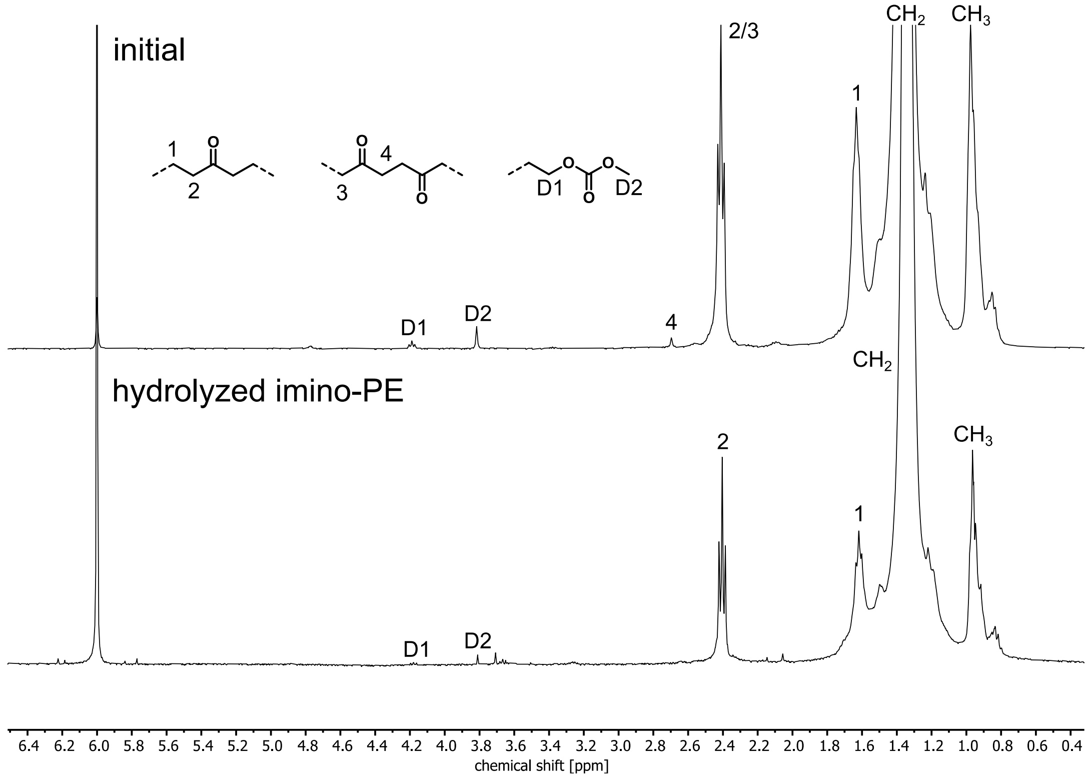

Figure S56. Comparative ^{1}H NMR spectra ($C_2D_2Cl_4$, 373 K) of initial keto-LDPE L3 (2.2 mol-% keto groups) and recycled keto-LDPE L3H' (2.2 mol-% keto groups) from acidic hydrolysis of imino-crosslinked LDPE XIL7 (*cf.* Table S5 and S8).

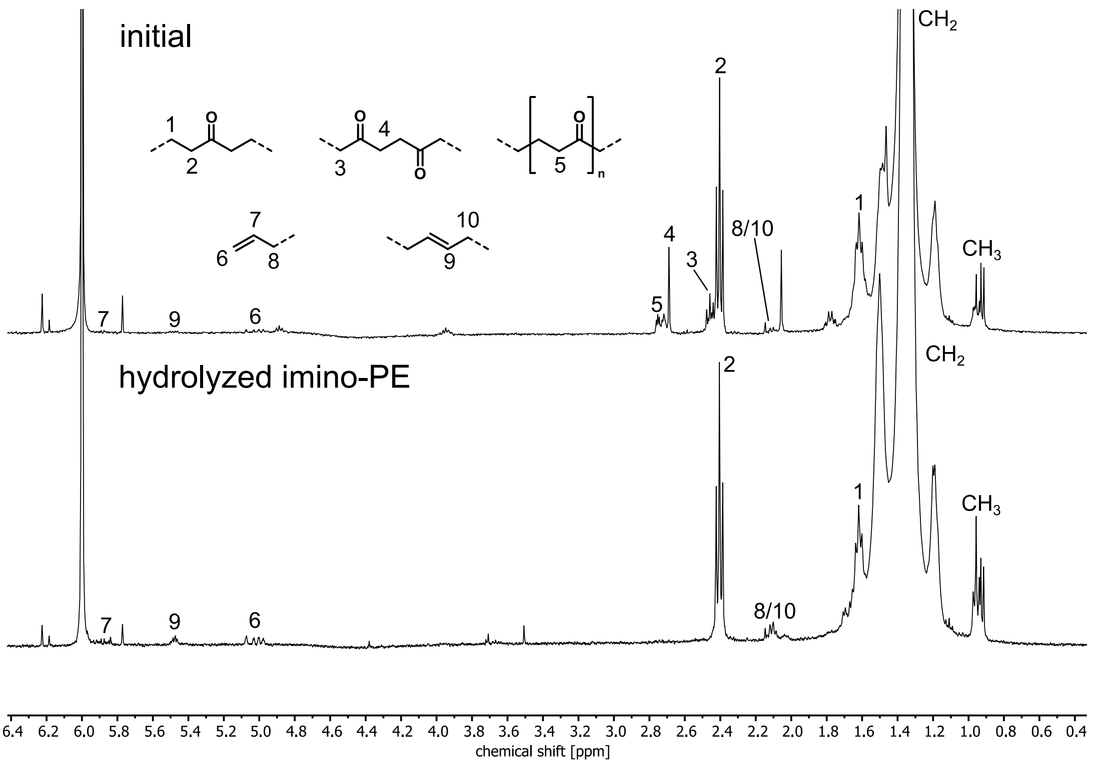

Figure S57. Comparative ^{1}H NMR spectra ($C_2D_2Cl_4$, 373 K) of initial keto-HDPE H2 (0.6 mol-% keto groups) and recycled keto-HDPE H2H (0.5 mol-% keto groups) from acidic hydrolysis of imino-crosslinked HDPE XIH2 (*cf.* Table S5 and S8).

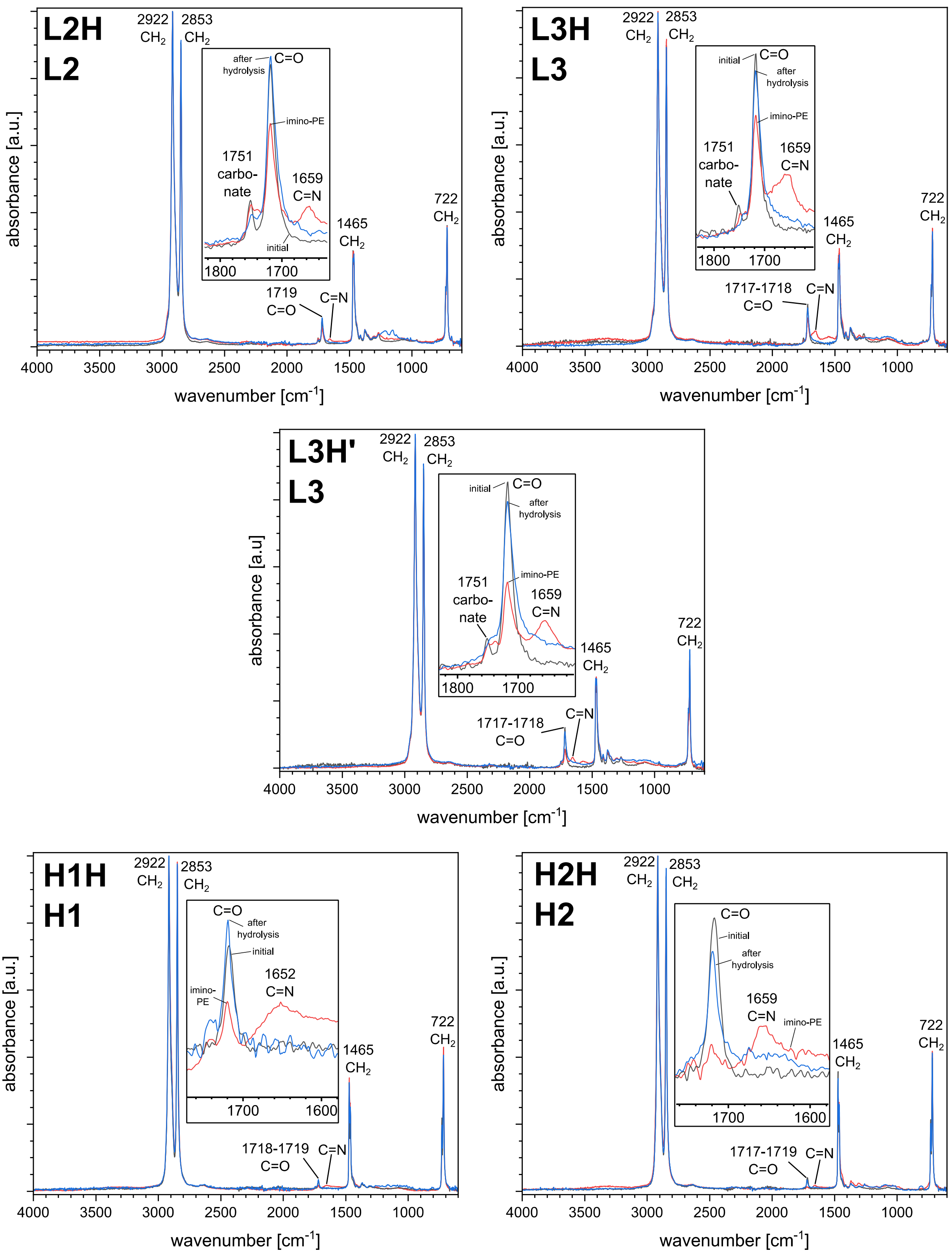

Figure S58. Comparative ATR-IR spectra of initial keto-PEs, imino-crosslinked PEs and recycled keto-PEs after acidic hydrolysis (*cf*. Table S8). Spectra are normalized to the maximum at 2922 cm^{-1}.

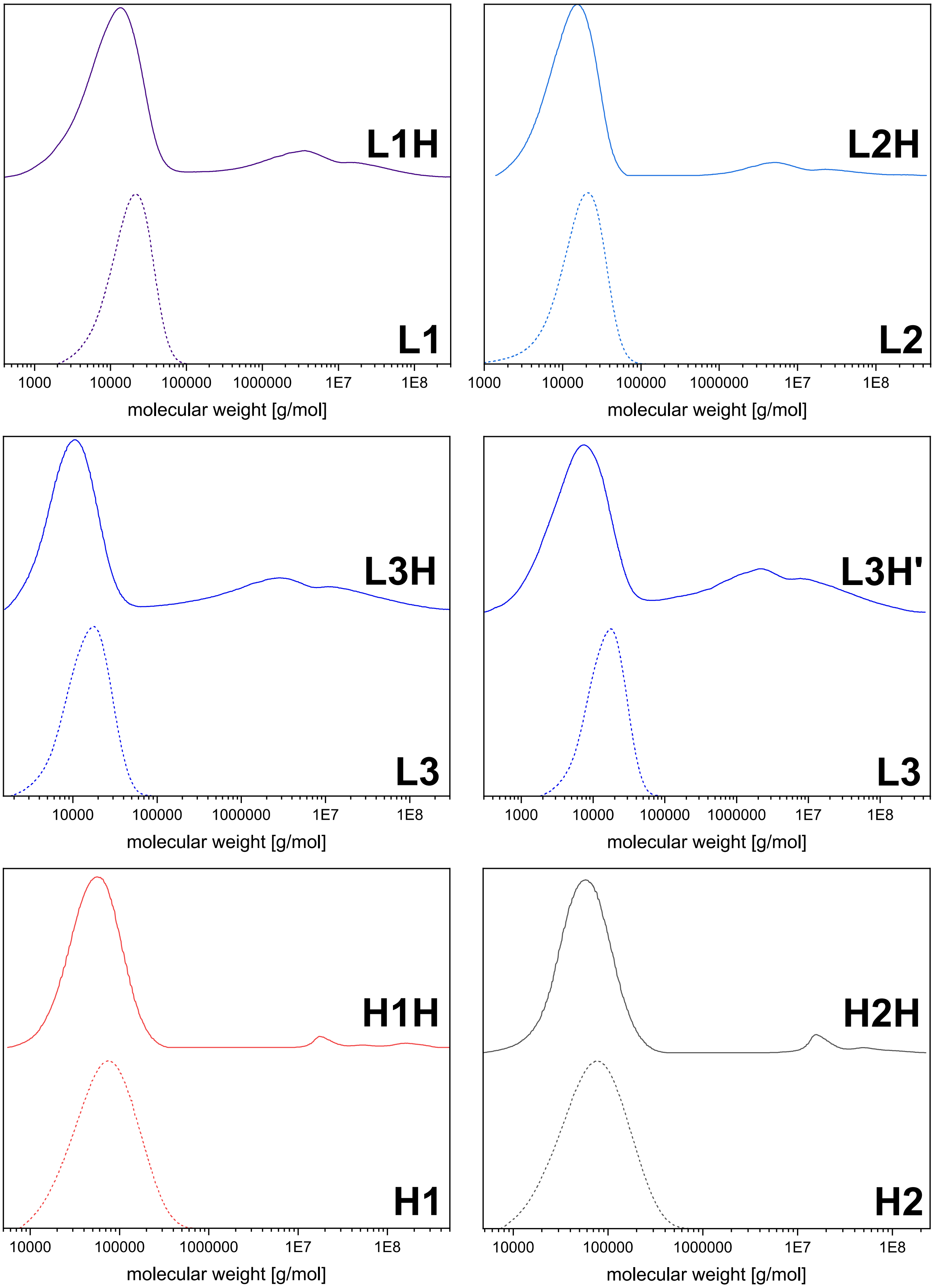

Figure S59. Comparative GPC molecular weight distributions of initial keto-PEs and recycled keto-PEs after acidic hydrolysis (*cf.* Table S8).

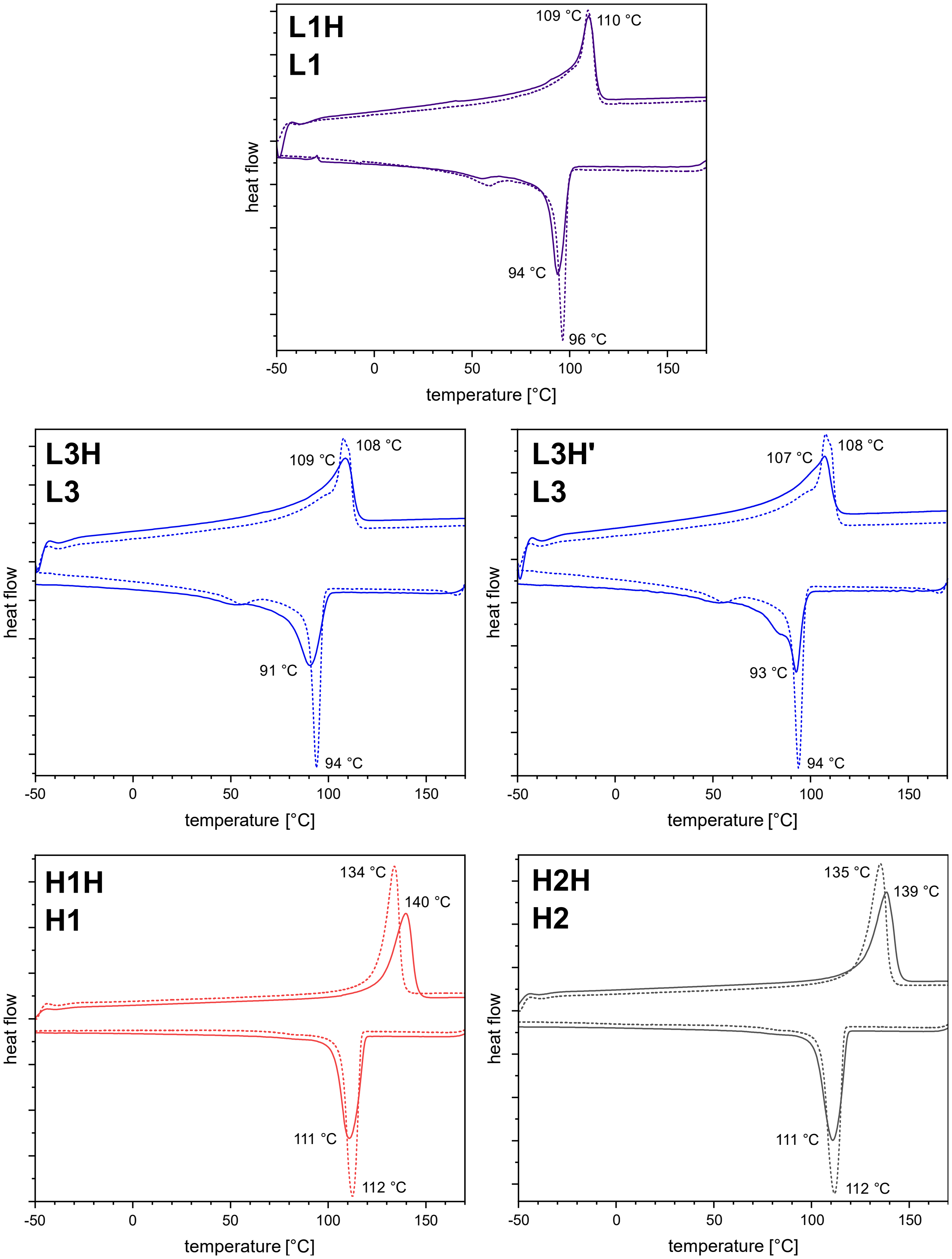

Figure S60. Comparison of 2nd heating and 1st cooling DSC curves of initial keto-PEs and recycled keto-PEs after hydrolysis (*cf.* Table S8).

2.8 Stability under Ambient Conditions

2.8.1 Stability under Air

To investigate the persistence of imino-crosslinked LDPEs at room temperature under air, tensile test specimens (ISO 527-2, type 5B) were prepared by Method 1 as already described (*cf*, Section 1.2.4). The samples had an initial keto content of 0.9 mol-% and an imino content of 0.7 mol-% (*cf*. Figure S61, A). The initial gel fraction was 34 ± 8 wt.-% as determined from 5 different samples (*cf*. Section 1.2.5). The mechanical properties of half of the specimens were investigated directly after melt pressing, whereas the other half was exposed to air for 3 months (20 °C, 40 % relative humidity). After this time, both the functional group density in the bulk material (0.9 mol-% keto and 0.8 mol-% imino content) and the gel fraction (33 ± 15 wt.-%) were virtually unaltered. Additionally, mechanical properties remained on a par with initial specimens (*cf*. Figure S61, B).

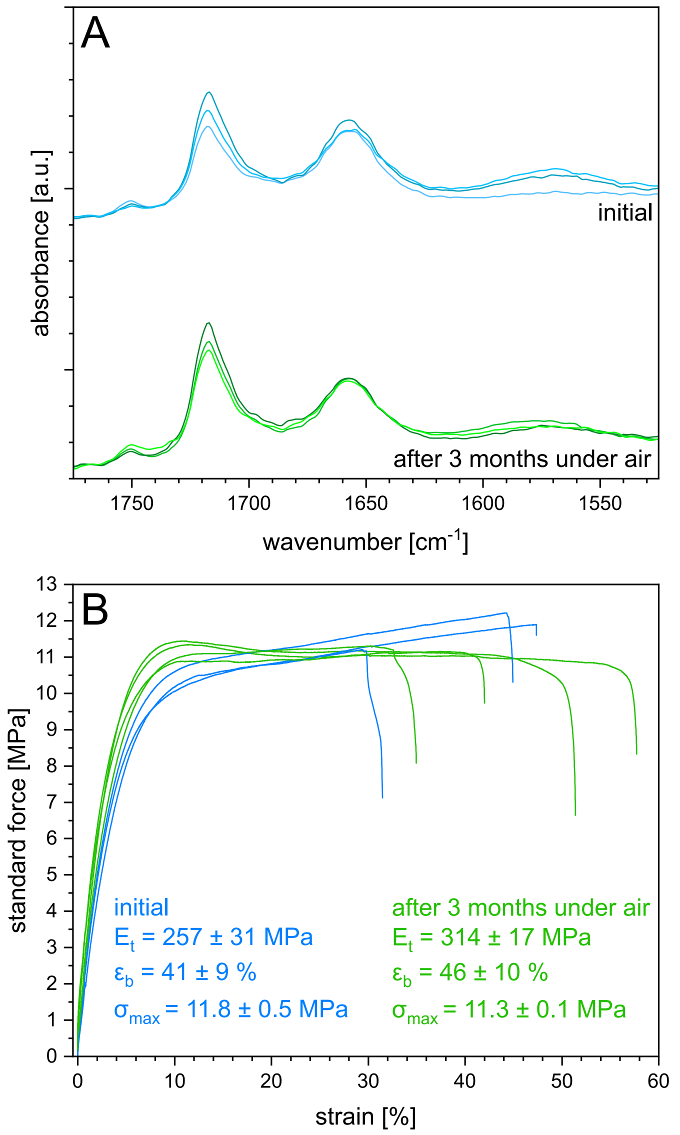

Figure S61. Comparison of imino-crosslinked LDPE specimens before and after exposure to air. **A:** Exemplary ATR-IR spectra. Spectra were normalized to the maximum at 2922 cm^{-1} and then shifted for clarity. **B:** Stress-strain curves.

2.8.2 Stability in water

A cylindrical sample of imino-crosslinked LDPE (1.4 cm length, 1.4 cm diameter, 1.9330 g total mass, gel fraction of 45 ± 1 wt.-%, 0.1 mol-% keto, 0.8 mol-% imino) was stirred in 4 L of distilled water at 20 °C for 4 weeks. The water was replaced every three days. After this time, the sample was dried and analyzed. Its mass decreased by 1.2 %. Its average gel fraction of 35 ± 3 wt.-% was determined on 8 individual slices. Note that each gel fraction value was determined on a complete slice so that the resulting average is a mean over the whole width of the crosslinked LDPE cylinder. ATR-IR spectra indicate considerable but not complete hydrolysis in the middle of the sample (0.4 mol-% keto, 0.7 mol-% imino content, *cf.* Figure S62). Spectra recorded on cross-sectional positions closer to the former contact surface with water (at ca. 2 mm depth) pointed towards a more pronounced but spatially very heterogenous degree of hydrolysis. The presence of considerable amounts of amino groups hampered quantification of keto and imino groups in these volume fractions.

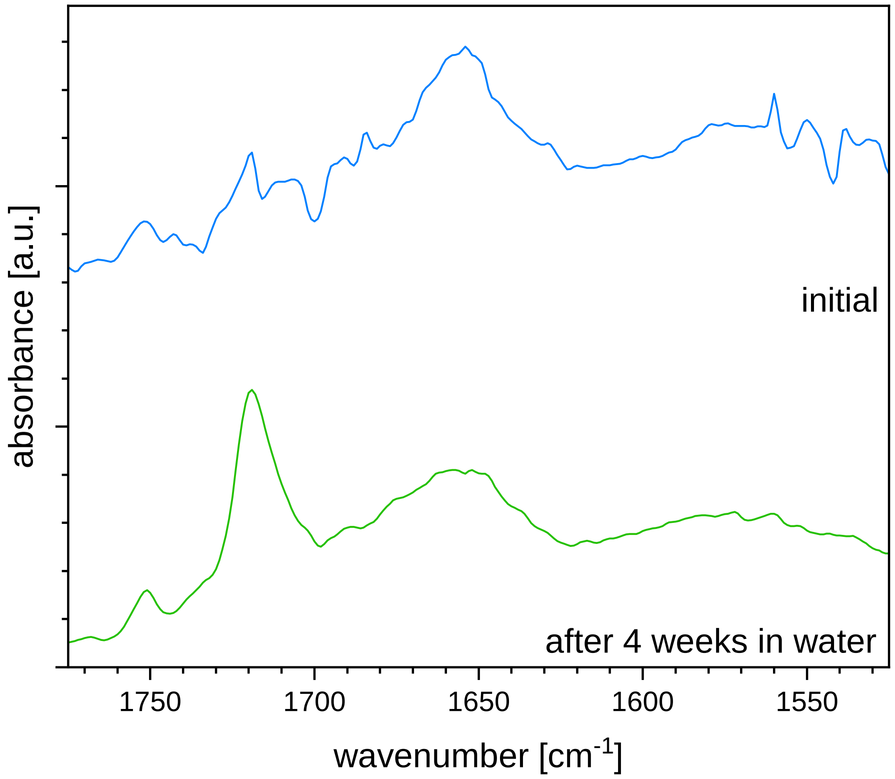

Figure S62. Comparative ATR-IR spectra of imino-crosslinked LDPE before and after 4 weeks in distilled water at 20 °C. Spectra were normalized to the maximum at 2922 cm^{-1} and then shifted for clarity.

3. References

1. Morgen, T. O.; Baur, M.; Göttker-Schnetmann, I.; Mecking, S. Photodegradable branched polyethylenes from carbon monoxide copolymerizatoin under benign conditions. *Nat. Commun.* 2020, *11*, 3693.
2. Baur, M.; Lin, F.; Morgen, T. O.; Odenwald, L.; Mecking, S. Polyethylene materials with in-chain ketones from non-alternating catalytic copolymerization. *Science* 2021, *374* (6567), 604-607.
3. Zhang, Y.; Mu, H.; Wang, X.; Pan, L.; Li, Y. Elaborate Tuning in Ligand Makes a Big Difference in Catalytic Performance: Bulky Nickel Catalysts for (Co)polymerization of Ethylene with Promising Vinyl Polar Monomers. *ChemCatChem* 2019, *11*, 2329-2340.
4. Ortmann, P.; Wimmer, F. P.; Mecking, S., Long-Spaced Polyketones from ADMET Copolymerizations as Ideal Models for Ethylene/CO Copolymers. *ACS Macro Letters* 2015**,** *4* (7), 704-707.
5. Soomro, S. S.; Cozzula, D.; Leitner, W.; Vogt, H.; Müller, T. E. The microstructure and melt properties of CO-ethylene copolymers with remarkably low CO content. *Polym. Chem.* 2014, *5*, 3831-3837.
6. Grau, E.; Broyer, J.-P.; Boisson, C.; Spitz, R.; Monteil, V. Unusual activation by solvent of the ethylene free radical polymerization. *Polym. Chem.* 2011, *2*, 2328-2333.
7. Brandolini, A. J.; Hills, D. D. *NMR Spectra of Polymers and Polymer Additives*, CRC Press, 2000.
